# The First Convergent Synthesis of 23,23-Difluoro-25-hydroxyvitamin D_3_ and Its 24-Hydroxy Derivatives: Preliminary Assessment of Biological Activities

**DOI:** 10.3390/molecules27165352

**Published:** 2022-08-22

**Authors:** Sayuri Mototani, Fumihiro Kawagoe, Kaori Yasuda, Hiroki Mano, Toshiyuki Sakaki, Atsushi Kittaka

**Affiliations:** 1 Faculty of Pharmaceutical Sciences, Teikyo University, 2-11-1 Kaga, Itabashi, Tokyo 173-8605, Japan; 2 Faculty of Engineering, Toyama Prefectural University, Imizu 939-0398, Toyama, Japan

**Keywords:** 23,23-difluorovitamin D_3_ analogues, synthesis, DAST, CYP24A1, VDR, vitamin D_3_ metabolite

## Abstract

In this paper, we report an efficient synthetic route for the 23,23-difluoro-25-hydroxyvitamin D_3_ (**5**) and its 24-hydroxylated analogues (**7**,**8**), which are candidates for the CYP24A1 main metabolites of **5**. The key fragments, 23,23-difluoro-CD-ring precursors (**9**–**11**), were synthesized starting from Inhoffen-Lythgoe diol (**12**), and introduction of the C23 difluoro unit to α-ketoester (**19**) was achieved using *N*,*N*-diethylaminosulfur trifluoride (DAST). Preliminary biological evaluation revealed that 23,23-F_2_-25(OH)D_3_ (**5**) showed approximately eight times higher resistance to CYP24A1 metabolism and 12 times lower VDR-binding affinity than its nonfluorinated counterpart 25(OH)D_3_ (**1**).

## 1. Introduction

**Scheme 1:**
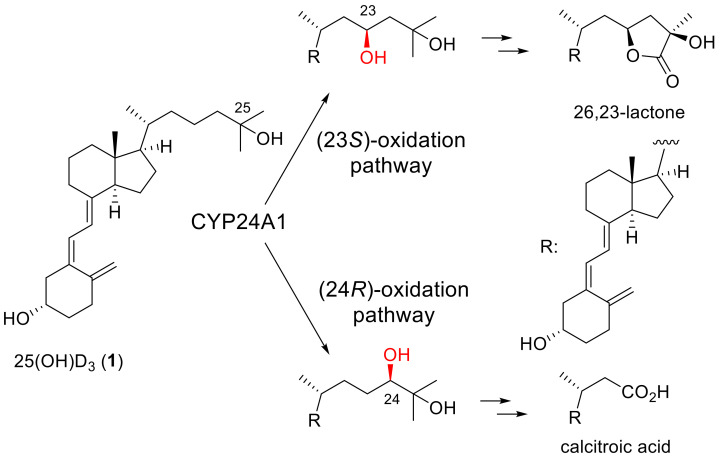
Main metabolic pathways of 25(OH)D_3_ (**1**) catalyzed by human CYP24A1.

**Figure 1:**
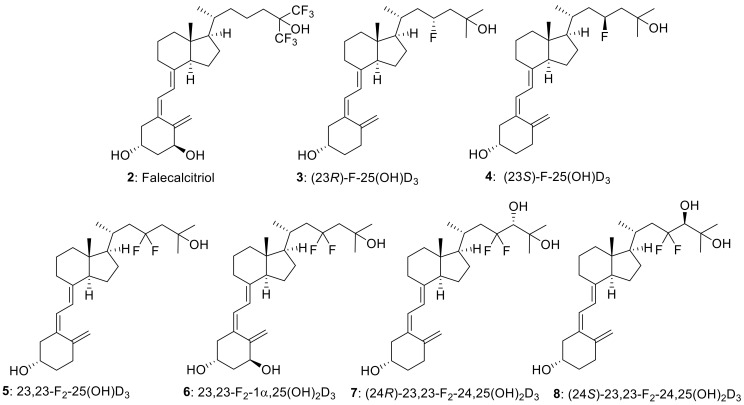
Structures of Falecalcitriol (**2**) and C23-fluorinated vitamin D_3_ analogues (**3**–**8**).

The introduction of fluorine atom(s) into biologically active compounds has been widely used in the development of pharmaceuticals, with the expected effects of increasing metabolic stability and improving binding affinity to target proteins [1,2,3,4]. Vitamin D_3_ is no exception. Fluorinated vitamin D_3_ analogues have been synthesized to extend the biological half-life and modulate the binding affinity to the vitamin D receptor (VDR), and their biological activities have been evaluated [5]. Among them, the side-chain fluorination of vitamin D_3_ has been vigorously pursued because hydroxylation of the side-chain C23 or C24 position and several subsequent oxidation steps by the metabolic enzyme CYP24A1 are the main deactivation processes of 25-hydroxyvitamin D_3_ [25(OH)D_3_] (**1**) (Scheme 1) [6,7,8]. Falecalcitriol (**2**), which has been clinically approved for the treatment of secondary hyperparathyroidism [9,10,11], is one such vitamin D_3_ analogue, and it contains a hexafluoroisopropanol unit in the side chain (Figure 1).

**Scheme 2:**
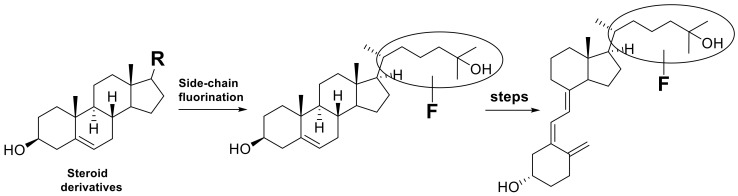
Synthetic strategy for side-chain fluorinated vitamin D_3_ analogues starting from sterols.

In recent years, new aspects of vitamin D_3_ function have been discovered [12,13,14,15], and syntheses of various vitamin D_3_ analogues have been carried out [16,17]. As a result, the importance of developing comprehensive and straightforward synthetic methods for fluorinated vitamin D_3_ analogues has increased. However, the synthetic methods reported so far are limited and mainly use sterol skeletons as starting materials, followed by photochemical transformation and thermal isomerization (Scheme 2) [5]. This strategy leads to a limited number of vitamin D derivatives even after multi-step synthesis with low chemical yields.

During our ongoing vitamin D_3_ research, we focused on the vitamin D_3_ side-chain C23 position and explored the efficient synthetic methodology for C23 fluorinated vitamin D_3_ analogues, in which we achieved the convergent stereoselective synthesis of (23*R*)-23-fluoro-25-hydroxyvitamin D_3_ [(23*R*)-F-25(OH)D_3_] (**3**) and its 23*S* isomer (**4**) through the corresponding CD-ring parts [18]. This time, we established an efficient synthetic route to 23,23-difluorovitamin D_3_ analogues. To the best of our knowledge, two 23,23-difluorovitamin D_3_ analogues: 23,23-difluoro-25-hydroxyvitamin D_3_ [23,23-F_2_-25(OH)D_3_] (**5**) and 23,23-difluoro-1α,25-dihydroxyvitamin D_3_ [23,23-F_2_-1α,25(OH)_2_D_3_] (**6**), were synthesized by Taguchi et al. in 1984 [19] and Nakada et al. in 1985 [20]. The synthetic route to **5** and **6** utilized the same strategy summarized in Scheme 2. They used a sterol-based compound as the starting material. After introducing the 23,23-difluoro unit into the side-chain, the B-ring was opened by photoirradiation, followed by thermal isomerization to afford **5**. They also prepared **6** by enzymatic 1α-hydroxylation of **5** [20].

**Figure 2:**
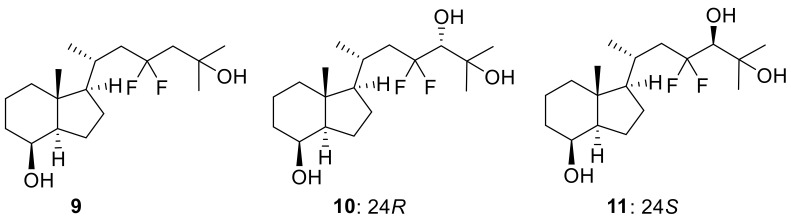
Structures of key CD-ring fragments (**9**–**11**) for the convergent method.

In this study, we herein report an alternative and efficient synthetic route to 23,23-difluorovitamin D_3_ analogues using a convergent method. First, 23,23-difluoro-CD-ring precursor (**9**) was prepared as a key intermediate in the synthesis of **5**. Next, we synthesized the 23,23-difluoro-24-hydroxy-CD-ring fragments (**10**,**11**) as substrates for 23,23-difluoro-24,25(OH)_2_D_3_ (**7**,**8**) as the possible CYP24A1 metabolites of 23,23-F_2_-25(OH)D_3_ (**5**) (Figure 2).

## 2. Results and Discussion

**Scheme 3:**
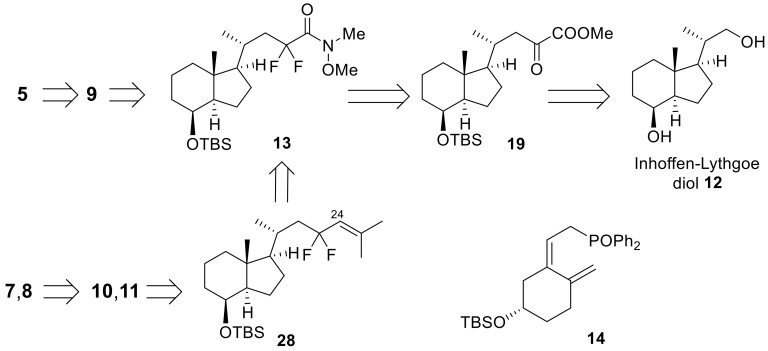
Retrosynthetic analysis of C23-fluorinated vitamin D_3_ analogues (**5**,**7**,**8**) using the convergent method.

The retrosynthetic analysis is shown in Scheme 3. The CD-rings (**9**–**11**) were synthesized from Inhoffen-Lythgoe diol (**12**), and the introduction of the difluoro unit to the C23 position was performed using a difluorination reaction of **19** with DAST [21,22,23]. Stereoselective introduction of the hydroxy group to the C24 position was achieved using Sharpless asymmetric dihydroxylation of **28** [24,25]. The A-ring phosphine oxide (**14**) was prepared from vitamin D_3_ [26].

**Scheme 4:**
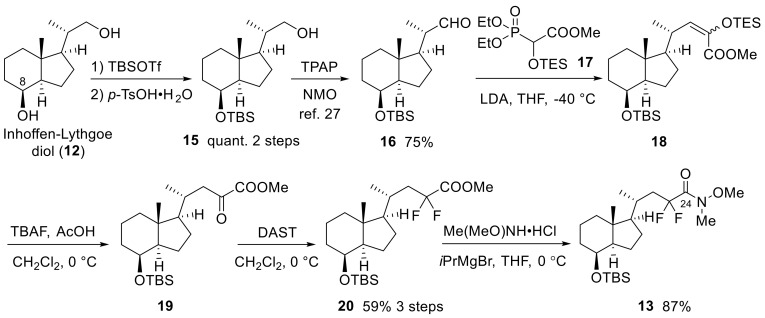
Preparation of 23,23-difluoro-24-amide-CD-ring (**13**) from Inhoffen-Lythgoe diol (**12**).

**Scheme 5:**
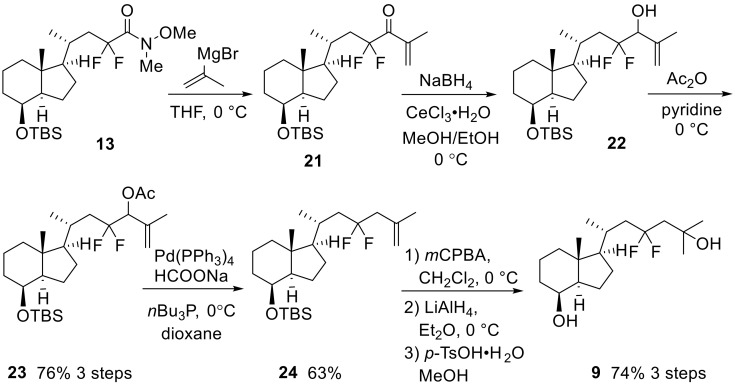
Preparation of 23,23-difluoro-CD-ring moiety (**9**) from **13**.

In Scheme 4 and Scheme 5, the synthesis of the 23,23-difluoro-CD-ring moiety (**9**) is described. Selective protection of the C8 secondary hydroxy group of **12** with a TBS group, followed by oxidation at the C22 primary alcohol by TPAP/NMO, afforded aldehyde (**16**) [27]. The aldehyde was subjected to the Horner-Emmons reagent (**17**) under basic conditions to create triethylsilyl enol ether (**18**), which was converted to α-ketoester (**19**) by selective desilylation of the silyl enol ether unit using TBAF in the presence of acetic acid [23]. The C23-difluoro unit was constructed using DAST toward α-ketoester (**19**), and the obtained difluoro methyl ester (**20**) was converted to a Weinreb amide (**13**) (Scheme 4).

Elongation of the side-chain progressed using isopropenyl Grignard reagent with Weinreb amide (**13**), and subsequent reduction of the obtained ketone (**21**) provided alcohol (**22**). After acetylation of **22**, a palladium-catalyzed regio-selective hydride reduction [28] yielded 25,26-alkene (**24**). Finally, epoxidation of the alkene moiety and reductive opening of the epoxide using LiAlH_4_, followed by desilylation at C8-OH afforded **9** (Scheme 5).

**Scheme 6:**
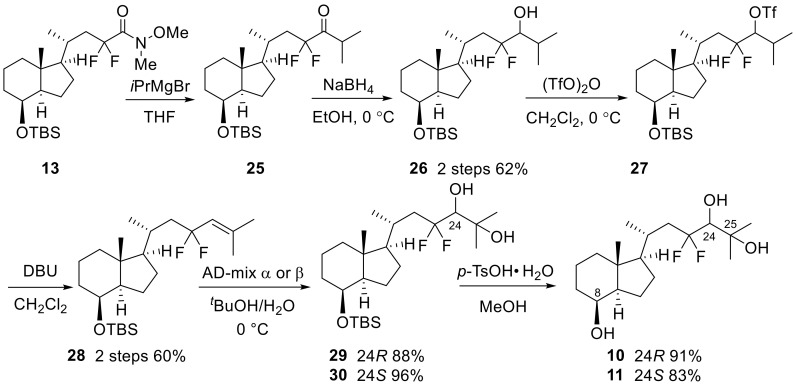
Stereoselective synthesis of 23,23-difluoro-24-hydroxy-CD-ring moieties (**10**,**11**).

**Figure 3:**
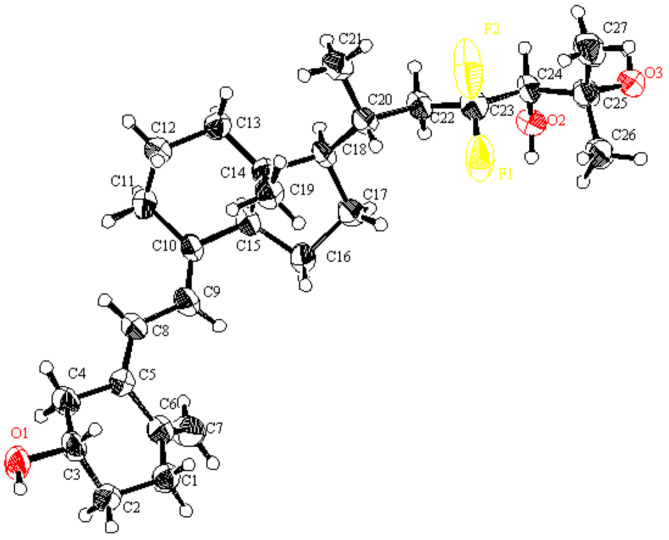
ORTEP drawings of **7**; Ellipsoid contour probability level = 50% (CCDC 2202393).

Next, we developed the synthetic route to two 23,23-difluoro-24-hydroxy-CD-ring moieties (**10**,**11**). As shown in Scheme 6, the synthesis started from Weinreb amide **13**, which was reacted with isopropyl magnesium bromide to provide ketone (**25**), followed by reduction with NaBH_4_ to afford alcohol (**26**). After trifluoromethanesulfonation of the alcohol, an E2 reaction under basic conditions afforded 24,25-olefin (**28**). Stereoselective hydroxylation was performed using Sharpless asymmetric dihydroxylation [24,25], followed by desilylation at C8-OH to develop the desired 8,24,25-trihydroxy-CD-rings (**10**,**11**). Later, the stereochemistry at the C24 position of **10** was determined to be the same as in compound **7** by X-ray crystallography (Figure 3).

**Scheme 7:**
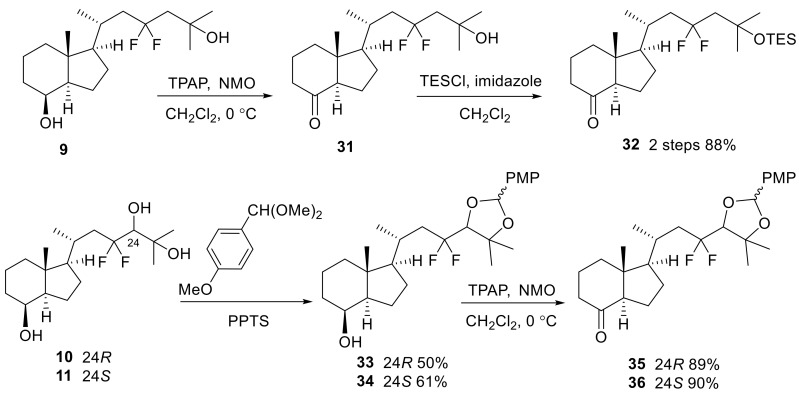
Synthesis of 23,23-difluoro-8-keto-CD-rings (**32**,**35**,**36**).

**Scheme 8:**
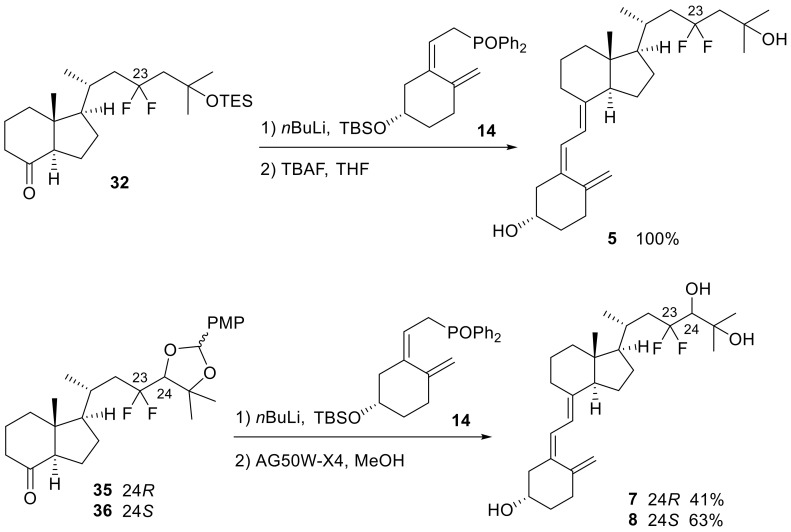
The Wittig−Horner coupling reaction between 8-keto-CD-rings (**32**,**35**,**36**) and the A-ring (**14**) and subsequent deprotection steps for **5**, **7**, and **8**.

The triene structures were constructed using 8-keto-CD-rings (**32**,**35**,**36**) and the A-ring (**14**). The 8-keto-CD-ring (**32**) was synthesized via the oxidation of **9** and subsequent protection of the C25-OH group with TES. For the synthesis of the 8-keto-CD-rings (**35**,**36**), both C24 and C25 hydroxy groups of **10** and **11** were protected with *p*-methoxybenzylidene acetal that provided inseparable diastereomeric mixtures, respectively, followed by oxidation of the C8-OH group using TPAP/NMO to generate **35** and **36** (Scheme 7). The coupling reaction between the 8-keto-CD-rings (**32**,**35**,**36**) and A-ring phosphine oxide (**14**) was performed using the Wittig–Horner reaction, followed by deprotection to yield the desired products (**5**,**7**,**8**) (Scheme 8).

The structure of **7,** including stereochemistry at the C24 position, was clarified with X-ray crystallography (Figure 3).

### Biological Evaluation

The metabolism of 23,23-F_2_-25(OH)D_3_ (**5**) by hCYP24A1 and the binding affinity for hVDR were evaluated (Table 1 and Table 2). 23,23-F_2_-25(OH)D_3_ (**5**) showed eight times higher resistance to CYP24A1 metabolism than its nonfluorinated counterpart 25(OH)D_3_ (**1**). We previously reported that 24,24-difluoro-25-hydroxyvitamin D_3_ showed nearly the same metabolic resistance toward CYP24A1 [23,25].

For hVDR binding affinity, Ikekawa and coworkers reported that 23,23-F_2_-1α,25(OH)_2_D_3_ (**6**) was seven times less active than the natural hormone 1α,25(OH)_2_D_3_ [20]. In our experiments, 23,23-F_2_-25(OH)D_3_ (**5**) possessed approximately 12 times lower binding affinity than 25(OH)D_3_ (**1**) (Table 2). On the contrary, 24,24-difluoro-25-hydroxyvitamin D_3_ showed 1.8-times higher binding affinity than 25(OH)D_3_ (**1**) [23,25]. These results suggest that the fluorine atoms at the C23 position markedly impair the binding affinity to hVDR.

## 3. Conclusions

In summary, we developed a novel and efficient synthetic route to 23,23-difluoro-25-hydroxyvitamin D_3_ analogues (**5**,**7**,**8**) via the key fragments 23,23-difluoro-CD-rings (**9**–**11**). The 23,23-difluoro unit was constructed using DAST toward α-ketoester (**19**). Preliminary biological evaluation revealed that 23,23-F_2_-25(OH)D_3_ (**5**) exhibits higher resistance against CYP24A1 metabolism and lower binding affinity for hVDR than 25(OH)D_3_ (**1**).

## 4. Experimental Section

^1^H and ^13^C-NMR spectra were recorded on JEOL AL-400 NMR (400 MHz) and ECP-600 NMR (600 MHz) spectrometers (Tokyo, Japan). ^1^H-NMR spectra were referenced with (CH_3_)_4_Si (δ 0.00 ppm) or CHCl_3_ (δ 7.26 ppm) as an internal standard. ^13^C-NMR spectra were referenced with deuterated solvent (δ 77.0 ppm for CDCl_3_). IR spectra were recorded on a JASCO FT-IR-800 Fourier transform infrared spectrophotometer (Tokyo, Japan). High-resolution mass spectra were obtained on a SHIMADZU LCMS-IT-TOF mass spectrometer (Kyoto, Japan) with an electrospray ionization (ESI) method or atmospheric pressure chemical ionization (APCI). Optical rotations were measured on a JASCO DIP-370 digital polarimeter (Tokyo, Japan). Column chromatography was performed on silica gel 60N (Kanto Chemical Co., Inc., 40–50 μm, Tokyo, Japan) or silica gel 60 (Merck, 0.040–0.063 mm, Tokyo, Japan). All experiments were performed under anhydrous conditions under an atmosphere of argon unless otherwise stated. The supporting information of ^1^H and ^13^C NMR spectra of all new compounds: **5**, **7**–**11**, **13**, **20**, **23**, **24**, **26**, **28**–**30**, and **32**–**36**, as well as ^19^F NMR spectra of compounds: **5**, **7**, and **8** is available at the link in Supplementary Materials.

*Methyl (R)-4-{(1R,3aR,4S,7aR)-4-[(tert-butyldimethylsilyl)oxy]-7a-methyloctahydro-1H-inden-1-yl}-2,2-difluoropentanoate* (**20**)

To the solution of Horner–Emmons reagent (**17**) (2.35 g, 7.24 mmol) in THF (3.5 mL), LDA (lithium diisopropylamide) (3.5 mL, 2 M THF/heptane/ethylbenzene solution, 1.75 mmol) was added at −40 °C; the mixture was stirred at the same temperature for 20 min, and a solution of **16** [27] (977.0 mg, 3.01 mmol) in THF (3.5 mL) was added. The reaction mixture was stirred at 0 °C for 20 min. After the reaction was quenched with H_2_O at 0 °C, the mixture was extracted with EtOAc three times. The organic layer was dried over Na_2_SO_4_, filtered, and concentrated. The crude residue was roughly purified by flash column chromatography on silica gel (hexanen:EtOAc = 3:1, 1% Et_3_N) to obtain the crude coupling product **18**, and it was used for the next reaction without further purification.

To the above coupling product **18** in CH_2_Cl_2_ (7 mL), AcOH (1.03 mL) and tetrabutylammonium fluoride (4.8 mL, 1 M THF solution, 4.8 mmol) were added at 0 °C under air, and the mixture was stirred at room temperature for 15 min. After the reaction was quenched with H_2_O at room temperature, the mixture was extracted with CH_2_Cl_2_ three times. The organic layer was dried over Na_2_SO_4_, filtered, and concentrated. The residue was partially purified by flash column chromatography on silica gel (hexane:EtOAc = 10:1) to obtain a crude residue of **19** (1.10 g).

To the solution of the above crude residue of **19** (1.10 g), CH_2_Cl_2_ (6.5 mL) was slowly added to *N*,*N*-diethylaminosulfur trifluoride (DAST) (2.8 mL, 3.4 g, 21.1 mmol) at 0 °C, and the mixture was stirred at room temperature for 16 h. The mixture was cooled to 0 °C, and MeOH and H_2_O were slowly added. The mixture was extracted with CH_2_Cl_2_ three times. The organic layer was dried over Na_2_SO_4_, filtered, and concentrated. The residue was partially purified by flash column chromatography on silica gel (hexane:EtOAc = 10:1) to obtain **20** (747.0 mg, 59%, three steps) as a colorless oil.

**20**: ${\left[\alpha \right]}_{D}^{27}$ +36.5 (c 1.90, CHCl_3_); IR (neat) 1774, 1471, 1253, 1097, 1077, 1029, 838 cm^−1^; ^1^H-NMR (600 MHz, CDCl_3_) δ −0.01 (s, 3H), 0.01 (s, 3H), 0.88 (s, 9H), 0.90 (d, *J* = 5.4 Hz, 3H), 0.91 (s, 3H), 1.03 (q, *J* = 9.6 Hz, 1H), 1.10 (td, *J* = 3.6, 13.2 Hz, 1H), 1.17–1.27 (m, 2H), 1.31–1.38 (m, 2H), 1.43–1.49 (m, 1H), 1.51–1.59 (m, 2H), 1.65–1.67 (m, 1H), 1.74–1.84 (m, 2H), 1.88–1.98 (m, 2H), 2.04–2.16 (m, 1H), 3.86 (s, 3H), 3.98–4.00 (m, 1H); ^13^C-NMR (150 MHz, CDCl_3_) δ −5.2, −4.8, 13.7, 17.6, 18.0, 18.3, 23.0, 25.8, 27.0, 27.1, 31.1 (t, *J* = 22.3 Hz), 34.4, 34.5, 40.6, 42.1, 53.0, 53.2, 56.0, 69.4, 116.8 (t, *J* = 248.5 Hz), 165.0 (t, *J* = 33.8 Hz); HRMS (ESI^−^) calcd for C_22_H_39_O_3_F_2_Si [M − H]^−^ 417.2642, found 417.2662.

*(R)-4-{(1R,3aR,4S,7aR)-4-[(tert-Butyldimethylsilyl)oxy]-7a-methyloctahydro-1H-inden-1-yl}-2,2-difluoro-N-methoxy-N-methylpentanamide* (**13**)

To the solution of compound **20** (1.01 g, 2.41 mmol) and Me(MeO)NH·HCl (934.3 mg, 9.58 mmol) in THF (20 mL), isopropyl magnesium chloride (19.0 mL, 1 M in THF, 19.0 mmol) was added at 0 °C, and the mixture was stirred at the same temperature for 23 h. After the reaction was quenched with water and aqueous saturated NH_4_Cl, the mixture was extracted with EtOAc three times. The organic layer was dried over Na_2_SO_4_, filtered, and concentrated. The residue was purified by flash column chromatography on silica gel (hexane:EtOAc = 6:1) to obtain **13** (933.9 mg, 87%) as a colorless oil.

**13**: ${\left[\alpha \right]}_{D}^{27}$ +39.9 (c 3.47, CHCl_3_); IR (neat) 1685, 1464, 1379, 1252, 1085, 1039, 984, 837 cm^−1^; ^1^H-NMR (600 MHz, CDCl_3_) δ −0.02 (s, 3H), 0.00 (s, 3H), 0.88 (s, 9H), 0.92 (s, 3H), 1.02 (d, *J* = 6.6 Hz, 3H), 1.06–1.13 (m, 2H), 1.21–1.26 (m, 2H), 1.30–1.37 (m, 3H), 1.51–1.59 (m, 1H), 1.64–1.66 (m, 1H), 1.74–1.87 (m, 4H), 1.94–1.96 (m, 1H), 2.22–2.32 (m, 1H) 3.24 (brs, 3H), 3.72 (s, 3H), 3.98–3.99 (m, 1H); ^13^C-NMR (150 MHz, CDCl_3_) δ −5.2, −4.8, 13.5, 17.6, 18.0, 20.2, 23.0, 25.8, 27.4, 30.6, 33.1, 34.3, 40.1 (t, *J* = 20.8 Hz), 40.6, 42.2, 53.1, 56.9, 61.9, 69.4, 118.6 (t, *J* = 249.2 Hz), 164.7 (t, *J* = 27.9 Hz); HRMS (ESI^+^) calcd for C_23_H_43_NO_3_F_2_SiNa [M + Na]^+^ 470.2872, found 470.2856.

*(6R)-6-{(1R,3aR,4S,7aR)-4-[(tert-Butyldimethylsilyl)oxy]-7a-methyloctahydro-1H-inden-1-yl}-4,4-difluoro-2-methylhept-1-en-3-yl acetate* (**23**)

To the solution of compound **13** (251.5 mg, 0.56 mmol) in THF (20 mL), isopropenyl magnesium bromide (2.25 mL, 0.5 M in THF, 1.12 mmol) was added at 0 °C, and the mixture was stirred at room temperature for 1 h. After the reaction was quenched with water, the mixture was extracted with EtOAc three times. The organic layer was dried over Na_2_SO_4_, filtered, and concentrated. The residue was purified by flash column chromatography on silica gel (hexane:EtOAc = 20:1) to obtain the crude product **21**.

NaBH_4_ (45.1 mg, 1.19 mmol) was added to the solution of the above crude **21** and CeCl_3_·6H_2_O in EtOH (3 mL) and MeOH (3 mL) at 0 °C, and the mixture was stirred at the same temperature for 5 min. After the reaction was quenched with water, the mixture was extracted with EtOAc three times. The organic layer was dried over Na_2_SO_4_, filtered, and concentrated. The crude **22** was used for the next reaction without further purification.

*N*,*N*-Dimethyl-4-aminopyridine (464.5 mg, 3.68 mmol) and Ac_2_O (1.85 g, 2 mL, 18.1 mmol) were added to the solution of the crude **22** in pyridine (4 mL) at 0 °C, and the mixture was stirred at room temperature for 10 min. After the reaction was quenched with water at room temperature, the mixture was extracted with EtOAc three times. The organic layer was dried over Na_2_SO_4_, filtered, and concentrated. The residue was purified by flash column chromatography on silica gel (hexane:EtOAc = 20:1) to obtain **23** (mixture of diastereomers) (201.8 mg, 76%, three steps) as a colorless oil.

**23**: IR (neat) 1755, 1468, 1375, 1235, 1085, 1039, 841, 775 cm^−1^; ^1^H-NMR (600 MHz, CDCl_3_) δ −0.01 (s, 3H), 0.01 (s, 3H), 0.88 (s, 9H), 0.94 (s, 3H), 1.04–1.15 (m, 5H), 1.17–1.27 (m, 2H), 1.30–1.38 (m, 3H), 1.46–1.60 (m, 2H), 1.65–1.67 (m, 1H), 1.71–2.03 (m, 7H), 2.13–2.14 (m, 3H), 3.98–4.00 (m, 1H), 5.10–5.11 (m, 2H), 5.25–5.32 (m, 1H); ^13^C-NMR (150 MHz, CDCl_3_) δ −5.2, −4.8, 13.5, 13.5, 17.6, 18.0, 19.4, 19.7, 20.4, 20.5, 20.8, 23.0, 25.8, 27.5, 27.5, 30.3, 30.4, 34.3, 38.5 (t, *J* = 22.3 Hz), 38.7 (t, *J* = 22.3 Hz), 40.7, 42.2, 53.1, 57.0, 57.0, 69.4, 76.3 (t, *J* = 28.7 Hz), 76.8 (t, *J* = 27.3 Hz), 117.0, 117.4, 122.9 (t, *J* = 247.1 Hz), 122.9 (t, *J* = 246.4 Hz), 138.4, 138.5, 169.3, 169.4; HRMS (ESI^+^) calcd for C_26_H_50_O_3_NF_2_SiNa [M + Na]^+^ 490.3523, found 490.3543.

*tert-Butyl({(1R,3aR,4S,7aR)-1-[(R)-4,4-difluoro-6-methylhept-6-en-2-yl]-7a-methyloctahydro-1H-inden-4-yl}oxy)dimethylsilane* (**24**)

To a solution of sodium formate (155.8 mg, 2.29 mmol) and Pd(PPh_3_)_4_ (423.2 mg, 0.37 mmol) in dioxane (1.5 mL), *n*Bu_3_P (345.5 mg, 427.0 μL, 1.77 mmol) was added at room temperature, and the mixture was stirred at 90 °C for 10 min. Acetate **23** (201.8 mg, 0.43 mmol) was dissolved in dioxane (1.5 mL), and the solution was added to the mixture. After being stirred at the same temperature for 17 h, the reaction mixture was quenched with H_2_O at room temperature. The mixture was extracted with EtOAc three times. The organic layer was washed with brine, dried over Na_2_SO_4_, filtered, and concentrated. The residue was partially purified by flash column chromatography on silica gel (hexane:EtOAc = 100:1), and repurification by flash column chromatography on silica gel (hexane only) provided **24** (111.9 mg, 63%) as a colorless oil.

**24**: ${\left[\alpha \right]}_{D}^{27}$ +36.0 (c 0.69, CHCl_3_); IR (neat) 1471, 1379, 1255, 1166, 1085, 1023, 837, 775 cm^−1^; ^1^H-NMR (600 MHz, CDCl_3_) δ 0.00 (s, 3H), 0.01 (s, 3H), 0.89 (s, 9H), 0.95 (s, 3H), 1.04 (d, *J* = 7.2 Hz, 3H), 1.07 (q, *J* = 9.6 Hz, 1H), 1.12 (td, *J* = 3.6, 13.2 Hz, 1H), 1.21–1.27 (m, 2H), 1.31–1.38 (m, 3H), 1.46–1.61 (m, 2H), 1.66–1.68 (m, 1H), 1.75–1.86 (m, 6H), 1.89–2.00 (m, 2H), 2.48–2.59 (m, 2H), 3.99–4.00 (m, 1H), 4.84 (brs, 1H), 4.59 (brs, 1H); ^13^C-NMR (150 MHz, CDCl_3_) δ −5.2, −4.8, 13.5, 17.6, 18.0, 20.3, 23.0, 23.3, 25.8, 27.5, 30.9, 34.4, 40.7, 41.7 (t, *J* = 23.0 Hz), 42.2, 45.6 (t, *J* = 25.9 Hz), 53.1, 57.1, 69.4, 116.3, 125.2 (t, *J* = 242.0 Hz), 138.7; HRMS (ESI^+^) calcd for C_24_H_44_OF_2_SiNa [M + Na]^+^ 437.3022, found 437.3033.

*(1R,3aR,4S,7aR)-1-[(2R)-4,4-Difluoro-6-hydroxy-6-methylheptan-2-yl]-7a-methyloctahydro-1H-inden-4-ol* (**9**)

*m*CPBA (286.1 mg, 1.66 mmol) was added to the mixture of **24** (111.9 mg, 0.27 mmol) and NaHCO_3_ (131.9 mg, 1.57 mmol) in CH_2_Cl_2_ (2 mL) at 0 °C, and the mixture was stirred at the same temperature for 100 min under air. After the reaction was quenched with H_2_O and saturated aqueous NaHCO_3_ at room temperature, the mixture was extracted with CH_2_Cl_2_ three times. The organic layer was washed with brine, dried over Na_2_SO_4_, filtered, and concentrated. The residue was purified by flash column chromatography on silica gel (hexane:EtOAc = 20:1) to obtain the crude epoxide.

LiAlH_4_ (14.0 mg, 0.37 mmol) was added to the solution of the above crude epoxide in Et_2_O (3 mL) at 0 °C, and the mixture was stirred at the same temperature for 15 min, and then at room temperature for 20 min. After the reaction was quenched with MeOH, water, and saturated aqueous potassium sodium tartrate, the mixture was extracted with EtOAc three times. The organic layer was washed with brine, dried over Na_2_SO_4_, filtered, and concentrated. The crude alcohol was used for the next reaction without further purification.

*p*-Toluenesulfonic acid monohydrate (981.0 mg, 5.16 mmol) was added to the solution of the above crude alcohol in MeOH (20 mL), and the mixture was stirred at room temperature for 25 h under air. After the reaction was quenched with H_2_O and saturated aqueous NaHCO_3_ at room temperature, the mixture was extracted with EtOAc three times. The organic layer was washed with brine, dried over Na_2_SO_4_, filtered, and concentrated. The residue was purified by flash column chromatography on silica gel (hexane:EtOAc = 2:1) to obtain **9** (63.8 mg, 74%, three steps) as a colorless oil.

**9**: ${\left[\alpha \right]}_{D}^{27}$ +26.9 (c 2.20, CHCl_3_); IR (neat) 3412, 1471, 1375, 1170, 987, 864 cm^−1^; ^1^H-NMR (600 MHz, CDCl_3_) δ 0.97 (s, 3H), 1.04 (d, *J* = 6.6 Hz, 3H), 1.08–1.18 (m, 2H), 1.23–1.38 (m, 8H), 1.39–1.49 (m, 3H), 1.54–1.88 (m, 8H), 1.92–2.12 (m, 4H), 4.06–4.08 (m, 1H); ^13^C-NMR (150 MHz, CDCl_3_) δ 13.3, 17.4, 20.3, 22.4, 27.5, 30.2, 30.4, 31.0, 33.5, 40.3, 41.9, 44.1 (t, *J* = 23.0 Hz), 48.6 (t, *J* = 23.0 Hz), 52.7, 56.8, 69.3, 69.9, 126.6 (t, *J* = 241.3 Hz); HRMS (ESI^+^) calcd for C_18_H_32_O_2_F_2_Na [M + Na]^+^ 341.2263 found 341.2249.

*(6R)-6-{(1R,3aR,4S,7aR)-4-[(tert-Butyldimethylsilyl)oxy]-7a-methyloctahydro-1H-inden-1-yl}-4,4-difluoro-2-methylheptan-3-ol* (**26**)

To the solution of compound **13** (933.9 mg, 2.09 mmol) in THF (10 mL), isopropyl magnesium chloride (6.3 mL, 1 M in THF, 6.26 mmol) was added at 0 °C, and the mixture was stirred at room temperature for 1 h. After the reaction was quenched with water and aqueous saturated NH_4_Cl at 0 °C, the mixture was extracted with EtOAc three times. The organic layer was dried over Na_2_SO_4_, filtered, and concentrated. The residue was purified by flash column chromatography on silica gel (hexane:EtOAc = 10:1) to obtain the crude product **25**.

NaBH_4_ (45.1 mg, 1.19 mmol) was added to the solution of the above crude **25** in EtOH (3 mL) at 0 °C, and the mixture was stirred at the same temperature for 5 min. After the reaction was quenched with water, the mixture was extracted with EtOAc three times. The organic layer was washed with brine, dried over Na_2_SO_4_, filtered, and concentrated. The residue was purified by flash column chromatography on silica gel (hexane:EtOAc = 10:1) to obtain **26** (mixture of diastereomers) (563.4 mg, 62%, two steps) as a colorless oil.

**26**: IR (neat) 3442, 1471, 1367, 1255, 1166, 1085, 1023, 837, 775 cm^−1^; ^1^H-NMR (600 MHz, CDCl_3_) δ −0.01 (s, 3H), 0.01 (s, 3H), 0.89 (s, 9H), 0.95–1.02 (m, 8H), 1.04–1.15 (m, 4H), 1.23–1.38 (m, 6H), 1.49–1.67 (m, 3H), 1.77–2.12 (m, 7H), 3.38–3.45 (m, 1H), 3.99–4.00 (m, 1H); ^13^C-NMR (150 MHz, CDCl_3_) δ −5.2, −4.8, 13.5, 13.6, 17.0, 17.5, 17.6, 18.0, 20.2, 20.3, 20.5, 20.7, 23.0, 25.8, 27.6, 27.6, 28.5, 28.6, 30.3, 30.5, 34.4, 38.4 (t, *J* = 21.5 Hz), 40.7, 40.7, 42.2, 53.1, 57.1, 69.4, 77.4 (t, *J* = 27.3 Hz), 77.6 (t, *J* = 28.9 Hz), 125.3 (t, *J* = 244.9 Hz), 125.5 (t, *J* = 244.9 Hz); HRMS (ESI^+^) calcd for C_24_H_47_O_2_F_2_Si [M + H]^+^ 433.3308, found 433.3290.

*tert-Butyl({(1R,3aR,4S,7aR)-1-[(R)-4,4-difluoro-6-methylhept-5-en-2-yl]-7a-methyloctahydro-1H-inden-4-yl}oxy)dimethylsilane* (**28**)

Trifluoromethanesulfonic anhydride (366.8 mg, 213 μL, 1.30 mmol) was added to the solution of **26** (467.3 mg, 1.08 mmol) and 2,6-di-*tert*-butylpyridine (464.5 mg, 3.68 mmol) in CH_2_Cl_2_ (4 mL) at 0 °C, and the mixture was stirred at the same temperature for 75 min. After the reaction was quenched with water and aqueous saturated NH_4_Cl at room temperature, the mixture was extracted with EtOAc three times. The organic layer was dried over Na_2_SO_4_, filtered, and concentrated. The crude **27** was used for the next reaction without further purification.

1,8-Diazabicyclo [5.4.0]undec-7-ene (DBU) (600 μL) was added to the solution of the above crude **27** in CH_2_Cl_2_ (4 mL) at room temperature and the mixture was stirred at the same temperature for 15 h. After the reaction was quenched with water and aqueous saturated NH_4_Cl at room temperature, the mixture was extracted with EtOAc three times. The organic layer was dried over Na_2_SO_4_, filtered, and concentrated. The residue was purified by flash column chromatography on silica gel (hexane only) to obtain **28** (269.8 mg, 60%, two steps) as a colorless oil.

**28**: ${\left[\alpha \right]}_{D}^{27}$ +59.4 (c 0.79, CHCl_3_); IR (neat) 1468, 1375, 1255, 1166, 1085, 1027, 984, 837, 771 cm^−1^; ^1^H-NMR (600 MHz, CDCl_3_) δ −0.01 (s, 3H), 0.01 (s, 3H), 0.89 (s, 9H), 0.94 (s, 3H), 1.03 (d, *J* = 6.6 Hz, 3H), 1.07 (q, *J* = 9.6 Hz, 1H), 1.10 (td, *J* = 3.6, 13.2 Hz, 1H), 1.21–1.27 (m, 2H), 1.31–1.38 (m, 3H), 1.53–1.84 (m, 12H), 1.95–2.06 (m, 2H), 3.99–4.00 (m, 1H), 5.32 (t, *J* = 8.1 Hz, 1H); ^13^C NMR (150 MHz, CDCl_3_) δ −5.2, −4.8, 13.5, 17.6, 18.0, 19.1, 20.4, 23.0, 25.8, 26.5, 27.6, 31.2, 34.4, 40.7, 42.2, 44.2 (t, *J* = 25.1 Hz), 53.1, 57.0, 69.4, 121.6 (t, *J* = 26.5 Hz), 123.2 (t, *J* = 238.5 Hz), 141.3 (t, *J* = 5.8 Hz); HRMS (ESI^+^) calcd for C_24_H_44_OF_2_SiNa [M + Na]^+^ 437.3022, found 437.3004.

*(3R,6R)-6-{(1R,3aR,4S,7aR)-4-[(tert-Butyldimethylsilyl)oxy]-7a-methyloctahydro-1H-inden-1-yl}-4,4-difluoro-2-methylheptane-2,3-diol* (**29**)

The mixture of AD-mix α (605.3 mg) in *t*BuOH (4 mL) and H_2_O (4 mL) was stirred at 0 °C for 20 min, and **28** (55.9 mg, 0.13 mmol) was added to the mixture at 0 °C with stirring at the same temperature for 4 h, and then at 4 °C for 20 h under air. After the reaction was quenched with water, the mixture was extracted with EtOAc three times. The organic layer was dried over Na_2_SO_4_, filtered, and concentrated. The residue was purified by flash column chromatography on silica gel (hexane:EtOAc = 1:1) to obtain **29** (53.4 mg, 88%) as a colorless oil.

**29**: ${\left[\alpha \right]}_{D}^{27}$ +35.0 (c 2.71, CHCl_3_); IR (neat) 3408, 1471, 1375, 1255, 1166, 1085, 1023, 833, 775 cm^−1^; ^1^H-NMR (600 MHz, CDCl_3_) δ −0.01 (s, 3H), 0.01 (s, 3H), 0.88 (s, 9H), 0.95 (s, 3H), 1.06 (d, *J* = 7.2 Hz, 3H), 1.09–1.16 (m, 2H), 1.22–1.38 (m, 11H), 1.53–1.67 (m, 3H), 1.67–1.88 (m, 3H), 1.95–2.00 (m, 2H), 2.26 (ddd, *J* = 7.8, 13.8, 34.2 Hz, 1H), 2.79 (d, *J* = 6.6 Hz, 1H), 3.41 (ddd, *J* = 5.4, 7.8, 19.2 Hz, 1H), 3.99–3.99 (m, 1H); ^13^C-NMR (150 MHz, CDCl_3_) δ −5.2, −4.8, 13.5, 17.6, 18.0, 21.1, 23.0, 25.6, 25.8, 27.6, 28.2, 30.1, 34.4, 39.5 (t, *J* = 22.3 Hz), 40.7, 42.2, 53.2, 57.1, 69.4, 72.1, 77.5 (t, *J* = 27.3 Hz), 126.3 (t, *J* = 245.6 Hz); HRMS (ESI^−^) calcd for C_25_H_47_O_5_F_2_Si [M + HCOO]^−^ 439.3166, found 439.3170.

*(3S,6R)-6-{(1R,3aR,4S,7aR)-4-[(tert-Butyldimethylsilyl)oxy]-7a-methyloctahydro-1H-inden-1-yl}-4,4-difluoro-2-methylheptane-2,3-diol* (**30**)

The mixture of AD-mix β (704.8 mg) in *t*BuOH (4 mL) and H_2_O (4 mL) was stirred at 0 °C for 30 min, and **28** (51.8 mg, 0.13 mmol) was added to the mixture at 0 °C with stirring at the same temperature for 5 h, and then at 4 °C for 19 h under air. After the reaction was quenched with water, the mixture was extracted with EtOAc three times. The organic layer was washed with brine, dried over Na_2_SO_4_, filtered, and concentrated. The residue was purified by flash column chromatography on silica gel (hexane:EtOAc = 2:1) to obtain **30** (53.8 mg, 96%) as a white powder.

**30**: ${\left[\alpha \right]}_{D}^{27}$ +44.7 (c 0.30, CHCl_3_); IR (neat) 3431, 1471, 1375, 1255, 1162, 1081, 1019, 837, 771 cm^−1^; ^1^H-NMR (400 MHz, CDCl_3_) 

δ −0.01 (s, 3H), 0.00 (s, 3H), 0.88 (s, 9H), 0.96 (s, 3H), 1.03 (d, *J* = 6.4 Hz, 3H), 1.07–1.16 (m, 2H), 1.21–1.39 (m, 11H), 1.52–1.86 (m, 6H), 1.95–2.19 (m, 3H), 2.84 (d, *J* = 7.6 Hz, 1H), 3.42 (ddd, *J* = 3.6, 8.0, 21.5 Hz, 1H), 3.99–3.99 (m, 1H); ^13^C-NMR (100 MHz, CDCl_3_) δ −5.2, −4.8, 13.6, 17.6, 18.0, 20.2, 23.0, 25.7, 25.8, 27.5, 28.2, 30.6, 34.4, 39.9 (t, *J* = 21.9 Hz), 40.7, 42.2, 53.1, 57.1, 69.4, 72.3, 76.6 (t, *J* = 28.2 Hz), 126.8 (t, *J* = 246.0 Hz); HRMS (ESI^−^) calcd for C_25_H_47_O_5_F_2_Si [M + HCOO]^−^ 439.3166, found 439.3164.

*(3R,6R)-4,4-Difluoro-6-[(1R,3aR,4S,7aR)-4-hydroxy-7a-methyloctahydro-1H-inden-1-yl]-2-methylheptane-2,3-diol* (**10**)

To the solution of **29** (53.4 mg, 0.12 mmol) in MeOH (10 mL), *p*-toluenesulfonic acid monohydrate (222.8 mg, 1.17 mmol) was added, and the mixture was stirred at room temperature for 39 h under air. After the reaction was quenched with H_2_O and saturated aqueous NaHCO_3_ at room temperature, the mixture was extracted with CH_2_Cl_2_ three times. The organic layer was dried over Na_2_SO_4_, filtered, and concentrated. The residue was purified by flash column chromatography on silica gel (hexane:EtOAc = 1:2) to obtain **10** (36.1 mg, 91%, in two steps) as a white powder.

**10**: ${\left[\alpha \right]}_{D}^{27}$ +28.7 (c 2.34, CHCl_3_); IR (neat) 3415, 1471, 1371, 1267, 1162, 1073, 991, 741 cm^−1^; ^1^H-NMR (600 MHz, CDCl_3_) δ 0.96 (s, 3H), 1.05 (d, *J* = 7.2 Hz, 3H), 1.11–1.17 (m, 2H), 1.27–1.66 (m, 14H), 1.77–1.91 (m, 4H), 1.99–2.02 (m, 1H), 2.16 (brs, 1H), 2.21–2.30 (m, 1H), 3.03 (d, *J* = 7.2 Hz, 1H), 3.41 (dt, *J* = 6.0, 18.6 Hz, 1H), 4.06–4.07 (m, 1H); ^13^C-NMR (100 MHz, CDCl_3_) δ 13.6, 17.4, 20.1, 22.4, 25.6 (t, *J* = 3.8 Hz), 27.4, 28.2, 30.6, 33.5, 39.8 (t, *J* = 21.9 Hz), 40.3, 41.9, 52.6, 56.9, 69.3, 72.3, 76.7 (t, *J* = 30.5 Hz), 126.7 (t, *J* = 246.0 Hz); HRMS (ESI^−^) calcd for C_18_H_32_O_3_F_2_Cl [M + Cl]^−^ 369.2014, found 369.1989.

*(3S,6R)-4,4-Difluoro-6-[(1R,3aR,4S,7aR)-4-hydroxy-7a-methyloctahydro-1H-inden-1-yl]-2-methylheptane-2,3-diol* (**11**)

To the solution of **30** (48.9 mg, 0.11 mmol) in MeOH (5 mL) and CH_2_Cl_2_ (5 mL), *p*-toluenesulfonic acid monohydrate (204.1 mg, 1.07 mmol) was added, and the mixture was stirred at room temperature for 66 h under air. After the reaction was quenched with H_2_O and saturated aqueous NaHCO_3_ at room temperature, the mixture was extracted with CH_2_Cl_2_ three times. The organic layer was dried over Na_2_SO_4_, filtered, and concentrated. The residue was purified by flash column chromatography on silica gel (hexane:EtOAc = 1:2) to obtain **11** (30.4 mg, 83%, in two steps) as a white powder.

**11**: ${\left[\alpha \right]}_{D}^{27}$ +22.6 (c 2.78, CHCl_3_); IR (neat) 3396, 1468, 1379, 1275, 1162, 1073, 987, 741 cm^−1^; ^1^H-NMR (600 MHz, CDCl_3_) δ 0.97 (s, 3H), 1.04 (d, *J* = 6.0 Hz, 3H), 1.08–1.20 (m, 2H), 1.28–1.91 (m, 18H), 2.00–2.19 (m, 3H), 2.93 (d, *J* = 7.8 Hz, 1H), 3.42 (ddd, *J* = 4.1, 7.8, 21.1 Hz, 1H), 4.06–4.08 (m, 1H); ^13^C-NMR (150 MHz, CDCl_3_) δ 13.3, 17.3, 21.0 (d, *J* = 3.0 Hz), 22.4, 25.5, 27.4, 28.2, 30.1 (d, *J* = 2.9 Hz), 33.5, 39.4 (t, *J* = 22.3 Hz), 40.3, 41.9, 52.7, 56.9, 69.3, 72.0, 77.5 (t, *J* = 25.0 Hz), 126.1 (t, *J* = 245.6 Hz); HRMS (ESI^−^) calcd for C_18_H_32_O_3_F_2_Cl [M + Cl]^−^ 369.2014, found 369.1983.

*(1R,3aR,7aR)-1-{(2R)-4,4-Difluoro-6-methyl-6[(triethylsilyl)oxy] heptan-2-yl}-7a-methyloctahydro-4H-inden-4-one* (**32**)

4-Methylmorpholine *N*-oxide (36.6 mg, 0.31 mmol) was added to the solution of **9** (63.8 mg, 0.20 mmol) in CH_2_Cl_2_ (2 mL), and the mixture was cooled to 0 °C. TPAP (37.8 mg, 0.11 mmol) was added to the mixture, and the mixture was stirred at 0 °C for 1 h. The reaction was diluted with an excess amount of Et_2_O. The mixture was directly purified by flash column chromatography on silica gel (Et_2_O only) to obtain the crude ketone (**31**), and this was used for the next reaction without further purification.

TESCl (211.0 mg, 234 μL, 1.4 mmol) was added to the 0 °C cooled solution of the above crude ketone (**31**) and imidazole (130.6 mg, 1.92 mmol) in CH_2_Cl_2_ (5 mL), and the mixture was stirred at room temperature for 28 h. After the reaction was quenched with H_2_O at 0 °C, the mixture was extracted with CH_2_Cl_2_ three times. The organic layer was dried over Na_2_SO_4_, filtered, and concentrated. The residue was purified by flash column chromatography on silica gel (hexane:EtOAc = 20:1–10:1) to obtain **32** (76.1 mg, 88%, in two steps) as a colorless oil.

**32**: ${\left[\alpha \right]}_{D}^{27}$ +6.4 (c 2.36, CHCl_3_); IR (neat) 1715, 1464, 1387, 1371, 1239, 1177, 1042, 748 cm^−1^; ^1^H-NMR (600 MHz, CDCl_3_) δ 0.58 (q, *J* = 7.8 Hz, 6H), 0.68 (s, 3H), 0.95 (t, *J* = 7.8 Hz, 9H), 1.10 (d, *J* = 6.0 Hz, 3H), 1.31–1.37 (m, 7H), 1.46 (q, *J* = 9.6 Hz, 1H), 1.50–1.78 (m, 4H), 1.82–2.06 (m, 7H), 2.12–2.16 (m, 1H), 2.19–2.30 (m, 2H), 2.46 (dd, *J* = 7.8, 12.0 Hz, 1H); ^13^C-NMR (150 MHz, CDCl_3_) δ 6.7, 7.0, 12.3, 19.1, 21.0, 23.9, 27.7, 30.7, 30.9, 38.9, 40.9, 43.1 (t, *J* = 23.0 Hz), 49.8, 51.2 (t, *J* = 23.7 Hz), 57.0, 62.0, 72.1, 125.3 (t, *J* = 240.6 Hz), 211.8; HRMS (ESI^+^) calcd for C_24_H_44_O_2_F_2_SiNa [M + Na]^+^ 453.2971 found 453.2953.

*(1R,3aR,4S,7aR)-1-{(2R)-4,4-Difluoro-4-[(4R)-2-(4-methoxyphenyl)-5,5-dimethyl-1,3-dioxolan-4-yl]butan-2-yl}-7a-methyloctahydro-1H-inden-4-ol* (**33**)

Pyridinium *p*-toluenesulfonate (PPTS) (19.3 mg, 0.08 mmol) was added to a solution of **10** (45.7 mg, 0.14 mmol) in anisaldehyde dimethyl acetal (1.5 mL) at room temperature, and the mixture was stirred at the same temperature for 18 h. After the reaction was quenched with water and saturated aqueous NaHCO_3_, the mixture was extracted with EtOAc three times. The organic layer was dried over Na_2_SO_4_, filtered, and concentrated. The residue was partially purified by flash column chromatography on silica gel (hexane:EtOAc = 4:1–2:1), and repurification by flash column chromatography on silica gel (hexane:EtOAc = 3:1) provided **33** (mixture of diastereomers) (31.3 mg, 50%) as a colorless oil.

**33**: IR (neat) 3481, 1523, 1460, 1379, 1252, 1170, 1096, 991, 829 cm^−1^; ^1^H-NMR (600 MHz, CDCl_3_) δ 0.96–0.96 (m, 3H), 1.06–1.18 (m, 5H), 1.25–1.35 (m, 3H), 1.41–1.62 (m, 12H), 1.79–1.93 (m, 4H), 1.99–2.20 (m, 1H), 2.17–2.28 (m, 1H), 3.79–3.83 (m, 4H), 4.06 (brs, 1H), 5.87 (s, 0.57H), 6.03 (s, 0.45H), 6.89–6.91 (m, 2H), 7.38–7.44 (m, 2H); ^13^C-NMR (150 MHz, CDCl_3_) δ 13.3, 17.4, 20.7, 20.8, 21.4, 21.4, 22.4, 23.4, 27.1, 27.3, 27.4, 28.0, 30.1, 30.2, 30.3, 30.3, 33.5, 40.3, 40.3, 40.5 (t, *J* = 23.0 Hz), 41.3 (t, *J* = 22.3 Hz), 41.9, 41.9, 52.7, 55.3, 56.8, 56.9, 69.3, 80.9, 81.6, 83.3, 83.5, 83.6, 83.6, 83.7, 83.8, 83.9, 84.0, 101.8, 102.1, 113.7, 113.7, 123.5 (dd, *J* = 241.2, 251.3 Hz), 123.7 (dd, *J* = 241.4, 251.4 Hz), 127.9, 128.4, 129.3, 130.5, 160.4, 160.5; HRMS (ESI^+^) calcd for C_26_H_38_O_4_F_2_Na [M + Na]^+^ 475.2630, found 475.2636.

*(1R,3aR,4S,7aR)-1-{(2R)-4,4-Difluoro-4-[(4S)-2-(4-methoxyphenyl)-5,5-dimethyl-1,3-dioxolan-4-yl]butan-2-yl}-7a-methyloctahydro-1H-inden-4-ol* (**34**)

Pyridinium *p*-toluenesulfonate (PPTS) (158.6 mg, 0.63 mmol) was added to a solution of **11** (36.1 mg, 0.11 mmol) in anisaldehyde dimethyl acetal (3 mL) at room temperature, and the mixture was stirred at the same temperature for 18 h. After the reaction was quenched with water and saturated aqueous NaHCO_3_, the mixture was extracted with EtOAc three times. The organic layer was dried over Na_2_SO_4_, filtered, and concentrated. The residue was partially purified by flash column chromatography on silica gel (hexane only–hexane:EtOAc = 4:1–2:1), and repurification by flash column chromatography on silica gel (hexane:EtOAc = 4:1) provided **34** (mixture of diastereomers) (22.9 mg, 61%) as a colorless oil.

**34**: IR (neat) 3516, 1519, 1460, 1375, 1252, 1166, 1093, 987, 829 cm^−1^; ^1^H-NMR (600 MHz, CDCl_3_) δ 0.96 (s, 3H), 1.05–1.35 (m, 8H), 1.40–1.88 (m, 15H), 1.96–2.07 (m, 2H), 3.81–3.84 (m, 4H), 4.05–4.07 (m, 1H), 5.55 (s, 0.56H), 6.04 (s, 0.44H), 6.90 (d, *J* = 7.8 Hz, 2H), 7.39–7.45 (m, 2H); ^13^C-NMR (150 MHz, CDCl_3_) δ 13.3, 13.3, 17.3, 21.5, 21.5, 22.4, 23.3 (t, *J* = 4.4 Hz), 27.1, 27.3, 27.4, 28.1, 30.6, 33.5, 40.3, 40.4 (t, *J* = 21.5 Hz), 41.2 (t, *J* = 23.0 Hz), 41.9, 52.6, 55.3, 56.8, 56.8, 69.3, 80.9, 81.6, 82.8, 82.9, 83.0, 83.0, 83.2, 83.2, 83.3, 83.5, 101.9, 102.2, 113.7, 113.8, 123.7 (dd, *J* = 239.9, 251.4 Hz), 123.9 (dd, *J* = 241.2, 251.3 Hz), 127.8, 128.4, 129.4, 130.5, 160.4, 160.5; HRMS (ESI^+^) calcd for C_26_H_38_O_4_F_2_Na [M + Na]^+^ 475.2630, found 475.2604.

*(1R,3aR,7aR)-1-{(2R)-4,4-Difluoro-4-[(4R)-2-(4-methoxyphenyl)-5,5-dimethyl-1,3-dioxolan-4-yl]butan-2-yl}-7a-methyloctahydro-4H-inden-4-one* (**35**)

4-Methylmorpholine *N*-oxide (14.4 mg, 0.12 mmol) was added to the solution of **33** (29.9 mg, 0.07 mmol) in CH_2_Cl_2_ (3 mL), and the mixture was cooled to 0 °C. TPAP (14.2 mg, 0.04 mmol) was added to the mixture, and the mixture was stirred at 0 °C for 40 min. The reaction was diluted with an excess amount of Et_2_O. The mixture was directly purified by flash column chromatography on silica gel (Et_2_O only) followed by purification on flash column chromatography on silica gel (hexane:EtOAc = 4:1) to obtain **35** (mixture of diastereomers) (26.5 mg, 89%) as a colorless oil.

**35**: IR (neat) 1712, 1615, 1519, 1468, 1387, 1252, 1170, 1096, 1031, 833, 736 cm^−1^; ^1^H-NMR (600 MHz, CDCl_3_) δ 0.65–0.67 (m, 3H), 1.12 (t, *J* = 6.9 Hz, 3H), 1.22–1.38 (m, 1H), 1.46–1.77 (m, 11H), 1.82–1.94 (m, 3H), 1.98–2.03 (m, 1H), 2.10–2.30 (m, 4H), 2.42–2.46 (m, 1H), 3.78–3.82 (m, 4H), 5.87 (s, 0.55H), 6.03 (s, 0.44H), 6.88–6.91 (m, 2H), 7.38–7.44 (m, 2H); ^13^C-NMR (150 MHz, CDCl_3_) δ 12.3, 12.3, 19.0, 20.9, 21.0, 21.4, 21.4, 23.3, 23.9, 27.1, 27.5, 27.6, 28.0, 30.2, 30.2, 30.4, 30.4, 38.8, 38.9, 40.4 (t, *J* = 22.3 Hz), 40.9, 41.3 (t, *J* = 23.0 Hz), 49.8, 55.3, 56.8, 56.8, 62.0, 80.9, 81.6, 83.3, 83.4, 83.5, 83.7, 83.8, 84.0, 101.9, 102.2, 113.7, 113.8, 121.8, 121.9, 123.4, 123.5, 123.6, 125.0, 125.2, 127.9, 128.4, 129.2, 130.3, 160.4, 160.6, 211.7; HRMS (ESI^+^) calcd for C_26_H_36_O_4_F_2_Na [M + Na]^+^ 473.2474, found 473.2492.

*(1R,3aR,7aR)-1-{(2R)-4,4-Difluoro-4-[(4S)-2-(4-methoxyphenyl)-5,5-dimethyl-1,3-dioxolan-4-yl]butan-2-yl}-7a-methyloctahydro-4H-inden-4-one* (**36**)

4-Methylmorpholine *N*-oxide (13.0 mg, 0.11 mmol) was added to the solution of **34** (31.3 mg, 0.07 mmol) in CH_2_Cl_2_ (3 mL), and the mixture was cooled to 0 °C. TPAP (13.1 mg, 0.04 mmol) was added to the mixture, and the mixture was stirred at 0 °C for 35 min. The reaction was diluted with an excess amount of Et_2_O. The mixture was directly purified by flash column chromatography on silica gel (Et_2_O only) followed by purification on flash column chromatography on silica gel (hexane:EtOAc = 4:1) to obtain **36** (mixture of diastereomers) (28.2 mg, 90%) as a colorless oil.

**36**: IR (neat) 1712, 1611, 1519, 1464, 1383, 1248, 1174, 1096, 1035, 837 cm^−1^; ^1^H-NMR (600 MHz, CDCl_3_) δ 0.66 (s, 3H), 1.10–1.12 (m, 3H), 1.24–1.36 (m, 1H), 1.40–1.61 (m, 10H), 1.66–2.04 (m, 7H), 2.11–2.14 (m, 1H), 2.18–2.24 (m, 1H), 2.27–2.30 (m, 1H), 2.41–2.46 (m, 1H), 3.79–3.83 (m, 4H), 5.88 (s, 0.53H), 6.04 (s, 0.44H), 6.89–6.91 (m, 2H), 7.38–7.44 (m, 2H); ^13^C-NMR (150 MHz, CDCl_3_) δ 12.4, 19.0, 20.5, 20.6, 21.4, 21.5, 23.2, 23.9, 27.1, 27.5, 27.6, 28.1, 30.7, 38.9, 40.4 (t, *J* = 22.3 Hz), 41.0, 41.1 (t, *J* = 23.0 Hz), 49.8, 55.3, 55.3, 56.7, 56.8, 62.0, 80.4, 81.6, 82.8, 82.9, 83.0, 83.0, 83.2, 83.2, 83.3, 83.5, 101.9, 102.2, 113.7, 113.8, 122.0, 122.1, 123.6, 123.6, 123.7, 123.8, 125.2, 125.4, 127.8, 128.4, 129.3, 130.4, 160.4, 160.6, 211.7, 211.7; HRMS (ESI^+^) calcd for C_26_H_36_O_4_F_2_Na [M + Na]^+^ 473.2474, found 473.2499.

*(3R,5Z,7E)-23,23-Difluoro-9,10-seco-5,7,10(19)-cholestatriene-3,25-diol: 23,23-Difluoro-25-hydroxyvitamin D_3_* (**5**)

*n*BuLi (183 μL, 1.55 M hexane solution, 0.29 mmol) was added to a solution of A-ring phosphine oxide **14** (129.1 mg, 0.29 mmol) in THF (1 mL) at −78 °C. After stirring for 20 min, a solution of **32** (30.7 mg, 0.07 mmol) in THF (1.5 mL) was added to the reaction mixture, and the mixture was stirred at −78 °C for 1 h. After the reaction was quenched with H_2_O at the same temperature, the mixture was extracted with EtOAc three times. The organic layer was dried over Na_2_SO_4_, filtered, and concentrated. The residue was purified by flash column chromatography on silica gel (hexane:EtOAc = 20:1) to obtain the crude coupling product (52.0 mg), and it was used for the next reaction without further purification. 

Tetrabutylammonium fluoride (710 μL, 1 M THF solution, 0.71 mmol) was added to the solution of the above crude coupling product (52.0 mg) in THF (4 mL), and the mixture was stirred at room temperature for 16 h. After the reaction was quenched with H_2_O and aqueous saturated NH_4_Cl at room temperature, the mixture was extracted with EtOAc three times. The organic layer was dried over Na_2_SO_4_, filtered, and concentrated. The residue was purified by flash column chromatography on silica gel (hexane:EtOAc = 1:1) to afford **5** (30.8 mg, 100%, in two steps) as a white powder.

**5**: ${\left[\alpha \right]}_{D}^{27}$ +53.4 (c 2.37, EtOH); IR (neat) 3381, 1441, 1379, 1220, 1174, 1054, 895, 860, 741 cm^−1^; ^1^H-NMR (600 MHz, CD_3_OD) δ 0.63 (s, 3H), 1.12 (d, *J* = 7.2 Hz, 3H), 1.33–1.76 (m, 16H), 1.83–2.18 (m, 9H), 2.23 (dd, *J* = 9.6, 12.0 Hz, 1H), 2.44 (dt, *J* = 5.1, 13.2 Hz, 1H), 2.57 (dd, *J* = 3.9, 12.9 Hz, 1H), 2.88–2.91 (m, 1H), 3.78–3.82 (m, 1H), 4.78 (d, *J* = 1.2 Hz, 1H), 5.08 (brs, 1H), 6.08 (d, *J* = 10.8 Hz, 1H), 6.26 (d, *J* = 10.8 Hz, 1H); ^13^C-NMR (150 MHz, CD_3_OD) δ 12.6, 21.7, 23.5, 24.8, 29.2, 30.2, 30.6, 30.8, 33.1, 33.9, 36.9, 42.1, 45.2 (t, *J* = 23.0 Hz), 47.2, 47.3, 50.6 (t, *J* = 23.7 Hz), 57.9, 58.5, 70.4, 70.9, 113.0, 119.4, 122.9, 127.0 (t, *J* = 240.5 Hz), 137.8, 142.6, 147.3; ^19^F NMR (565 MHz, CD_3_OD) δ −94.3 (d, *J* = 224.7 Hz), −92.7 (d, *J* = 243.4 Hz); HRMS (ESI^+^) calcd for C_27_H_42_O_4_F_2_Na [M + Na]^+^ 459.3045, found 459.3033.

*(3R,24R,5Z,7E)-23,23-Difluoro-9,10-seco-5,7,10(19)-cholestatriene-3,24,25-triol: (24R)-23,23-Difluoro-24,25-dihydroxyvitamin D_3_* (**7**)

*n*BuLi (152 μL, 1.55 M hexane solution, 0.24 mmol) was added to a solution of A-ring phosphine oxide **14** (112.9 mg, 0.25 mmol) in THF (1 mL) at −78 °C. After stirring for 20 min, a solution of **35** (26.5 mg, 0.06 mmol) in THF (1 mL) was added to the reaction mixture, and the mixture was stirred at −78 °C for 2 h. After the reaction was quenched with H_2_O at the same temperature, the mixture was extracted with EtOAc three times. The organic layer was washed with brine, dried over Na_2_SO_4_, filtered, and concentrated. The residue was purified by flash column chromatography on silica gel (hexane:EtOAc = 5:1) to obtain the crude coupling product (25.5 mg), and it was used for the next reaction without further purification.

The above crude residue was dissolved in MeOH (5 mL), and AG 50W-X4 resin H^+^ form (183.1 mg) was added. The mixture was stirred for 3 h, insoluble materials were filtered off, and the solution was diluted with H_2_O and saturated aqueous NaHCO_3_. The mixture was extracted with EtOAc three times. The organic layer was washed with brine, dried over Na_2_SO_4_, filtered, and concentrated. The residue was purified by flash column chromatography (hexane:EtOAc = 1:1) to afford **7** (10.8 mg, 41%, two steps) as a white solid. Crystal of **7** was obtained by dissolving **7** in EtOH and allowing the solvent to slowly evaporate at room temperature (colorless needles).

**7**: ${\left[\alpha \right]}_{D}^{27}$ +90.0 (c 0.83, EtOH); IR (neat) 3388, 1433, 1375, 1158, 1058, 1000, 957, 895 cm^−1^; ^1^H-NMR (600 MHz, CD_3_OD) δ 0.63 (s, 3H), 1.13 (d, *J* = 7.2 Hz, 3H), 1.29–1.63 (m, 13H), 1.70–1.79 (m, 3H), 1.88–2.33 (m, 9H), 2.44 (dt, *J* = 5.4, 13.8 Hz, 1H), 2.57 (dd, *J* = 3.6, 12.6 Hz, 1H), 2.88–2.91 (m, 1H), 3.44 (dd, *J* = 9.0, 13.8 Hz, 1H), 3.78–3.82 (m, 1H), 4.78 (d, *J* = 1.2 Hz, 1H), 5.08 (brs, 1H), 6.07 (d, *J* = 11.4 Hz, 1H), 6.26 (d, *J* = 10.8 Hz, 1H); ^13^C-NMR (150 MHz, CD_3_OD) δ 12.5, 22.2, 23.5, 24.8, 27.0 (d, *J* = 4.4 Hz), 27.8, 29.2, 30.2, 32.4, 33.9, 36.9, 41.1 (t, *J* = 22.3 Hz), 42.1, 47.2, 47.3, 57.9, 58.7, 70.9, 73.0, 79.5 (t, *J* = 26.6 Hz), 113.0, 119.4, 122.9, 127.1 (t, *J* = 244.1 Hz), 137.7, 142.6, 147.3; ^19^F-NMR (565 MHz, CD_3_OD) δ −107.1 (d, *J* = 251.8 Hz), −101.8 (d, *J* = 251.2 Hz); HRMS (ESI^−^) calcd for C_27_H_42_O_3_F_2_Cl [M + Cl]^−^ 487.2796, found 487.2778.

### 4.1. Crystal Data of ***7*** (CCDC 2202393)

C_27_H_42_F_2_O_3_: *Mr* = 452.63, CuKα (λ = 1.54187 Å), orthorhombic, *P*bca, colorless block 0.200 × 0.150 × 0.040 mm, crystal dimensions *a* = 6.74568(15) Å, *b* = 10.5822(3) Å, *c* = 36.4192(7) Å, α = 90°, β = 90°, γ = 90°, *T* = 173 K, *Z* = 4, *V* = 32599.74(10) Å^3^, *D*calc = 1.156 g/cm^3^, μCuKα = 6.723 cm^−1^, *F*_000_ = 984.00, GOF = 1.162, *R*_int_ = 0.0686, *R*_1_ = 0.0614, w*R*_2_ = 0.1337.

All measurements were taken on a Rigaku Raxis Rapid imaging plate area detector with graphite monochromated Cu-Kα radiation. The data were collected at a temperature of –100 °C. The structure was solved by direct-method SIR97 and expanded using Fourier techniques. The non-hydrogen atoms were refined anisotropically. All calculations were performed using the Crystal Structure (Crystal Structure 4.2.2) crystallographic software package except for refinement, which was performed using SHELXL97.

### 4.2. Accession Codes of Compound ***7***

CCDC 2202393 contains the supplementary crystallographic data for this study. These data can be obtained free of charge via www.ccdc.cam.ac.uk/data_request/cif (accessed on 21 August 2022), by emailing data_request@ccdc.cam.ac.uk, or by contacting The Cambridge Crystallographic Data Centre, 12 Union Road, Cambridge CB2 1EZ, UK; Fax: +44-1223-336033.

*(3R,24S,5Z,7E)-23,23-Difluoro-9,10-seco-5,7,10(19)-cholestatriene-3,24,25-triol: (24S)-23,23-Difluoro-24,25-dihydroxyvitamin D_3_* (**8**)

*n*BuLi (161 μL, 1.55 M hexane solution, 0.25 mmol) was added to a solution of A-ring phosphine oxide **14** (115.4 mg, 0.25 mmol) in THF (1 mL) at −78 °C. After stirring for 20 min, a solution of **36** (28.2 mg, 0.06 mmol) in THF (1 mL) was added to the reaction mixture, and the mixture was stirred at −78 °C for 100 min. After the reaction was quenched with H_2_O at the same temperature, the mixture was extracted with EtOAc three times. The organic layer was washed with brine, dried over Na_2_SO_4_, filtered, and concentrated. The residue was purified by flash column chromatography on silica gel (hexane:EtOAc = 4:1) to obtain the crude coupling product (41.6 mg), and it was used for the next reaction without further purification.

The above crude residue was dissolved in MeOH (5 mL), and AG 50W-X4 resin H^+^ form (188.3 mg) was added. The mixture was stirred for 3 h 10 min, insoluble materials were filtered off, and the solution was diluted with H_2_O and saturated aqueous NaHCO_3_. The mixture was extracted with EtOAc three times. The organic layer was washed with brine, dried over Na_2_SO_4_, filtered, and concentrated. The residue was purified by flash column chromatography (hexane:EtOAc = 1:1) to afford **8** (19.6 mg, 63%, two steps) as a white powder.

**8**: ${\left[\alpha \right]}_{D}^{27}$ +12.0 (c 0.25, CHCl_3_); IR (neat) 3388, 1437, 1375, 1263, 1162, 1066, 996, 891, 741 cm^−1^; ^1^H-NMR (600 MHz, CD_3_OD) δ 0.64 (s, 3H), 1.12 (d, *J* = 7.2 Hz, 3H), 1.30–1.42 (m, 10H), 1.47–1.63 (m, 4H), 1.68–1.79 (m, 3H), 1.87–2.31 (m, 9H), 2.44 (dt, *J* = 5.1, 13.2 Hz, 1H), 2.57 (dd, *J* = 3.6, 12.6 Hz, 1H), 2.88–2.91 (m, 1H), 3.46 (dd, *J* = 7.2, 16.8 Hz, 1H), 3.78–3.82 (m, 1H), 4.79 (d, *J* = 2.4 Hz, 1H), 5.08 (brs, 1H), 6.08 (d, *J* = 10.8 Hz, 1H), 6.26 (d, *J* = 10.8 Hz, 1H); ^13^C-NMR (150 MHz, CD_3_OD) δ 12.6, 21.6, 23.6, 24.8, 26.7 (d, *J* = 4.2 Hz), 27.9, 29.1, 30.2, 32.7, 33.9, 36.9, 41.3 (t, *J* = 22.2 Hz), 42.2, 47.2, 47.3, 57.9, 58.7, 70.9, 73.1, 79.0 (t, *J* = 27.3 Hz), 113.0, 119.4, 122.9, 127.3 (t, *J* = 244.9 Hz), 137.7, 142.6, 147.3; ^19^F-NMR (565 MHz, CD_3_OD) δ −105.0 (d, *J* = 251.5 Hz), −101.6 (d, *J* = 250.9 Hz); HRMS (ESI^−^) calcd for C_27_H_42_O_3_F_2_Cl [M + Cl]^−^ 487.2796, found 487.2794.

### 4.3. Metabolism of 25(OH)D_3_ ***(1)*** and ***5*** by Recombinant hCYP24A1

The metabolism of 25(OH)D_3_ and its analogue **5** by CYP24A1 was analyzed using the membrane fraction prepared from recombinant *Escherichia coli* cells expressing human CYP24A1, as described in our previous study [29]. Briefly, the reaction mixture containing 0.02 µM of human CYP24A1, 2.0 µM of adrenodoxin (ADX), 0.2 µM of NADPH-adrenodoxin reductase (ADR), 1 mM of EDTA, 1 mM of NADPH, and 5.0 µM of each substrate in 100 mM Tris-HCl (pH 7.4) was incubated at 37 °C for 5 or 15 min. The metabolites were extracted with four volumes of CHCl_3_-CH_3_OH (3:1) and analyzed by HPLC under the following conditions: column, CAPCELL PAK C18 UG120 (5 μm) (4.6 × 250 mm) (SHISEIDO, Tokyo, Japan); UV detection, 265 nm; flow-rate, 1.0 mL min^−1^; column temperature, 40 °C; mobile phase, CH_3_CN: a linear gradient of 20–100% CH_3_CN aqueous solution per 25 min and 100% CH_3_CN for 10 min.

### 4.4. Measurement of hVDR Binding Affinity of 25(OH)D_3_ ***(1)*** and ***5***

The binding affinity of each analogue for hVDR was evaluated using the in vitro system based on the split-luciferase technique described in our previous study [30]. Briefly, 50 μL of cell lysate prepared from recombinant *E. coli* expressing split-luciferase vitamin D biosensor protein was added to each well of a 96-well plate and left for 10 min at room temperature. Then, 50 μL of the Luciferin solution containing 20 mM of MgSO_4_, 2 mM of D-luciferin, and 4 mM of adenosine triphosphate in 25 mM Tris-HCl (pH 7.4) were injected into each well and incubated for 15 min at room temperature. The luminescence (photon counts) was measured using a luminometer (Infinite 200 Pro 96-microplate luminometer, Tecan). The relative hVDR binding affinity of each analogue was evaluated based on the concentration at which the luminescence showed 50% of the maximum value.

## Figures and Tables

**Table 1:** Hydroxylation activities of human CYP24A1 toward 25(OH)D_3_ (**1**) and 23,23-F_2_-25(OH)D_3_ (**5**).

Substrate	(nmol/min/nmol P450)
25(OH)D_3_	6.2 ± 1.5
23,23-F2-25(OH)D_3_ (5)	0.77 ± 0.15
24,24-F2-25(OH)D_3_ [23]	0.53 ± 0.12 [25]

Data were obtained at a substrate concentration of 5 μM. Each value is the mean ± SD of three separate experiments.

**Table 2:** Relative hVDR binding affinity of 23,23-F_2_-25(OH)D_3_ (**5**).

Compound	Relative hVDR Binding Affinity (%)
25(OH)D_3_ (1)	100
23,23-F2-25(OH)D_3_ (5)	8.2
24,24-F2-25(OH)D_3_ [23]	180 [25]

## Data Availability

The data presented in this study are available on request from the corresponding author.

## Supplementary Materials

The following are available online at https://www.mdpi.com/article/10.3390/molecules27165352/s1. ^1^H and ^13^C-NMR spectra of all new compounds: **5**, **7**–**11**, **13**, **20**, **23**, **24**, **26**, **28–30**, and **32**–**36**, as well as ^19^F-NMR spectra of compounds: **5**, **7**, and **8**.

## Sample Availability

Samples of the compounds are available from the authors.

## Abbreviations

DAST: *N*:*N*-diethylaminosulfur trifluoride; VDR: vitamin D receptor; TBS: *tert*-butyldimethylsilyl; TPAP: tetrapropylammonium perruthenate; NMO: *4*-methylmorpholine *N*-oxide; TBAF: tetra-*n*-butylammonium fluoride; TES: triethylsilyl; LDA: lithium diisopropylamide; DBU: 1,8-Diazabicyclo [5.4.0]undec-7-ene; PPTS: pyridinium *p*-toluenesulfonate.
