# Supplementary material for: The First Convergent Synthesis of 23,23-Difluoro-25-hydroxyvitamin D3 and Its 24-Hydroxy Derivatives: Preliminary Assessment of Biological Activities
Source: Molecules. 2022 Aug 22;27(16):5352. doi: 10.3390/molecules27165352 (PMC9415778; doi:10.3390/molecules27165352)
Supplement: Supplementary file 1 [file molecules-27-05352-s001.zip › molecules-1853414-supplementary.pdf]

Supplementary Materials

The First Convergent Synthesis of 23,23-Difluoro-25-hydroxyvitamin D3 and Its 24-Hydroxy Derivatives: Preliminary Assessment of Biological Activities

Sayuri Mototani [1], Fumihiro Kawagoe [1], Kaori Yasuda [2], Hiroki Mano [2], Toshiyuki Sakaki [2] and Atsushi Kittaka [1,*]

[1] Faculty of Pharmaceutical Sciences, Teikyo University, 2-11-1 Kaga, Itabashi, Tokyo 173-8605, Japan
[2] Faculty of Engineering, Toyama Prefectural University, Imizu, Toyama 939-0398, Japan
* Correspondence: akittaka@pharm.teikyo-u.ac.jp; Tel.: +81-3-3964-8109; Fax: +81-3-3964-8117

Contents

Title page S1

^{1}H and ^{13}C-NMR spectra of all new compounds **20**, **13**, **23**, **24**, **9**, **26**, **28–30**, **10**, **11**, **32–36**, **5**, **7**, and **8**.
S2-S20
^{19}F-NMR spectra of **5**, **7**, and **8**. S21-S22

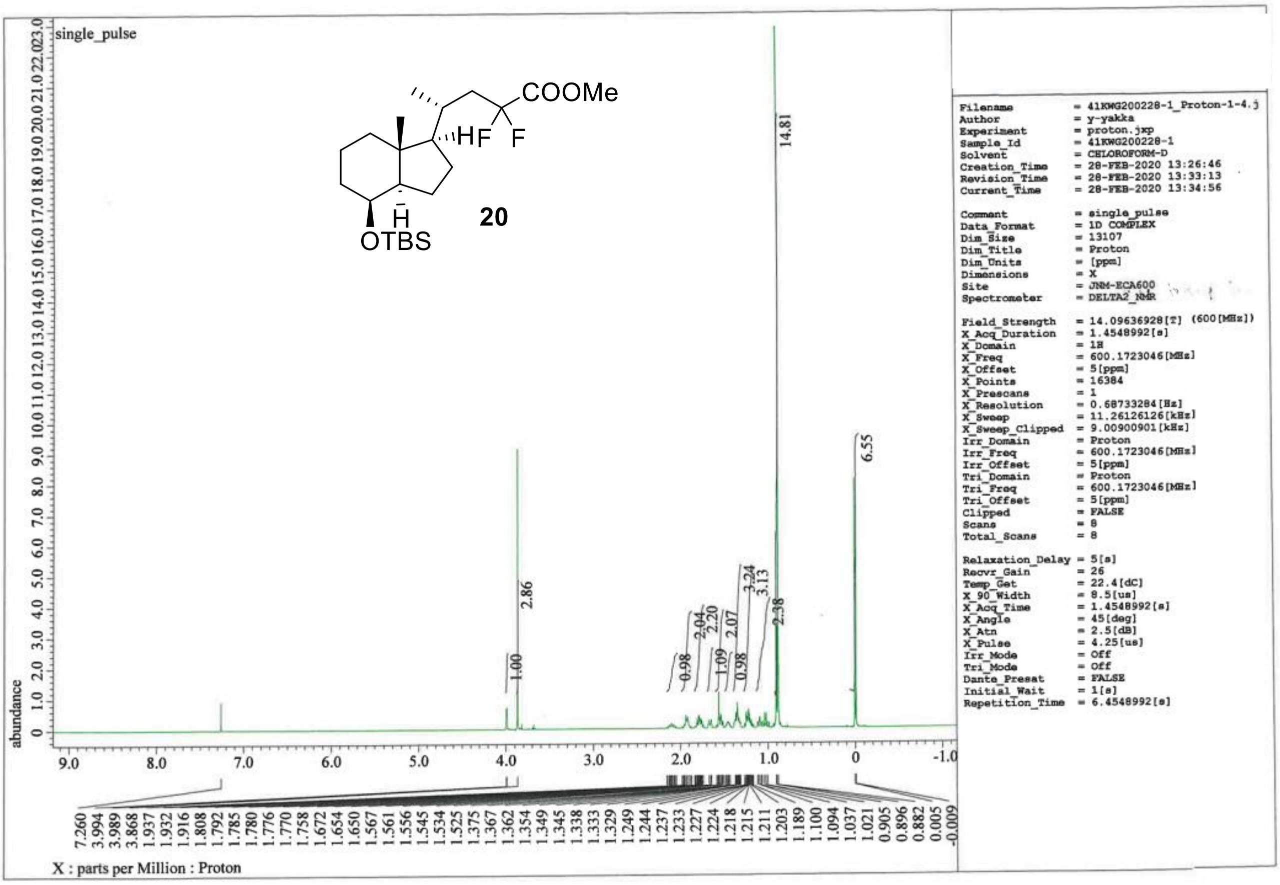
single_pulse
COOMe
H F F
H
OTBS
20
14.81
6.55
2.86
1.00
0.98
2.04
2.20
1.09
2.07
0.98
3.24
3.13
2.38
abundance
X : parts per Million : Proton
7.260 3.994 3.989 3.868 1.937 1.932 1.916 1.808 1.792 1.785 1.780 1.776 1.770 1.758 1.672 1.654 1.650 1.567 1.561 1.556 1.545 1.534 1.525 1.375 1.367 1.362 1.354 1.349 1.345 1.338 1.333 1.329 1.249 1.244 1.237 1.233 1.227 1.224 1.218 1.215 1.211 1.203 1.189 1.100 1.094 1.037 1.021 0.905 0.896 0.882 0.005 -0.009
Filename = 41KWG200228-1_Proton-1-4.j
Author = y-yakka
Experiment = proton.jxp
Sample_Id = 41KWG200228-1
Solvent = CHLOROFORM-D
Creation_Time = 28-FEB-2020 13:26:46
Revision_Time = 28-FEB-2020 13:33:13
Current_Time = 28-FEB-2020 13:34:56
Comment = single_pulse
Data_Format = 1D COMPLEX
Dim_Size = 13107
Dim_Title = Proton
Dim_Units = [ppm]
Dimensions = X
Site = JNM-ECA600
Spectrometer = DELTA2_NMR
Field_Strength = 14.09636928[T] (600[MHz])
X_Acq_Duration = 1.4548992[s]
X_Domain = 1H
X_Freq = 600.1723046[MHz]
X_Offset = 5[ppm]
X_Points = 16384
X_Prescans = 1
X_Resolution = 0.68733284[Hz]
X_Sweep = 11.26126126[kHz]
X_Sweep_Clipped = 9.00900901[kHz]
Irr_Domain = Proton
Irr_Freq = 600.1723046[MHz]
Irr_Offset = 5[ppm]
Tri_Domain = Proton
Tri_Freq = 600.1723046[MHz]
Tri_Offset = 5[ppm]
Clipped = FALSE
Scans = 8
Total_Scans = 8
Relaxation_Delay = 5[s]
Recvr_Gain = 26
Temp_Get = 22.4[dC]
X_90_Width = 8.5[us]
X_Acq_Time = 1.4548992[s]
X_Angle = 45[deg]
X_Atn = 2.5[dB]
X_Pulse = 4.25[us]
Irr_Mode = Off
Tri_Mode = Off
Dante_Presat = FALSE
Initial_Wait = 1[s]
Repetition_Time = 6.4548992[s]

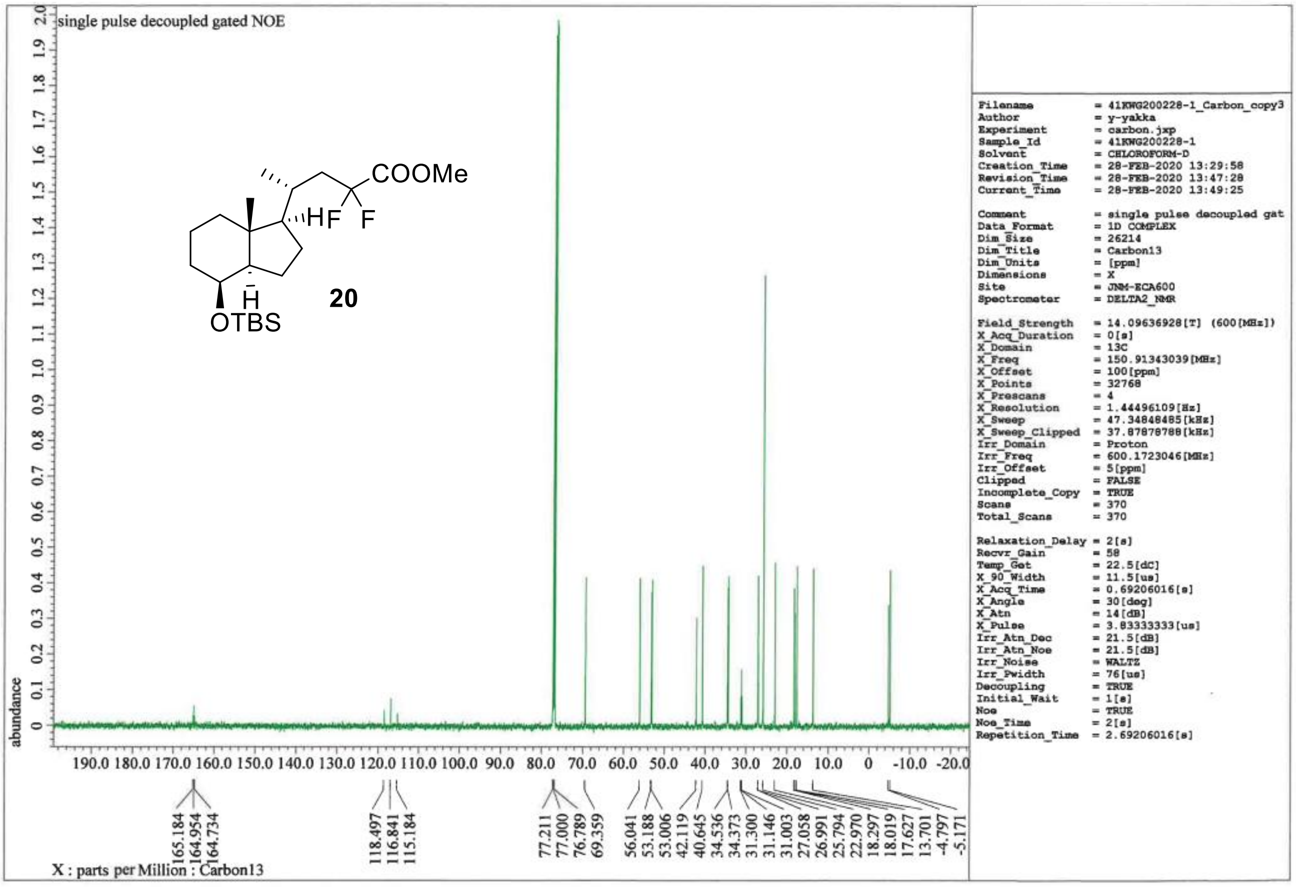
single pulse decoupled gated NOE
COOMe
H F F
H
OTBS
20
abundance
X : parts per Million : Carbon13
165.184 164.954 164.734 118.497 116.841 115.184 77.211 77.000 76.789 69.359 56.041 53.188 53.006 42.119 40.645 34.536 34.373 31.300 31.146 31.003 27.058 26.991 25.794 22.970 18.297 18.019 17.627 13.701 -4.797 -5.171
Filename = 41KWG200228-1_Carbon_copy3
Author = y-yakka
Experiment = carbon.jxp
Sample_Id = 41KWG200228-1
Solvent = CHLOROFORM-D
Creation_Time = 28-FEB-2020 13:29:58
Revision_Time = 28-FEB-2020 13:47:28
Current_Time = 28-FEB-2020 13:49:25
Comment = single pulse decoupled gat
Data_Format = 1D COMPLEX
Dim_Size = 26214
Dim_Title = Carbon13
Dim_Units = [ppm]
Dimensions = X
Site = JNM-ECA600
Spectrometer = DELTA2_NMR
Field_Strength = 14.09636928[T] (600[MHz])
X_Acq_Duration = 0[s]
X_Domain = 13C
X_Freq = 150.91343039[MHz]
X_Offset = 100[ppm]
X_Points = 32768
X_Prescans = 4
X_Resolution = 1.44496109[Hz]
X_Sweep = 47.34848485[kHz]
X_Sweep_Clipped = 37.87878788[kHz]
Irr_Domain = Proton
Irr_Freq = 600.1723046[MHz]
Irr_Offset = 5[ppm]
Clipped = FALSE
Incomplete_Copy = TRUE
Scans = 370
Total_Scans = 370
Relaxation_Delay = 2[s]
Recvr_Gain = 58
Temp_Get = 22.5[dC]
X_90_Width = 11.5[us]
X_Acq_Time = 0.69206016[s]
X_Angle = 30[deg]
X_Atn = 14[dB]
X_Pulse = 3.83333333[us]
Irr_Atn_Dec = 21.5[dB]
Irr_Atn_Noe = 21.5[dB]
Irr_Noise = WALTZ
Irr_Pwidth = 76[us]
Decoupling = TRUE
Initial_Wait = 1[s]
Noe = TRUE
Noe_Time = 2[s]
Repetition_Time = 2.69206016[s]

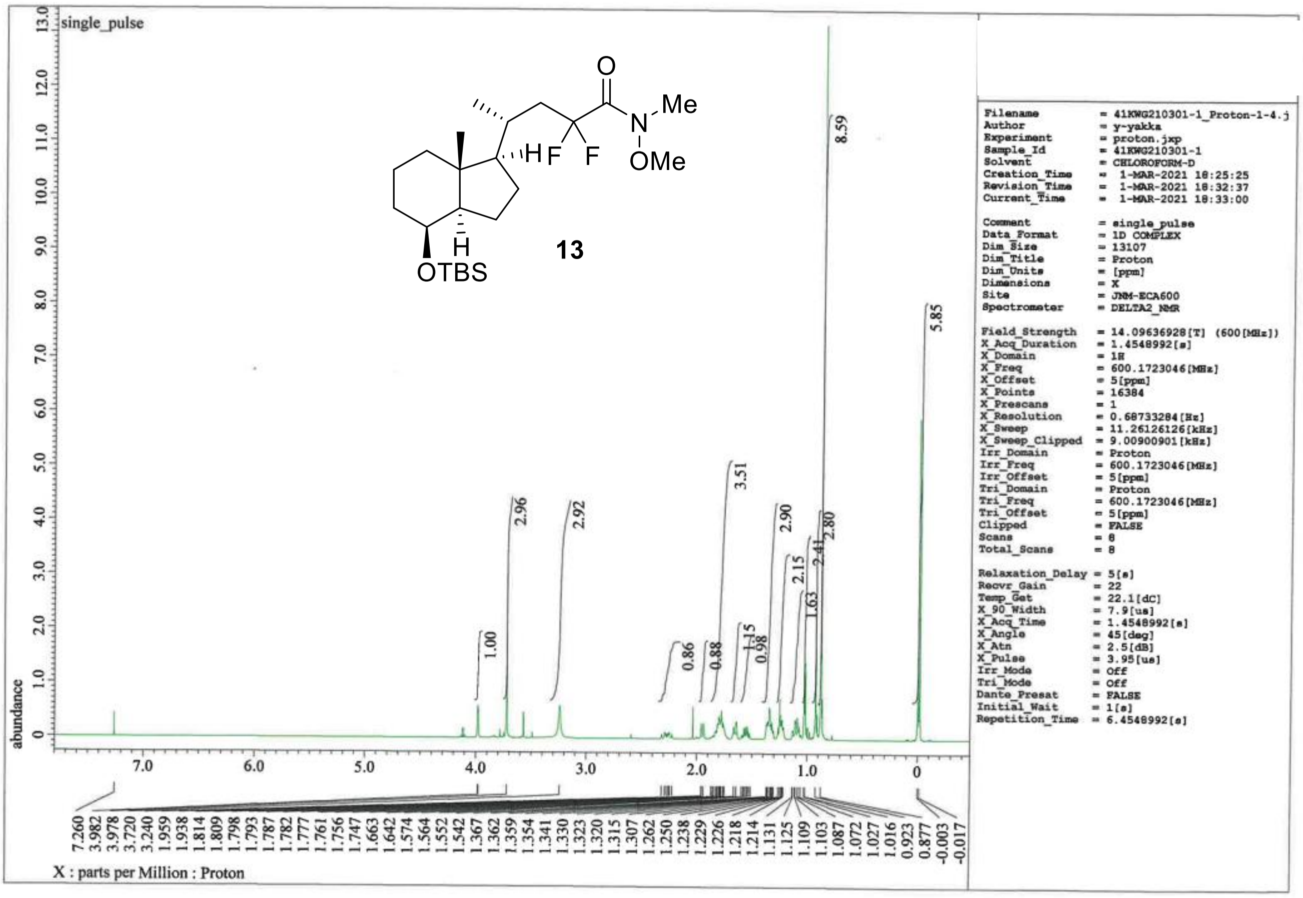
single_pulse
O
Me
N
HF F
OMe
H
OTBS
13
abundance
X : parts per Million : Proton
1.00
2.96
2.92
0.86
0.88
3.51
1.15
0.98
2.90
2.15
1.63
2.41
2.80
8.59
5.85
7.260
3.982
3.978
3.720
3.240
1.959
1.938
1.814
1.809
1.798
1.793
1.787
1.782
1.777
1.761
1.756
1.747
1.663
1.642
1.574
1.564
1.552
1.542
1.367
1.362
1.359
1.354
1.341
1.330
1.323
1.320
1.315
1.307
1.262
1.250
1.238
1.229
1.226
1.218
1.214
1.131
1.125
1.109
1.103
1.087
1.072
1.027
1.016
0.923
0.877
-0.003
-0.017
Filename = 41KWG210301-1_Proton-1-4.j
Author = y-yakka
Experiment = proton.jxp
Sample_Id = 41KWG210301-1
Solvent = CHLOROFORM-D
Creation_Time = 1-MAR-2021 18:25:25
Revision_Time = 1-MAR-2021 18:32:37
Current_Time = 1-MAR-2021 18:33:00
Comment = single_pulse
Data_Format = 1D COMPLEX
Dim_Size = 13107
Dim_Title = Proton
Dim_Units = [ppm]
Dimensions = X
Site = JNM-ECA600
Spectrometer = DELTA2_NMR
Field_Strength = 14.09636928[T] (600[MHz])
X_Acq_Duration = 1.4548992[s]
X_Domain = 1H
X_Freq = 600.1723046[MHz]
X_Offset = 5[ppm]
X_Points = 16384
X_Prescans = 1
X_Resolution = 0.68733284[Hz]
X_Sweep = 11.26126126[kHz]
X_Sweep_Clipped = 9.00900901[kHz]
Irr_Domain = Proton
Irr_Freq = 600.1723046[MHz]
Irr_Offset = 5[ppm]
Tri_Domain = Proton
Tri_Freq = 600.1723046[MHz]
Tri_Offset = 5[ppm]
Clipped = FALSE
Scans = 8
Total_Scans = 8
Relaxation_Delay = 5[s]
Recvr_Gain = 22
Temp_Get = 22.1[dC]
X_90_Width = 7.9[us]
X_Acq_Time = 1.4548992[s]
X_Angle = 45[deg]
X_Atn = 2.5[dB]
X_Pulse = 3.95[us]
Irr_Mode = Off
Tri_Mode = Off
Dante_Presat = FALSE
Initial_Wait = 1[s]
Repetition_Time = 6.4548992[s]

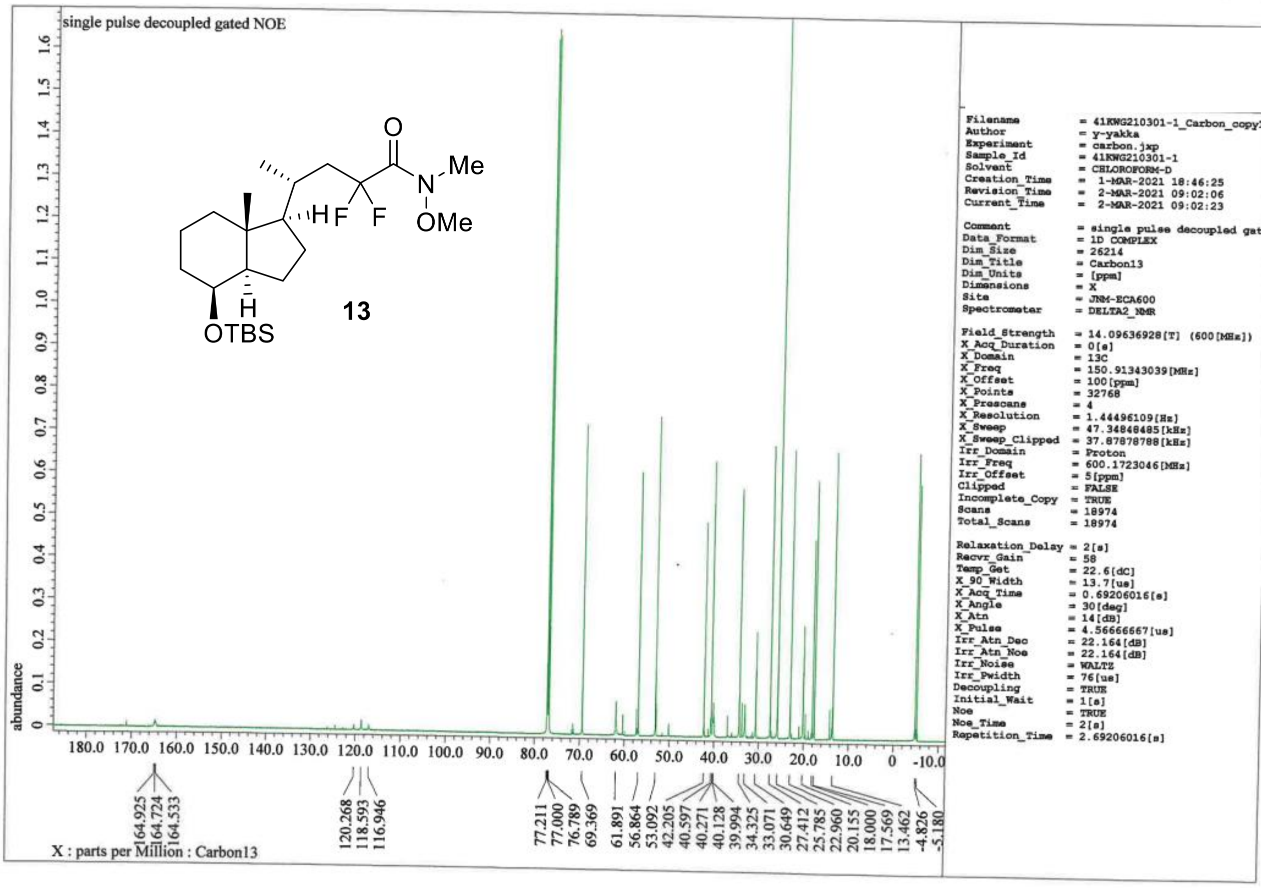
single pulse decoupled gated NOE
O
Me
N
HF F
OMe
H
OTBS
13
abundance
X : parts per Million : Carbon13
164.925
164.724
164.533
120.268
118.593
116.946
77.211
77.000
76.789
69.369
61.891
56.864
53.092
42.205
40.597
40.271
40.128
39.994
34.325
33.071
30.649
27.412
25.785
22.960
20.155
18.000
17.569
13.462
-4.826
-5.180
Filename = 41KWG210301-1_Carbon_copy1
Author = y-yakka
Experiment = carbon.jxp
Sample_Id = 41KWG210301-1
Solvent = CHLOROFORM-D
Creation_Time = 1-MAR-2021 18:46:25
Revision_Time = 2-MAR-2021 09:02:06
Current_Time = 2-MAR-2021 09:02:23
Comment = single pulse decoupled gat
Data_Format = 1D COMPLEX
Dim_Size = 26214
Dim_Title = Carbon13
Dim_Units = [ppm]
Dimensions = X
Site = JNM-ECA600
Spectrometer = DELTA2_NMR
Field_Strength = 14.09636928[T] (600[MHz])
X_Acq_Duration = 0[s]
X_Domain = 13C
X_Freq = 150.91343039[MHz]
X_Offset = 100[ppm]
X_Points = 32768
X_Prescans = 4
X_Resolution = 1.44496109[Hz]
X_Sweep = 47.34848485[kHz]
X_Sweep_Clipped = 37.87878788[kHz]
Irr_Domain = Proton
Irr_Freq = 600.1723046[MHz]
Irr_Offset = 5[ppm]
Clipped = FALSE
Incomplete_Copy = TRUE
Scans = 18974
Total_Scans = 18974
Relaxation_Delay = 2[s]
Recvr_Gain = 58
Temp_Get = 22.6[dC]
X_90_Width = 13.7[us]
X_Acq_Time = 0.69206016[s]
X_Angle = 30[deg]
X_Atn = 14[dB]
X_Pulse = 4.56666667[us]
Irr_Atn_Dec = 22.164[dB]
Irr_Atn_Noe = 22.164[dB]
Irr_Noise = WALTZ
Irr_Pwidth = 76[us]
Decoupling = TRUE
Initial_Wait = 1[s]
Noe = TRUE
Noe_Time = 2[s]
Repetition_Time = 2.69206016[s]

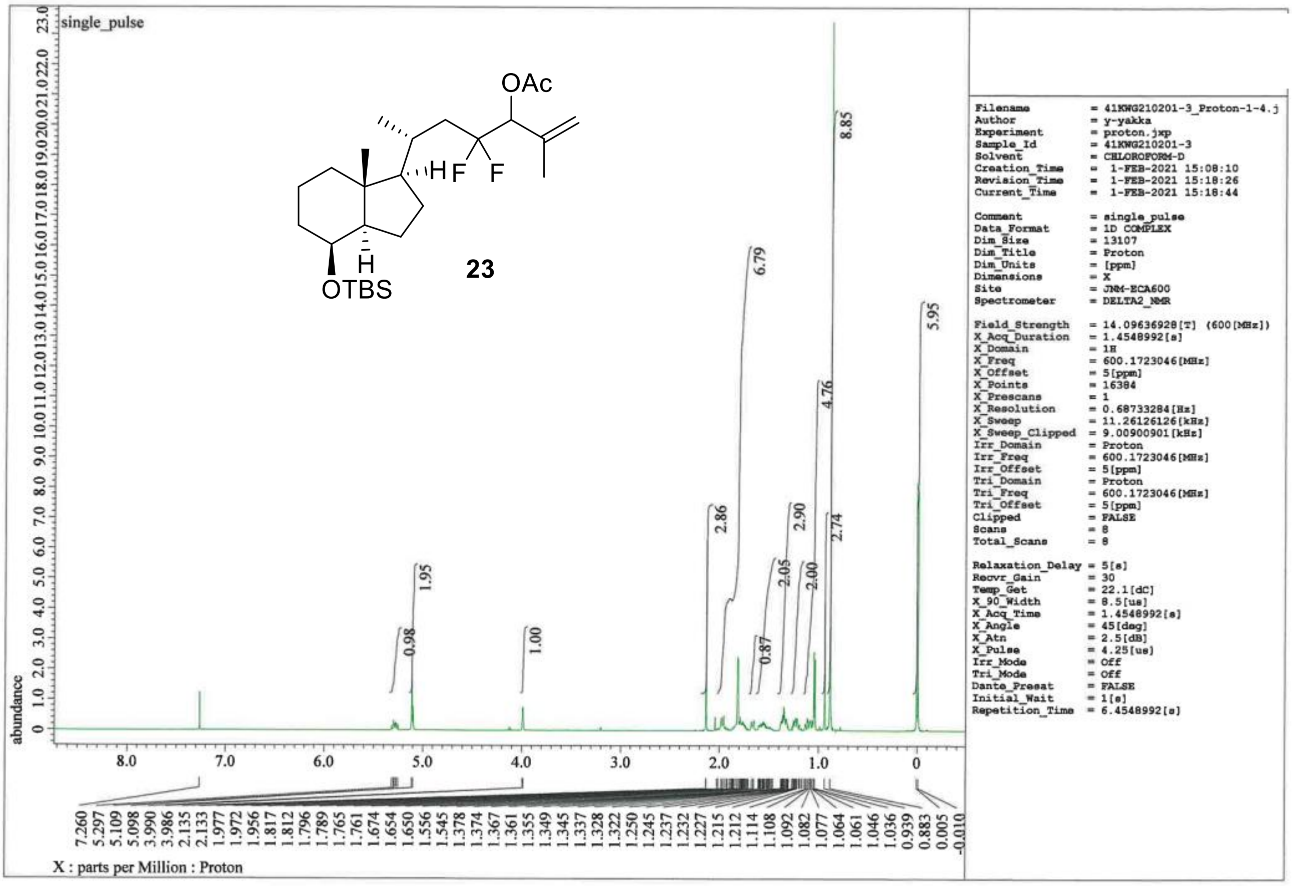
single_pulse
OAc
H F F
H
OTBS
23
8.85
6.79
5.95
4.76
2.86
2.90
2.74
2.05
2.00
1.95
0.98
1.00
0.87
abundance
X : parts per Million : Proton
7.260 5.297 5.109 5.098 3.990 3.986 2.135 2.133 1.977 1.972 1.956 1.817 1.812 1.796 1.789 1.765 1.761 1.674 1.654 1.650 1.556 1.545 1.378 1.374 1.367 1.361 1.355 1.349 1.345 1.337 1.328 1.322 1.250 1.245 1.237 1.232 1.227 1.215 1.212 1.114 1.108 1.092 1.082 1.077 1.064 1.061 1.046 1.036 0.939 0.883 0.005 -0.010
Filename = 41KWG210201-3_Proton-1-4.j
Author = y-yakka
Experiment = proton.jxp
Sample_Id = 41KWG210201-3
Solvent = CHLOROFORM-D
Creation_Time = 1-FEB-2021 15:08:10
Revision_Time = 1-FEB-2021 15:18:26
Current_Time = 1-FEB-2021 15:18:44
Comment = single_pulse
Data_Format = 1D COMPLEX
Dim_Size = 13107
Dim_Title = Proton
Dim_Units = [ppm]
Dimensions = X
Site = JNM-ECA600
Spectrometer = DELTA2_NMR
Field_Strength = 14.09636928[T] (600[MHz])
X_Acq_Duration = 1.4548992[s]
X_Domain = 1H
X_Freq = 600.1723046[MHz]
X_Offset = 5[ppm]
X_Points = 16384
X_Prescans = 1
X_Resolution = 0.68733284[Hz]
X_Sweep = 11.26126126[kHz]
X_Sweep_Clipped = 9.00900901[kHz]
Irr_Domain = Proton
Irr_Freq = 600.1723046[MHz]
Irr_Offset = 5[ppm]
Tri_Domain = Proton
Tri_Freq = 600.1723046[MHz]
Tri_Offset = 5[ppm]
Clipped = FALSE
Scans = 8
Total_Scans = 8
Relaxation_Delay = 5[s]
Recvr_Gain = 30
Temp_Get = 22.1[dC]
X_90_Width = 8.5[us]
X_Acq_Time = 1.4548992[s]
X_Angle = 45[deg]
X_Atn = 2.5[dB]
X_Pulse = 4.25[us]
Irr_Mode = Off
Tri_Mode = Off
Dante_Presat = FALSE
Initial_Wait = 1[s]
Repetition_Time = 6.4548992[s]

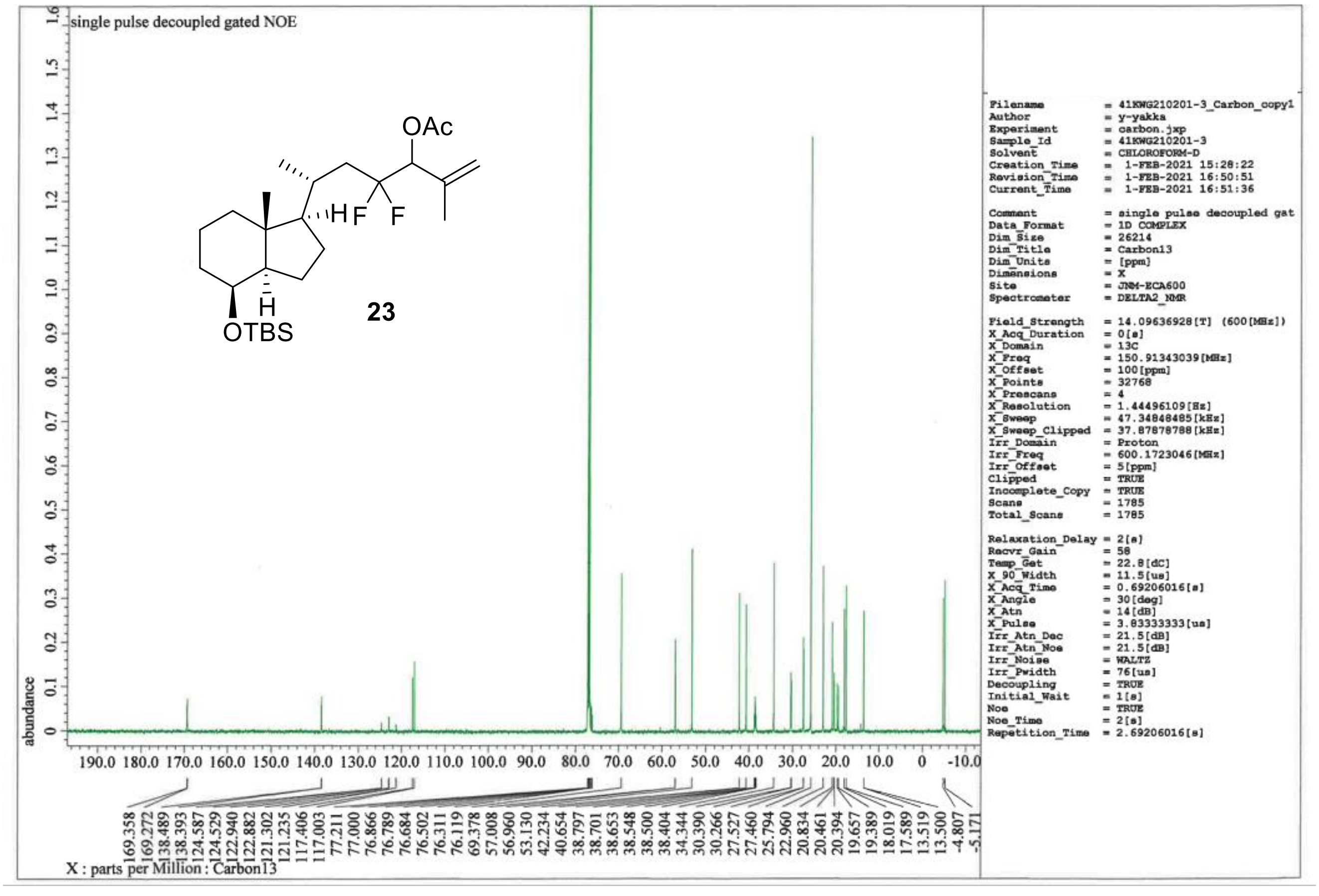
single pulse decoupled gated NOE
OAc
H F F
H
OTBS
23
abundance
X : parts per Million : Carbon13
169.358 169.272 138.489 138.393 124.587 124.529 122.940 122.882 121.302 121.235 117.406 117.003 77.211 77.000 76.866 76.789 76.684 76.502 76.311 76.119 69.378 57.008 56.960 53.130 42.234 40.654 38.797 38.701 38.653 38.548 38.500 38.404 34.344 30.390 30.266 27.527 27.460 25.794 22.960 20.834 20.461 20.394 19.657 19.389 18.019 17.589 13.519 13.500 -4.807 -5.171
Filename = 41KWG210201-3_Carbon_copy1
Author = y-yakka
Experiment = carbon.jxp
Sample_Id = 41KWG210201-3
Solvent = CHLOROFORM-D
Creation_Time = 1-FEB-2021 15:28:22
Revision_Time = 1-FEB-2021 16:50:51
Current_Time = 1-FEB-2021 16:51:36
Comment = single pulse decoupled gat
Data_Format = 1D COMPLEX
Dim_Size = 26214
Dim_Title = Carbon13
Dim_Units = [ppm]
Dimensions = X
Site = JNM-ECA600
Spectrometer = DELTA2_NMR
Field_Strength = 14.09636928[T] (600[MHz])
X_Acq_Duration = 0[s]
X_Domain = 13C
X_Freq = 150.91343039[MHz]
X_Offset = 100[ppm]
X_Points = 32768
X_Prescans = 4
X_Resolution = 1.44496109[Hz]
X_Sweep = 47.34848485[kHz]
X_Sweep_Clipped = 37.87878788[kHz]
Irr_Domain = Proton
Irr_Freq = 600.1723046[MHz]
Irr_Offset = 5[ppm]
Clipped = TRUE
Incomplete_Copy = TRUE
Scans = 1785
Total_Scans = 1785
Relaxation_Delay = 2[s]
Recvr_Gain = 58
Temp_Get = 22.8[dC]
X_90_Width = 11.5[us]
X_Acq_Time = 0.69206016[s]
X_Angle = 30[deg]
X_Atn = 14[dB]
X_Pulse = 3.83333333[us]
Irr_Atn_Dec = 21.5[dB]
Irr_Atn_Noe = 21.5[dB]
Irr_Noise = WALTZ
Irr_Pwidth = 76[us]
Decoupling = TRUE
Initial_Wait = 1[s]
Noe = TRUE
Noe_Time = 2[s]
Repetition_Time = 2.69206016[s]

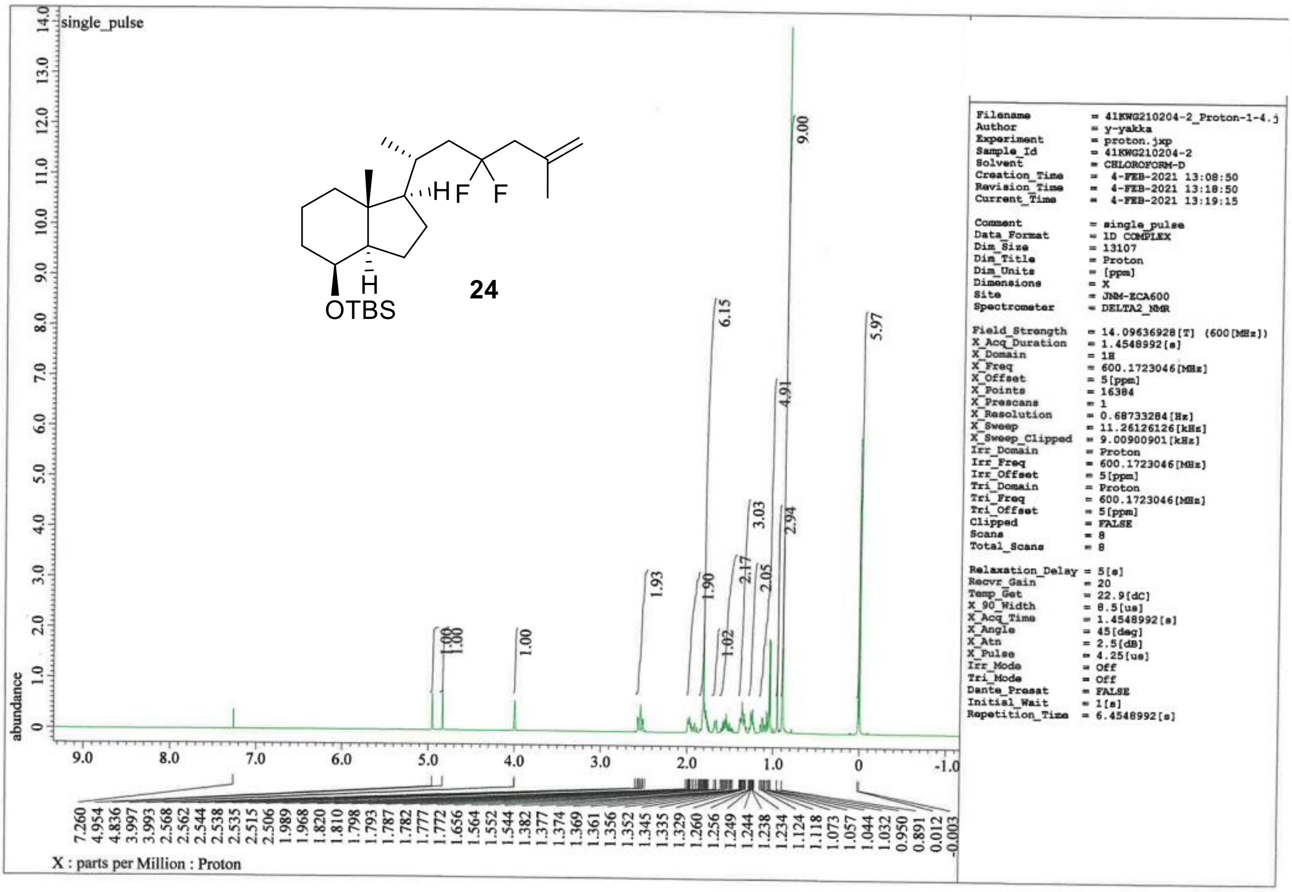
single_pulse
24
OTBS
abundance
X : parts per Million : Proton
Filename = 41KWG210204-2_Proton-1-4.j
Author = y-yakka
Experiment = proton.jxp
Sample_Id = 41KWG210204-2
Solvent = CHLOROFORM-D
Creation_Time = 4-FEB-2021 13:08:50
Revision_Time = 4-FEB-2021 13:18:50
Current_Time = 4-FEB-2021 13:19:15
Comment = single_pulse
Data_Format = 1D COMPLEX
Dim_Size = 13107
Dim_Title = Proton
Dim_Units = [ppm]
Dimensions = X
Site = JNM-ECA600
Spectrometer = DELTA2_NMR
Field_Strength = 14.09636928[T] (600[MHz])
X_Acq_Duration = 1.4548992[s]
X_Domain = 1H
X_Freq = 600.1723046[MHz]
X_Offset = 5[ppm]
X_Points = 16384
X_Prescans = 1
X_Resolution = 0.68733284[Hz]
X_Sweep = 11.26126126[kHz]
X_Sweep_Clipped = 9.00900901[kHz]
Irr_Domain = Proton
Irr_Freq = 600.1723046[MHz]
Irr_Offset = 5[ppm]
Tri_Domain = Proton
Tri_Freq = 600.1723046[MHz]
Tri_Offset = 5[ppm]
Clipped = FALSE
Scans = 8
Total_Scans = 8
Relaxation_Delay = 5[s]
Recvr_Gain = 20
Temp_Get = 22.9[dC]
X_90_Width = 8.5[us]
X_Acq_Time = 1.4548992[s]
X_Angle = 45[deg]
X_Atn = 2.5[dB]
X_Pulse = 4.25[us]
Irr_Mode = Off
Tri_Mode = Off
Dante_Presat = FALSE
Initial_Wait = 1[s]
Repetition_Time = 6.4548992[s]
1.00 1.00 1.00 1.93 1.90 1.02 6.15 2.17 2.05 3.03 4.91 2.94 9.00 5.97
7.260 4.954 4.836 3.997 3.993 2.568 2.562 2.544 2.538 2.535 2.515 2.506 1.989 1.968 1.820 1.810 1.798 1.793 1.787 1.782 1.777 1.772 1.656 1.564 1.552 1.544 1.382 1.377 1.374 1.369 1.361 1.356 1.352 1.345 1.335 1.329 1.260 1.256 1.249 1.244 1.238 1.234 1.124 1.118 1.073 1.057 1.044 1.032 0.950 0.891 0.012 -0.003

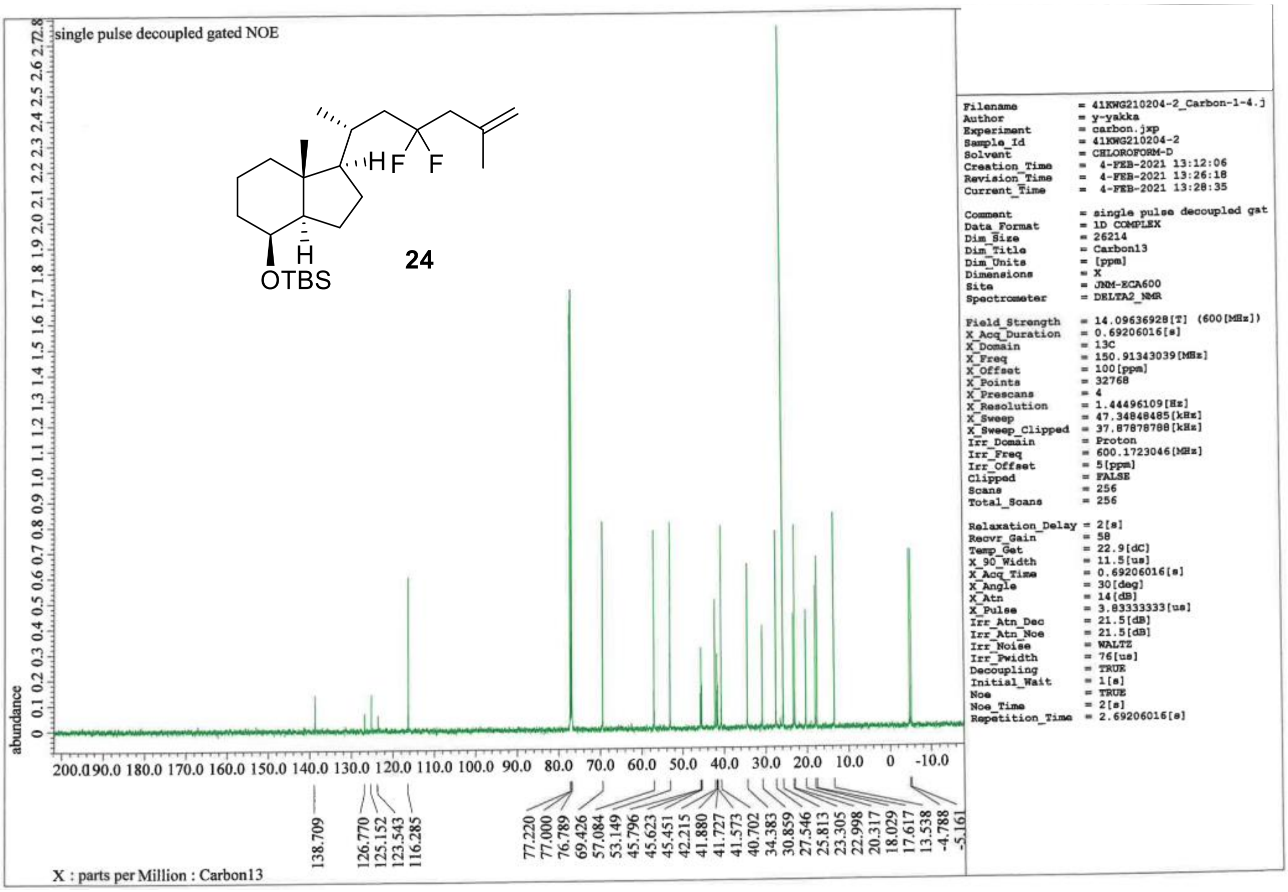
single pulse decoupled gated NOE
24
OTBS
abundance
X : parts per Million : Carbon13
Filename = 41KWG210204-2_Carbon-1-4.j
Author = y-yakka
Experiment = carbon.jxp
Sample_Id = 41KWG210204-2
Solvent = CHLOROFORM-D
Creation_Time = 4-FEB-2021 13:12:06
Revision_Time = 4-FEB-2021 13:26:18
Current_Time = 4-FEB-2021 13:28:35
Comment = single pulse decoupled gat
Data_Format = 1D COMPLEX
Dim_Size = 26214
Dim_Title = Carbon13
Dim_Units = [ppm]
Dimensions = X
Site = JNM-ECA600
Spectrometer = DELTA2_NMR
Field_Strength = 14.09636928[T] (600[MHz])
X_Acq_Duration = 0.69206016[s]
X_Domain = 13C
X_Freq = 150.91343039[MHz]
X_Offset = 100[ppm]
X_Points = 32768
X_Prescans = 4
X_Resolution = 1.44496109[Hz]
X_Sweep = 47.34848485[kHz]
X_Sweep_Clipped = 37.87878788[kHz]
Irr_Domain = Proton
Irr_Freq = 600.1723046[MHz]
Irr_Offset = 5[ppm]
Clipped = FALSE
Scans = 256
Total_Scans = 256
Relaxation_Delay = 2[s]
Recvr_Gain = 58
Temp_Get = 22.9[dC]
X_90_Width = 11.5[us]
X_Acq_Time = 0.69206016[s]
X_Angle = 30[deg]
X_Atn = 14[dB]
X_Pulse = 3.83333333[us]
Irr_Atn_Dec = 21.5[dB]
Irr_Atn_Noe = 21.5[dB]
Irr_Noise = WALTZ
Irr_Pwidth = 76[us]
Decoupling = TRUE
Initial_Wait = 1[s]
Noe = TRUE
Noe_Time = 2[s]
Repetition_Time = 2.69206016[s]
138.709 126.770 125.152 123.543 116.285 77.220 77.000 76.789 69.426 57.084 53.149 45.796 45.623 45.451 42.215 41.880 41.727 41.573 40.702 34.383 30.859 27.546 25.813 23.305 22.998 20.317 18.029 17.617 13.538 -4.788 -5.161

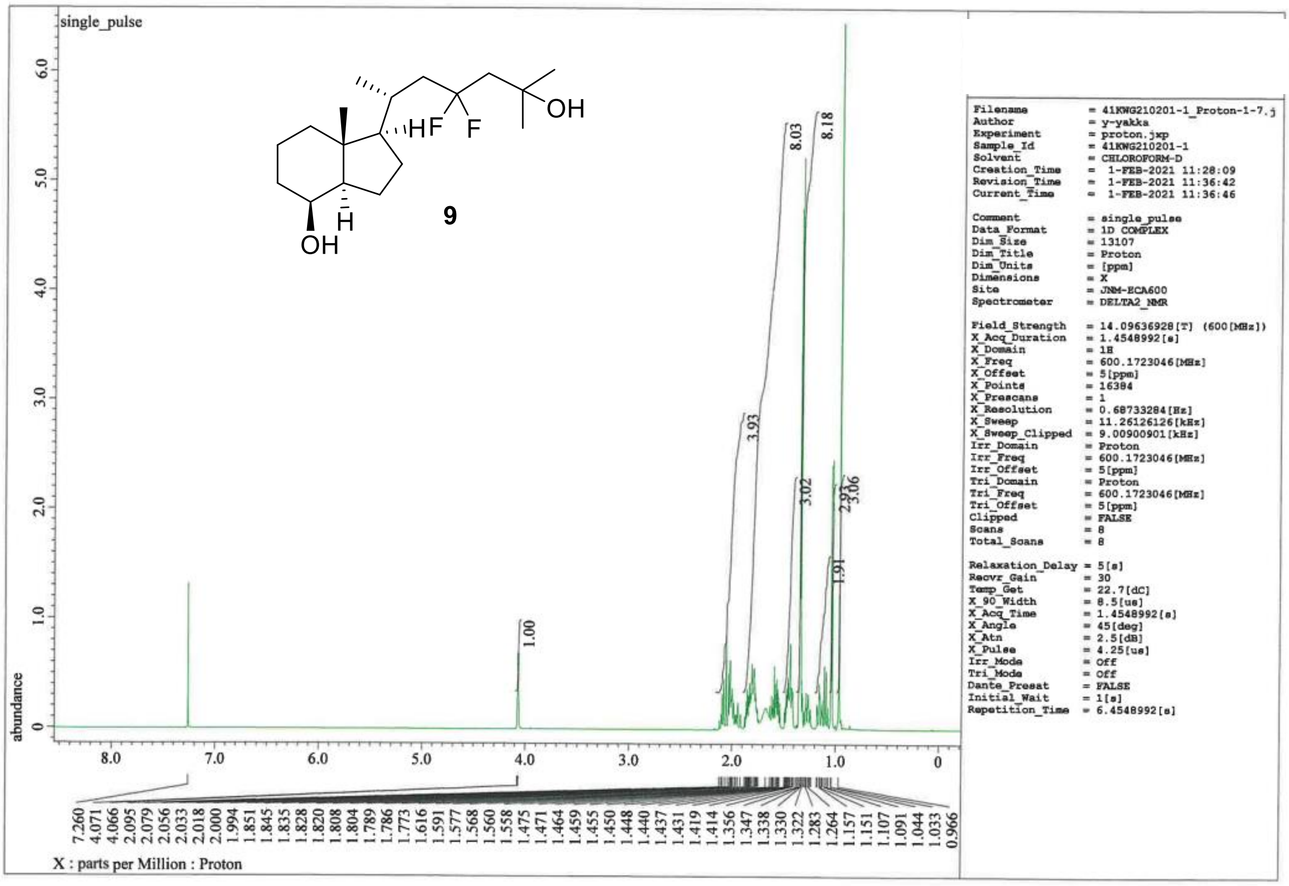
single_pulse
OH
HF F
OH
H
9
8.03
8.18
3.93
3.02
2.93
3.06
1.91
1.00
abundance
X : parts per Million : Proton
7.260 4.071 4.066 2.095 2.079 2.056 2.033 2.018 2.000 1.994 1.851 1.845 1.835 1.828 1.820 1.808 1.804 1.789 1.786 1.773 1.616 1.591 1.577 1.568 1.560 1.558 1.475 1.471 1.464 1.459 1.455 1.450 1.448 1.440 1.437 1.431 1.419 1.414 1.356 1.347 1.338 1.330 1.322 1.283 1.264 1.157 1.151 1.107 1.091 1.044 1.033 0.966
Filename = 41KWG210201-1_Proton-1-7.j
Author = y-yakka
Experiment = proton.jxp
Sample_Id = 41KWG210201-1
Solvent = CHLOROFORM-D
Creation_Time = 1-FEB-2021 11:28:09
Revision_Time = 1-FEB-2021 11:36:42
Current_Time = 1-FEB-2021 11:36:46
Comment = single_pulse
Data_Format = 1D COMPLEX
Dim_Size = 13107
Dim_Title = Proton
Dim_Units = [ppm]
Dimensions = X
Site = JNM-ECA600
Spectrometer = DELTA2_NMR
Field_Strength = 14.09636928[T] (600[MHz])
X_Acq_Duration = 1.4548992[s]
X_Domain = 1H
X_Freq = 600.1723046[MHz]
X_Offset = 5[ppm]
X_Points = 16384
X_Prescans = 1
X_Resolution = 0.68733284[Hz]
X_Sweep = 11.26126126[kHz]
X_Sweep_Clipped = 9.00900901[kHz]
Irr_Domain = Proton
Irr_Freq = 600.1723046[MHz]
Irr_Offset = 5[ppm]
Tri_Domain = Proton
Tri_Freq = 600.1723046[MHz]
Tri_Offset = 5[ppm]
Clipped = FALSE
Scans = 8
Total_Scans = 8
Relaxation_Delay = 5[s]
Recvr_Gain = 30
Temp_Get = 22.7[dC]
X_90_Width = 8.5[us]
X_Acq_Time = 1.4548992[s]
X_Angle = 45[deg]
X_Atn = 2.5[dB]
X_Pulse = 4.25[us]
Irr_Mode = Off
Tri_Mode = Off
Dante_Presat = FALSE
Initial_Wait = 1[s]
Repetition_Time = 6.4548992[s]

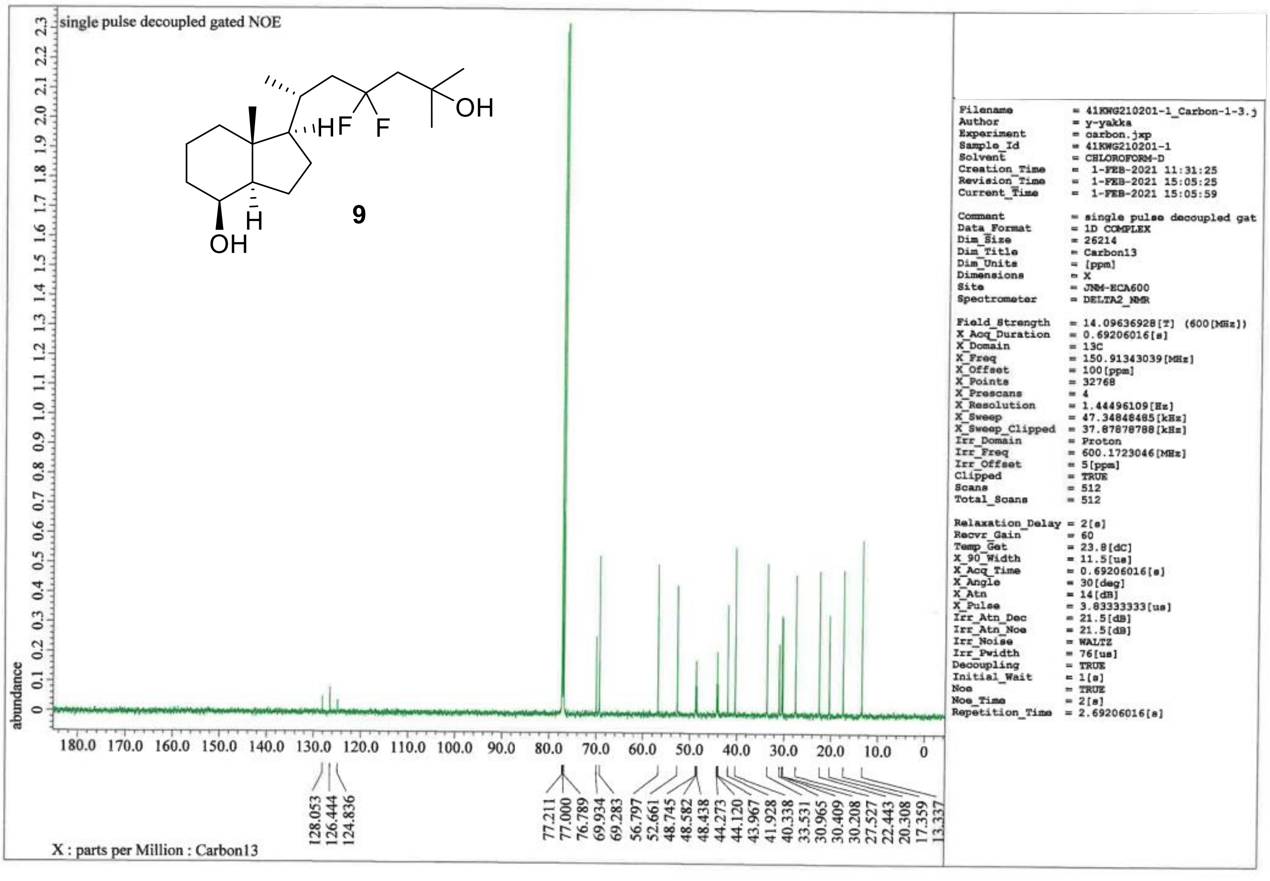
single pulse decoupled gated NOE
OH
HF F
OH
H
9
abundance
X : parts per Million : Carbon13
128.053 126.444 124.836
77.211 77.000 76.789 69.934 69.283 56.797 52.661 48.745 48.582 48.438 44.273 44.120 43.967 41.928 40.338 33.531 30.965 30.409 30.208 27.527 22.443 20.308 17.359 13.337
Filename = 41KWG210201-1_Carbon-1-3.j
Author = y-yakka
Experiment = carbon.jxp
Sample_Id = 41KWG210201-1
Solvent = CHLOROFORM-D
Creation_Time = 1-FEB-2021 11:31:25
Revision_Time = 1-FEB-2021 15:05:25
Current_Time = 1-FEB-2021 15:05:59
Comment = single pulse decoupled gat
Data_Format = 1D COMPLEX
Dim_Size = 26214
Dim_Title = Carbon13
Dim_Units = [ppm]
Dimensions = X
Site = JNM-ECA600
Spectrometer = DELTA2_NMR
Field_Strength = 14.09636928[T] (600[MHz])
X_Acq_Duration = 0.69206016[s]
X_Domain = 13C
X_Freq = 150.91343039[MHz]
X_Offset = 100[ppm]
X_Points = 32768
X_Prescans = 4
X_Resolution = 1.44496109[Hz]
X_Sweep = 47.34848485[kHz]
X_Sweep_Clipped = 37.87878788[kHz]
Irr_Domain = Proton
Irr_Freq = 600.1723046[MHz]
Irr_Offset = 5[ppm]
Clipped = TRUE
Scans = 512
Total_Scans = 512
Relaxation_Delay = 2[s]
Recvr_Gain = 60
Temp_Get = 23.8[dC]
X_90_Width = 11.5[us]
X_Acq_Time = 0.69206016[s]
X_Angle = 30[deg]
X_Atn = 14[dB]
X_Pulse = 3.83333333[us]
Irr_Atn_Dec = 21.5[dB]
Irr_Atn_Noe = 21.5[dB]
Irr_Noise = WALTZ
Irr_Pwidth = 76[us]
Decoupling = TRUE
Initial_Wait = 1[s]
Noe = TRUE
Noe_Time = 2[s]
Repetition_Time = 2.69206016[s]

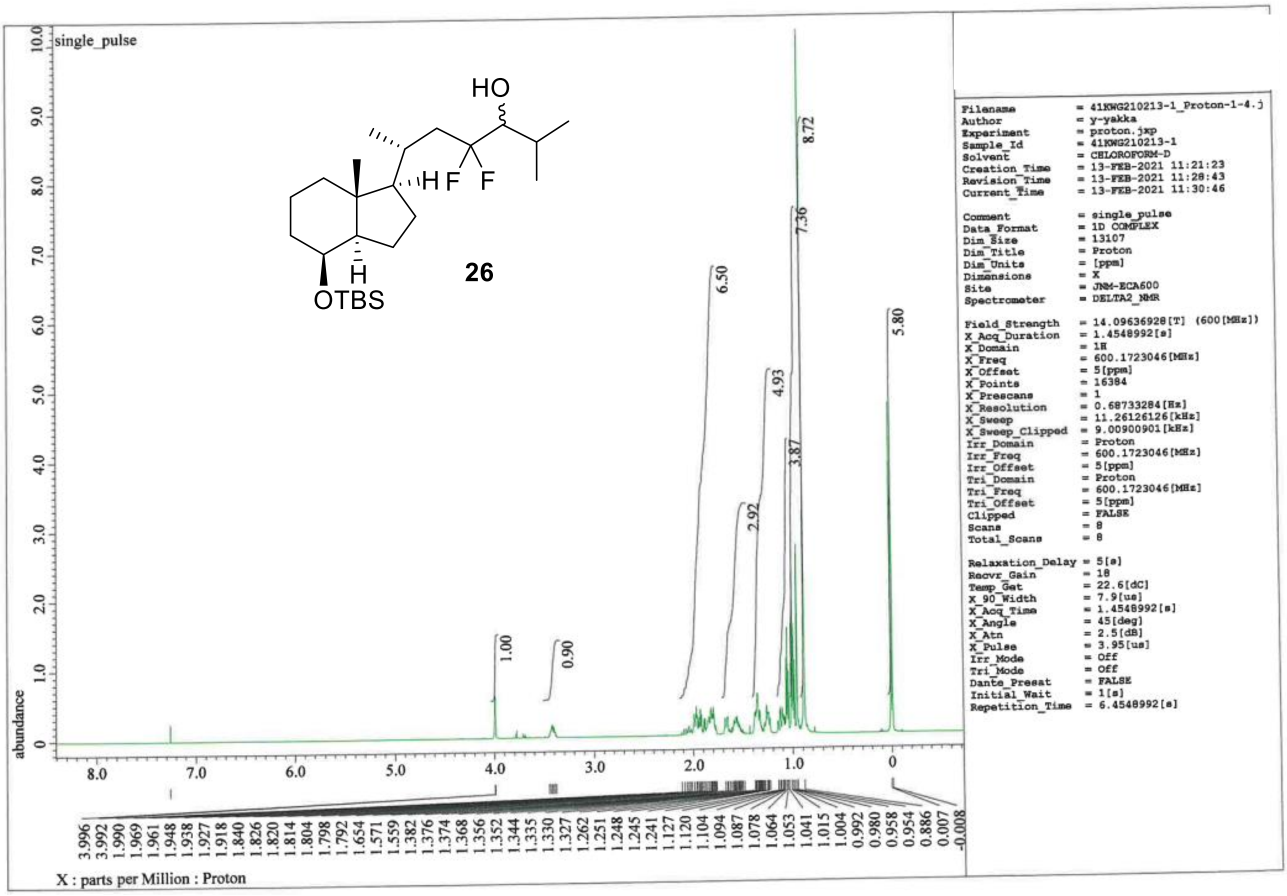
single_pulse
HO
H F F
26
OTBS
abundance
X : parts per Million : Proton
1.00
0.90
6.50
2.92
4.93
3.87
7.36
8.72
5.80
3.996
3.992
1.990
1.969
1.961
1.948
1.938
1.927
1.918
1.840
1.826
1.820
1.814
1.804
1.798
1.792
1.654
1.571
1.559
1.382
1.376
1.374
1.368
1.356
1.352
1.344
1.335
1.330
1.327
1.262
1.251
1.248
1.245
1.241
1.127
1.120
1.104
1.094
1.087
1.078
1.064
1.053
1.041
1.015
1.004
0.992
0.980
0.958
0.954
0.886
0.007
-0.008
Filename = 41KWG210213-1_Proton-1-4.j
Author = y-yakka
Experiment = proton.jxp
Sample_Id = 41KWG210213-1
Solvent = CHLOROFORM-D
Creation_Time = 13-FEB-2021 11:21:23
Revision_Time = 13-FEB-2021 11:28:43
Current_Time = 13-FEB-2021 11:30:46
Comment = single_pulse
Data_Format = 1D COMPLEX
Dim_Size = 13107
Dim_Title = Proton
Dim_Units = [ppm]
Dimensions = X
Site = JNM-ECA600
Spectrometer = DELTA2_NMR
Field_Strength = 14.09636928[T] (600[MHz])
X_Acq_Duration = 1.4548992[s]
X_Domain = 1H
X_Freq = 600.1723046[MHz]
X_Offset = 5[ppm]
X_Points = 16384
X_Prescans = 1
X_Resolution = 0.68733284[Hz]
X_Sweep = 11.26126126[kHz]
X_Sweep_Clipped = 9.00900901[kHz]
Irr_Domain = Proton
Irr_Freq = 600.1723046[MHz]
Irr_Offset = 5[ppm]
Tri_Domain = Proton
Tri_Freq = 600.1723046[MHz]
Tri_Offset = 5[ppm]
Clipped = FALSE
Scans = 8
Total_Scans = 8
Relaxation_Delay = 5[s]
Recvr_Gain = 18
Temp_Get = 22.6[dC]
X_90_Width = 7.9[us]
X_Acq_Time = 1.4548992[s]
X_Angle = 45[deg]
X_Atn = 2.5[dB]
X_Pulse = 3.95[us]
Irr_Mode = Off
Tri_Mode = Off
Dante_Presat = FALSE
Initial_Wait = 1[s]
Repetition_Time = 6.4548992[s]

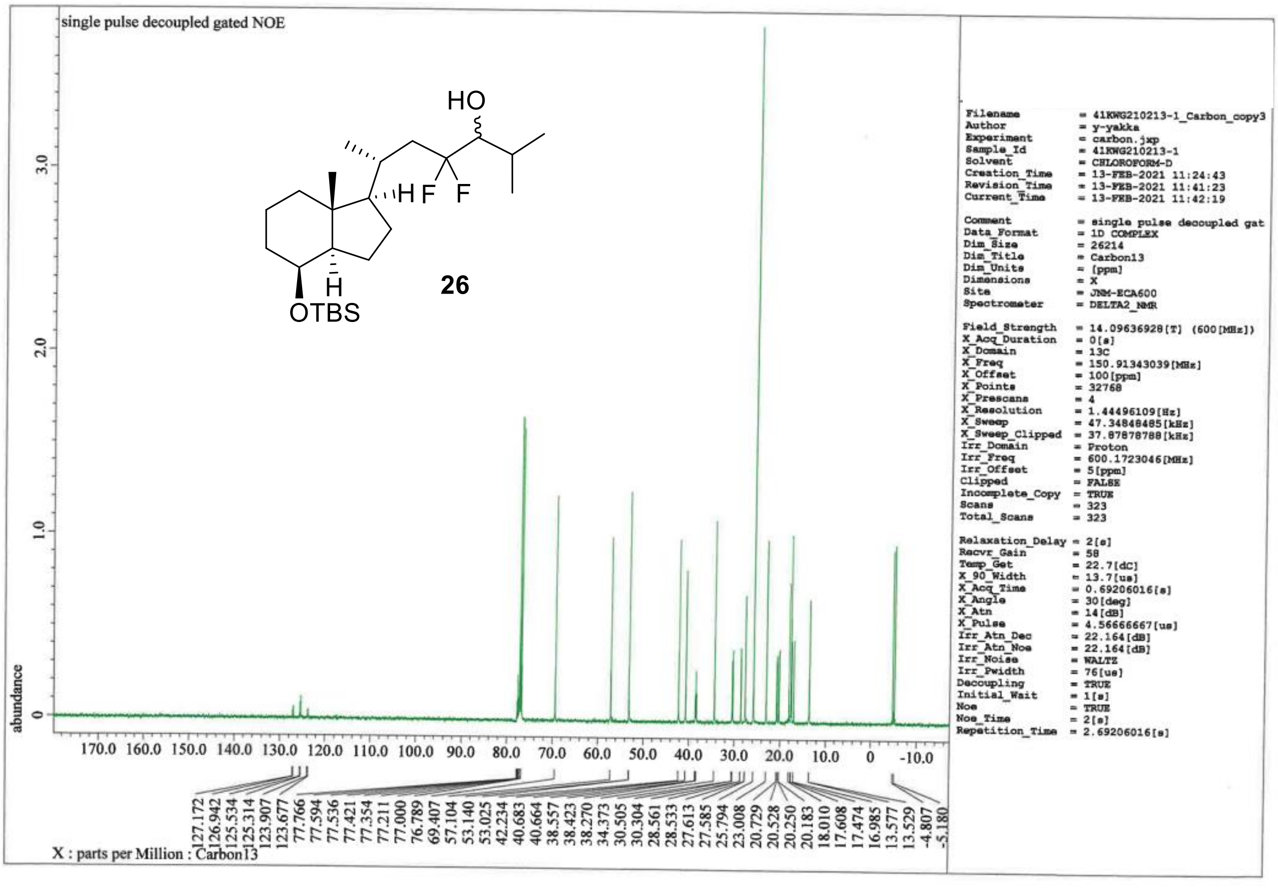
single pulse decoupled gated NOE
HO
H F F
26
OTBS
abundance
X : parts per Million : Carbon13
127.172
126.942
125.534
125.314
123.907
123.677
77.766
77.594
77.536
77.421
77.354
77.211
77.000
76.789
69.407
57.104
53.140
53.025
42.234
40.683
40.664
38.557
38.423
38.270
34.373
30.505
30.304
28.561
28.533
27.613
27.585
25.794
23.008
20.729
20.528
20.250
20.183
18.010
17.608
17.474
16.985
13.577
13.529
-4.807
-5.180
Filename = 41KWG210213-1_Carbon_copy3
Author = y-yakka
Experiment = carbon.jxp
Sample_Id = 41KWG210213-1
Solvent = CHLOROFORM-D
Creation_Time = 13-FEB-2021 11:24:43
Revision_Time = 13-FEB-2021 11:41:23
Current_Time = 13-FEB-2021 11:42:19
Comment = single pulse decoupled gat
Data_Format = 1D COMPLEX
Dim_Size = 26214
Dim_Title = Carbon13
Dim_Units = [ppm]
Dimensions = X
Site = JNM-ECA600
Spectrometer = DELTA2_NMR
Field_Strength = 14.09636928[T] (600[MHz])
X_Acq_Duration = 0[s]
X_Domain = 13C
X_Freq = 150.91343039[MHz]
X_Offset = 100[ppm]
X_Points = 32768
X_Prescans = 4
X_Resolution = 1.44496109[Hz]
X_Sweep = 47.34848485[kHz]
X_Sweep_Clipped = 37.87878788[kHz]
Irr_Domain = Proton
Irr_Freq = 600.1723046[MHz]
Irr_Offset = 5[ppm]
Clipped = FALSE
Incomplete_Copy = TRUE
Scans = 323
Total_Scans = 323
Relaxation_Delay = 2[s]
Recvr_Gain = 58
Temp_Get = 22.7[dC]
X_90_Width = 13.7[us]
X_Acq_Time = 0.69206016[s]
X_Angle = 30[deg]
X_Atn = 14[dB]
X_Pulse = 4.56666667[us]
Irr_Atn_Dec = 22.164[dB]
Irr_Atn_Noe = 22.164[dB]
Irr_Noise = WALTZ
Irr_Pwidth = 76[us]
Decoupling = TRUE
Initial_Wait = 1[s]
Noe = TRUE
Noe_Time = 2[s]
Repetition_Time = 2.69206016[s]

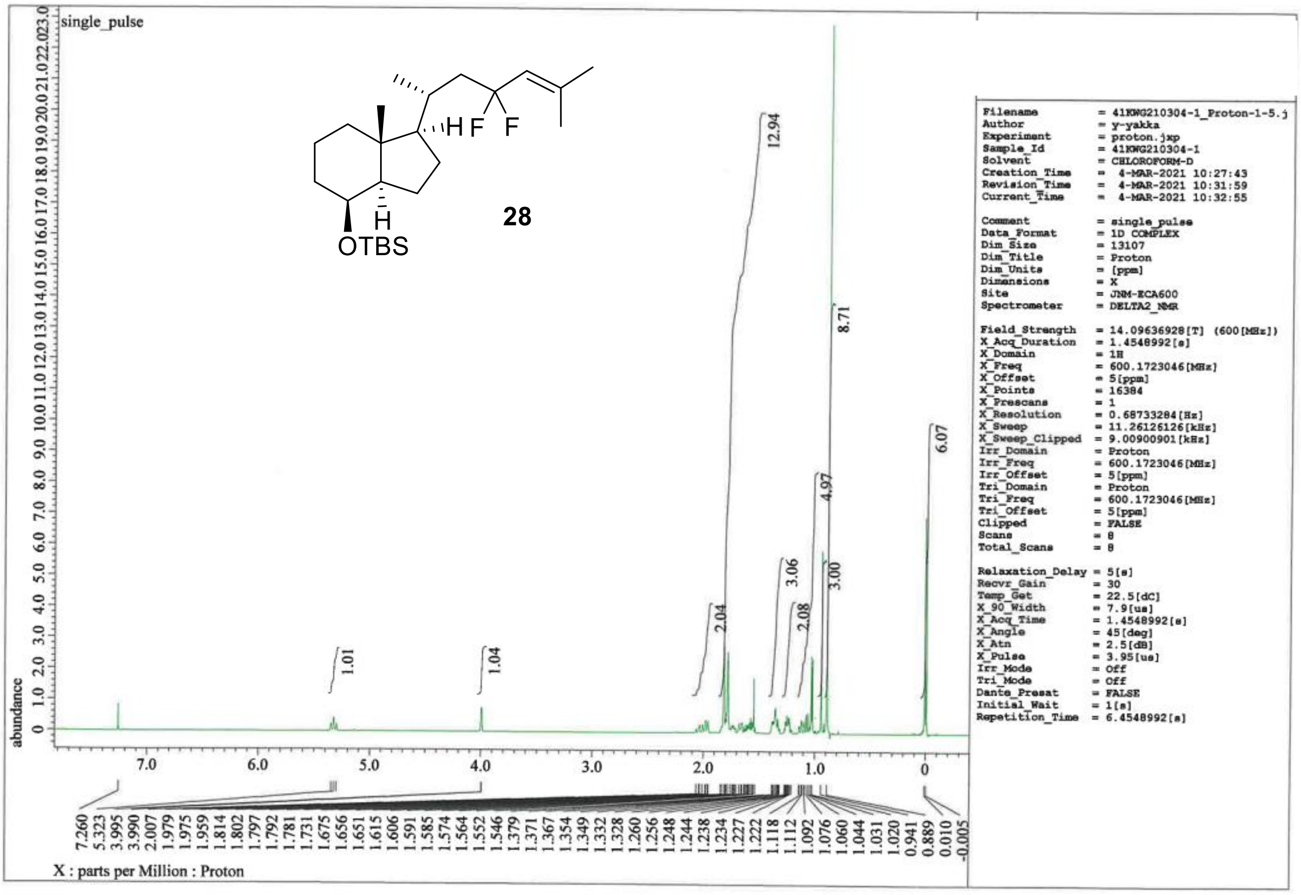
single_pulse
28
OTBS
abundance
X : parts per Million : Proton
12.94
8.71
6.07
4.97
3.06
3.00
2.08
2.04
1.04
1.01
7.260
5.323
3.995
3.990
2.007
1.979
1.975
1.959
1.814
1.802
1.797
1.792
1.781
1.731
1.675
1.656
1.651
1.615
1.606
1.591
1.585
1.574
1.564
1.552
1.546
1.379
1.371
1.367
1.354
1.349
1.332
1.328
1.260
1.256
1.248
1.244
1.238
1.234
1.227
1.222
1.118
1.112
1.092
1.076
1.060
1.044
1.031
1.020
0.941
0.889
0.010
-0.005
Filename = 41KWG210304-1_Proton-1-5.j
Author = y-yakka
Experiment = proton.jxp
Sample_Id = 41KWG210304-1
Solvent = CHLOROFORM-D
Creation_Time = 4-MAR-2021 10:27:43
Revision_Time = 4-MAR-2021 10:31:59
Current_Time = 4-MAR-2021 10:32:55
Comment = single_pulse
Data_Format = 1D COMPLEX
Dim_Size = 13107
Dim_Title = Proton
Dim_Units = [ppm]
Dimensions = X
Site = JNM-ECA600
Spectrometer = DELTA2_NMR
Field_Strength = 14.09636928[T] (600[MHz])
X_Acq_Duration = 1.4548992[s]
X_Domain = 1H
X_Freq = 600.1723046[MHz]
X_Offset = 5[ppm]
X_Points = 16384
X_Prescans = 1
X_Resolution = 0.68733284[Hz]
X_Sweep = 11.26126126[kHz]
X_Sweep_Clipped = 9.00900901[kHz]
Irr_Domain = Proton
Irr_Freq = 600.1723046[MHz]
Irr_Offset = 5[ppm]
Tri_Domain = Proton
Tri_Freq = 600.1723046[MHz]
Tri_Offset = 5[ppm]
Clipped = FALSE
Scans = 8
Total_Scans = 8
Relaxation_Delay = 5[s]
Recvr_Gain = 30
Temp_Get = 22.5[dC]
X_90_Width = 7.9[us]
X_Acq_Time = 1.4548992[s]
X_Angle = 45[deg]
X_Atn = 2.5[dB]
X_Pulse = 3.95[us]
Irr_Mode = Off
Tri_Mode = Off
Dante_Presat = FALSE
Initial_Wait = 1[s]
Repetition_Time = 6.4548992[s]

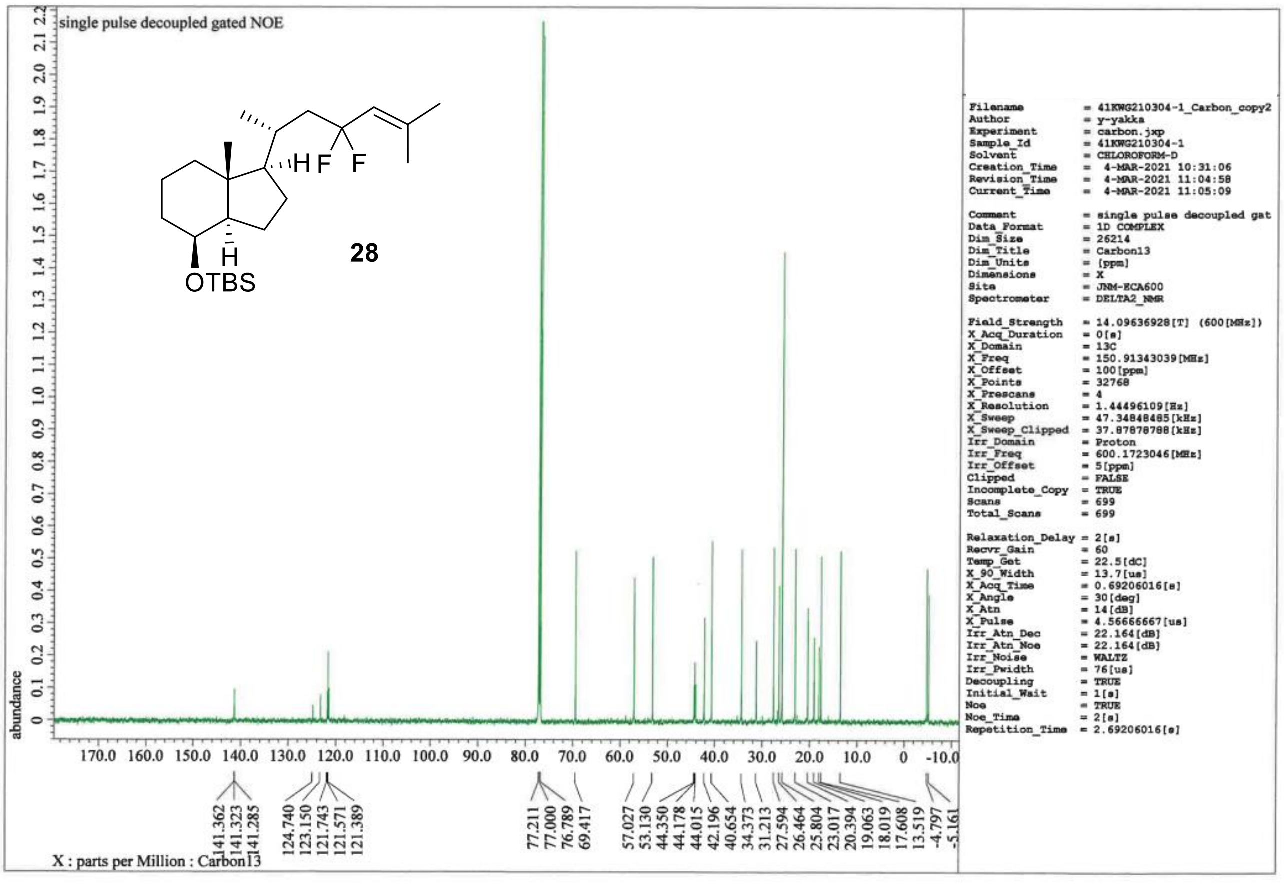
single pulse decoupled gated NOE
28
OTBS
abundance
X : parts per Million : Carbon13
141.362
141.323
141.285
124.740
123.150
121.743
121.571
121.389
77.211
77.000
76.789
69.417
57.027
53.130
44.350
44.178
44.015
42.196
40.654
34.373
31.213
27.594
26.464
25.804
23.017
20.394
19.063
18.019
17.608
13.519
-4.797
-5.161
Filename = 41KWG210304-1_Carbon_copy2
Author = y-yakka
Experiment = carbon.jxp
Sample_Id = 41KWG210304-1
Solvent = CHLOROFORM-D
Creation_Time = 4-MAR-2021 10:31:06
Revision_Time = 4-MAR-2021 11:04:58
Current_Time = 4-MAR-2021 11:05:09
Comment = single pulse decoupled gat
Data_Format = 1D COMPLEX
Dim_Size = 26214
Dim_Title = Carbon13
Dim_Units = [ppm]
Dimensions = X
Site = JNM-ECA600
Spectrometer = DELTA2_NMR
Field_Strength = 14.09636928[T] (600[MHz])
X_Acq_Duration = 0[s]
X_Domain = 13C
X_Freq = 150.91343039[MHz]
X_Offset = 100[ppm]
X_Points = 32768
X_Prescans = 4
X_Resolution = 1.44496109[Hz]
X_Sweep = 47.34848485[kHz]
X_Sweep_Clipped = 37.87878788[kHz]
Irr_Domain = Proton
Irr_Freq = 600.1723046[MHz]
Irr_Offset = 5[ppm]
Clipped = FALSE
Incomplete_Copy = TRUE
Scans = 699
Total_Scans = 699
Relaxation_Delay = 2[s]
Recvr_Gain = 60
Temp_Get = 22.5[dC]
X_90_Width = 13.7[us]
X_Acq_Time = 0.69206016[s]
X_Angle = 30[deg]
X_Atn = 14[dB]
X_Pulse = 4.56666667[us]
Irr_Atn_Dec = 22.164[dB]
Irr_Atn_Noe = 22.164[dB]
Irr_Noise = WALTZ
Irr_Pwidth = 76[us]
Decoupling = TRUE
Initial_Wait = 1[s]
Noe = TRUE
Noe_Time = 2[s]
Repetition_Time = 2.69206016[s]

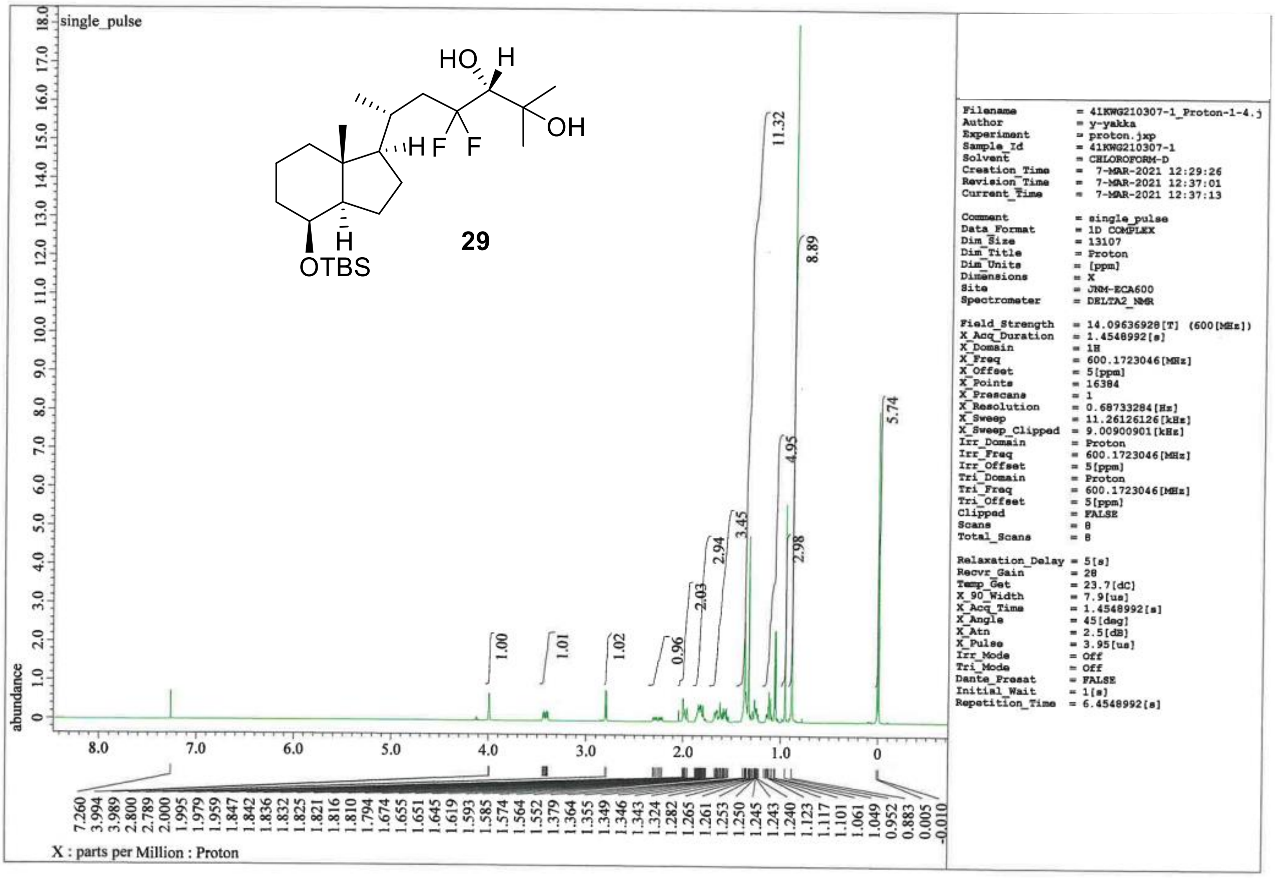
single_pulse
HO H
OH
H F F
H
OTBS
29
abundance
X : parts per Million : Proton
Filename = 41KWG210307-1_Proton-1-4.j
Author = y-yakka
Experiment = proton.jxp
Sample_Id = 41KWG210307-1
Solvent = CHLOROFORM-D
Creation_Time = 7-MAR-2021 12:29:26
Revision_Time = 7-MAR-2021 12:37:01
Current_Time = 7-MAR-2021 12:37:13
Comment = single_pulse
Data_Format = 1D COMPLEX
Dim_Size = 13107
Dim_Title = Proton
Dim_Units = [ppm]
Dimensions = X
Site = JNM-ECA600
Spectrometer = DELTA2_NMR
Field_Strength = 14.09636928[T] (600[MHz])
X_Acq_Duration = 1.4548992[s]
X_Domain = 1H
X_Freq = 600.1723046[MHz]
X_Offset = 5[ppm]
X_Points = 16384
X_Prescans = 1
X_Resolution = 0.68733284[Hz]
X_Sweep = 11.26126126[kHz]
X_Sweep_Clipped = 9.00900901[kHz]
Irr_Domain = Proton
Irr_Freq = 600.1723046[MHz]
Irr_Offset = 5[ppm]
Tri_Domain = Proton
Tri_Freq = 600.1723046[MHz]
Tri_Offset = 5[ppm]
Clipped = FALSE
Scans = 8
Total_Scans = 8
Relaxation_Delay = 5[s]
Recvr_Gain = 28
Temp_Get = 23.7[dC]
X_90_Width = 7.9[us]
X_Acq_Time = 1.4548992[s]
X_Angle = 45[deg]
X_Atn = 2.5[dB]
X_Pulse = 3.95[us]
Irr_Mode = Off
Tri_Mode = Off
Dante_Presat = FALSE
Initial_Wait = 1[s]
Repetition_Time = 6.4548992[s]

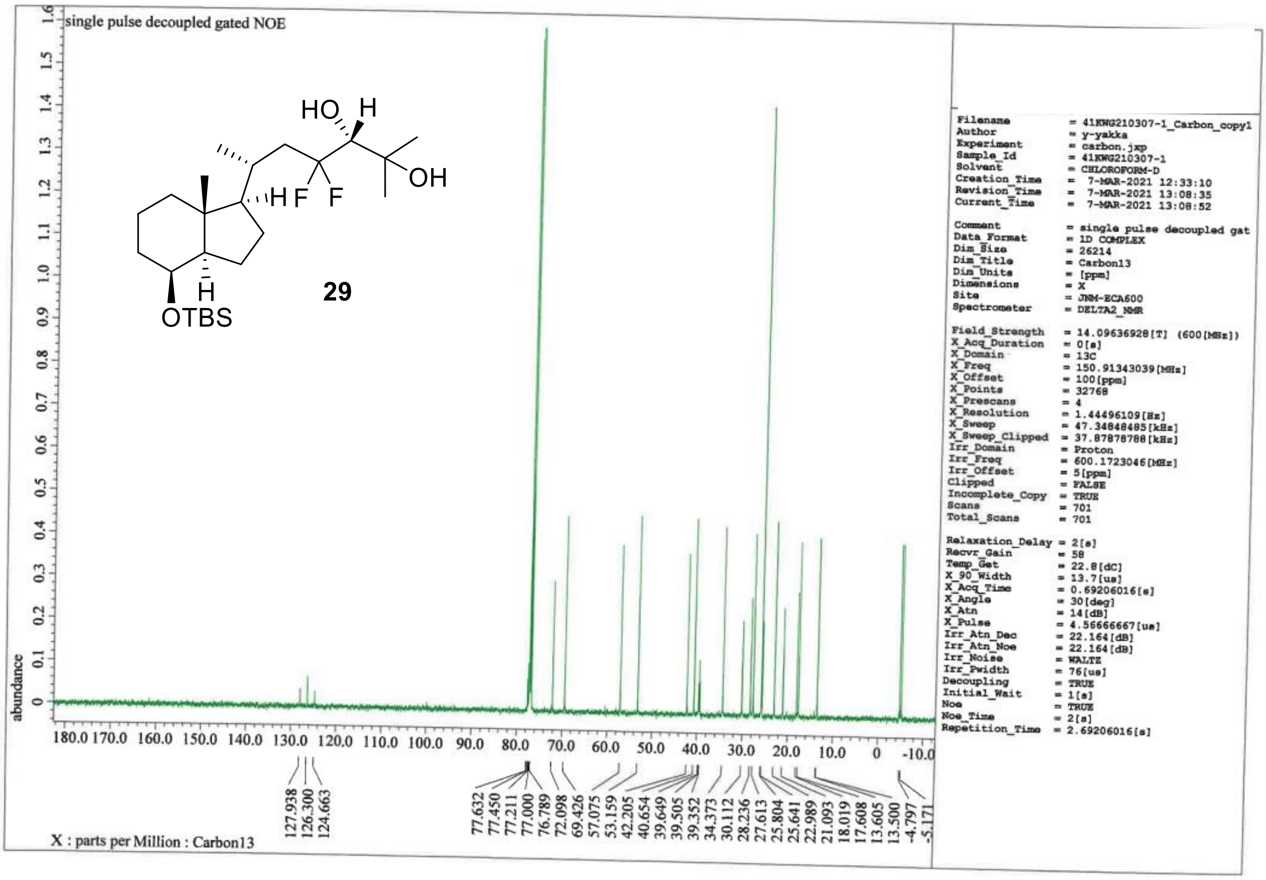
single pulse decoupled gated NOE
HO H
OH
H F F
H
OTBS
29
abundance
X : parts per Million : Carbon13
Filename = 41KWG210307-1_Carbon_copy1
Author = y-yakka
Experiment = carbon.jxp
Sample_Id = 41KWG210307-1
Solvent = CHLOROFORM-D
Creation_Time = 7-MAR-2021 12:33:10
Revision_Time = 7-MAR-2021 13:08:35
Current_Time = 7-MAR-2021 13:08:52
Comment = single pulse decoupled gat
Data_Format = 1D COMPLEX
Dim_Size = 26214
Dim_Title = Carbon13
Dim_Units = [ppm]
Dimensions = X
Site = JNM-ECA600
Spectrometer = DELTA2_NMR
Field_Strength = 14.09636928[T] (600[MHz])
X_Acq_Duration = 0[s]
X_Domain = 13C
X_Freq = 150.91343039[MHz]
X_Offset = 100[ppm]
X_Points = 32768
X_Prescans = 4
X_Resolution = 1.44496109[Hz]
X_Sweep = 47.34848485[kHz]
X_Sweep_Clipped = 37.87878788[kHz]
Irr_Domain = Proton
Irr_Freq = 600.1723046[MHz]
Irr_Offset = 5[ppm]
Clipped = FALSE
Incomplete_Copy = TRUE
Scans = 701
Total_Scans = 701
Relaxation_Delay = 2[s]
Recvr_Gain = 58
Temp_Get = 22.8[dC]
X_90_Width = 13.7[us]
X_Acq_Time = 0.69206016[s]
X_Angle = 30[deg]
X_Atn = 14[dB]
X_Pulse = 4.56666667[us]
Irr_Atn_Dec = 22.164[dB]
Irr_Atn_Noe = 22.164[dB]
Irr_Noise = WALTZ
Irr_Pwidth = 76[us]
Decoupling = TRUE
Initial_Wait = 1[s]
Noe = TRUE
Noe_Time = 2[s]
Repetition_Time = 2.69206016[s]

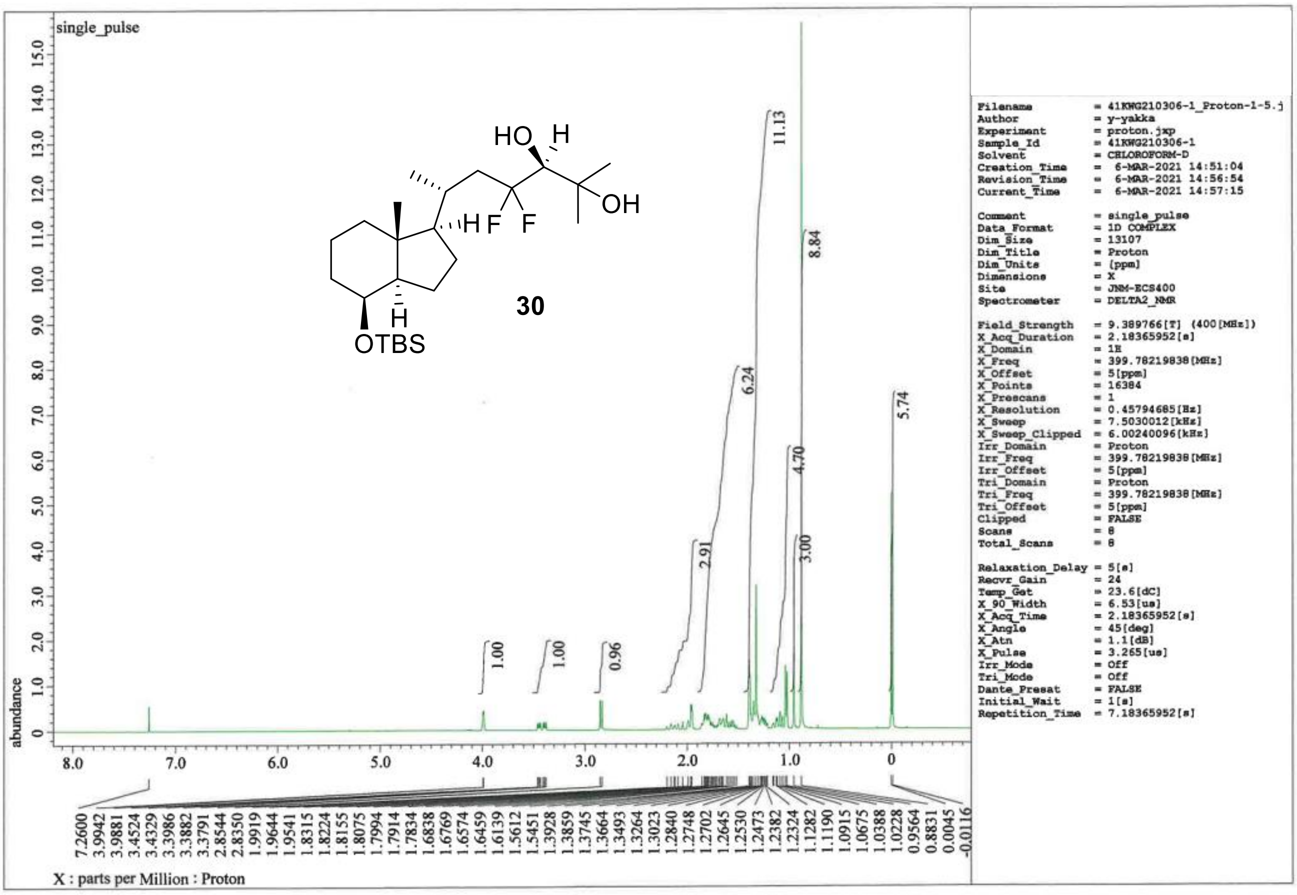
single_pulse
HO H
OH
H F F
H
OTBS
30
1.00
1.00
0.96
2.91
6.24
11.13
4.70
3.00
8.84
5.74
abundance
X : parts per Million : Proton
7.2600 3.9942 3.9881 3.4524 3.4329 3.3986 3.3882 3.3791 2.8544 2.8350 1.9919 1.9644 1.9541 1.8315 1.8224 1.8155 1.8075 1.7994 1.7914 1.7834 1.6838 1.6769 1.6574 1.6459 1.6139 1.5612 1.5451 1.3928 1.3859 1.3745 1.3664 1.3493 1.3264 1.3023 1.2840 1.2748 1.2702 1.2645 1.2530 1.2473 1.2382 1.2324 1.1282 1.1190 1.0915 1.0675 1.0388 1.0228 0.9564 0.8831 0.0045 -0.0116
Filename = 41KWG210306-1_Proton-1-5.j
Author = y-yakka
Experiment = proton.jxp
Sample_Id = 41KWG210306-1
Solvent = CHLOROFORM-D
Creation_Time = 6-MAR-2021 14:51:04
Revision_Time = 6-MAR-2021 14:56:54
Current_Time = 6-MAR-2021 14:57:15
Comment = single_pulse
Data_Format = 1D COMPLEX
Dim_Size = 13107
Dim_Title = Proton
Dim_Units = [ppm]
Dimensions = X
Site = JNM-ECS400
Spectrometer = DELTA2_NMR
Field_Strength = 9.389766[T] (400[MHz])
X_Acq_Duration = 2.18365952[s]
X_Domain = 1H
X_Freq = 399.78219838[MHz]
X_Offset = 5[ppm]
X_Points = 16384
X_Prescans = 1
X_Resolution = 0.45794685[Hz]
X_Sweep = 7.5030012[kHz]
X_Sweep_Clipped = 6.00240096[kHz]
Irr_Domain = Proton
Irr_Freq = 399.78219838[MHz]
Irr_Offset = 5[ppm]
Tri_Domain = Proton
Tri_Freq = 399.78219838[MHz]
Tri_Offset = 5[ppm]
Clipped = FALSE
Scans = 8
Total_Scans = 8
Relaxation_Delay = 5[s]
Recvr_Gain = 24
Temp_Get = 23.6[dC]
X_90_Width = 6.53[us]
X_Acq_Time = 2.18365952[s]
X_Angle = 45[deg]
X_Atn = 1.1[dB]
X_Pulse = 3.265[us]
Irr_Mode = Off
Tri_Mode = Off
Dante_Presat = FALSE
Initial_Wait = 1[s]
Repetition_Time = 7.18365952[s]

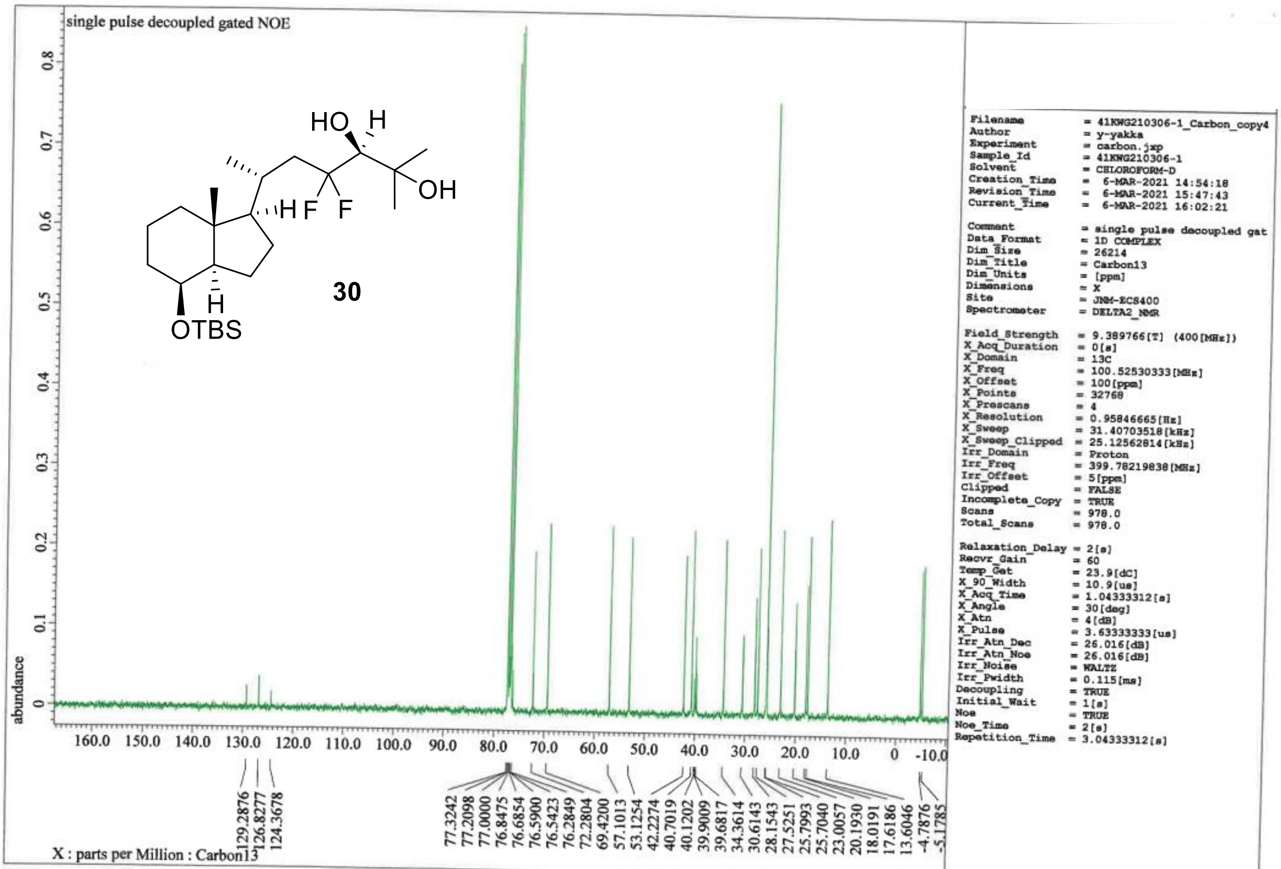
single pulse decoupled gated NOE
HO H
OH
H F F
H
OTBS
30
abundance
X : parts per Million : Carbon13
129.2876 126.8277 124.3678 77.3242 77.2098 77.0000 76.8475 76.6854 76.5900 76.5423 76.2849 72.2804 69.4200 57.1013 53.1254 42.2274 40.7019 40.1202 39.9009 39.6817 34.3614 30.6143 28.1543 27.5251 25.7993 25.7040 23.0057 20.1930 18.0191 17.6186 13.6046 -4.7876 -5.1785
Filename = 41KWG210306-1_Carbon_copy4
Author = y-yakka
Experiment = carbon.jxp
Sample_Id = 41KWG210306-1
Solvent = CHLOROFORM-D
Creation_Time = 6-MAR-2021 14:54:18
Revision_Time = 6-MAR-2021 15:47:43
Current_Time = 6-MAR-2021 16:02:21
Comment = single pulse decoupled gat
Data_Format = 1D COMPLEX
Dim_Size = 26214
Dim_Title = Carbon13
Dim_Units = [ppm]
Dimensions = X
Site = JNM-ECS400
Spectrometer = DELTA2_NMR
Field_Strength = 9.389766[T] (400[MHz])
X_Acq_Duration = 0[s]
X_Domain = 13C
X_Freq = 100.52530333[MHz]
X_Offset = 100[ppm]
X_Points = 32768
X_Prescans = 4
X_Resolution = 0.95846665[Hz]
X_Sweep = 31.40703518[kHz]
X_Sweep_Clipped = 25.12562814[kHz]
Irr_Domain = Proton
Irr_Freq = 399.78219838[MHz]
Irr_Offset = 5[ppm]
Clipped = FALSE
Incomplete_Copy = TRUE
Scans = 978.0
Total_Scans = 978.0
Relaxation_Delay = 2[s]
Recvr_Gain = 60
Temp_Get = 23.9[dC]
X_90_Width = 10.9[us]
X_Acq_Time = 1.04333312[s]
X_Angle = 30[deg]
X_Atn = 4[dB]
X_Pulse = 3.63333333[us]
Irr_Atn_Dec = 26.016[dB]
Irr_Atn_Noe = 26.016[dB]
Irr_Noise = WALTZ
Irr_Pwidth = 0.115[ms]
Decoupling = TRUE
Initial_Wait = 1[s]
Noe = TRUE
Noe_Time = 2[s]
Repetition_Time = 3.04333312[s]

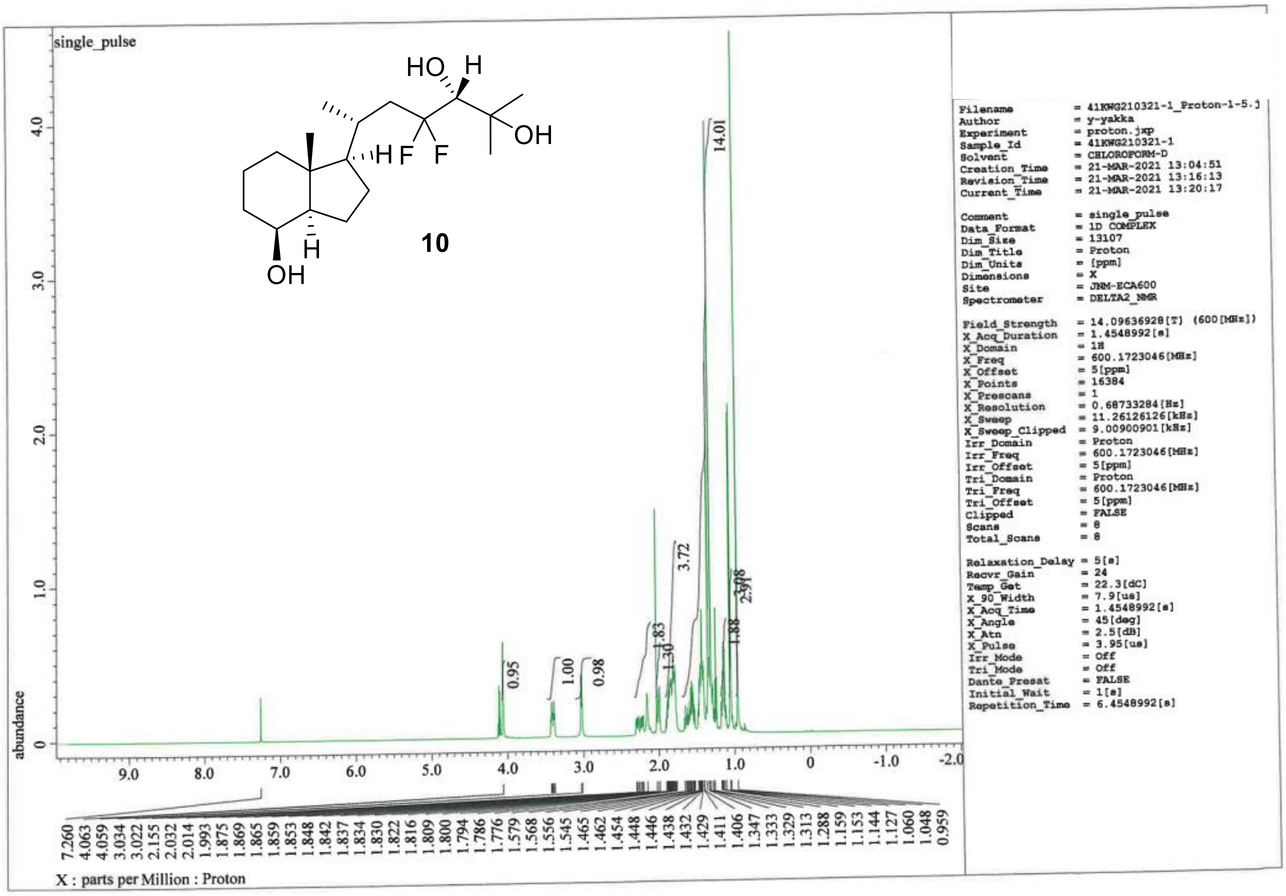

single_pulse
HO H
OH
H F F
H
OH
10
14.01
3.72
0.95
1.00
0.98
1.83
1.30
1.88
2.98
2.91
abundance
X : parts per Million : Proton
7.260
4.063
4.059
3.034
3.022
2.155
2.032
2.014
1.993
1.875
1.869
1.865
1.859
1.853
1.848
1.842
1.837
1.834
1.830
1.822
1.816
1.809
1.800
1.794
1.786
1.776
1.579
1.568
1.556
1.545
1.465
1.462
1.454
1.448
1.446
1.438
1.432
1.429
1.411
1.406
1.347
1.333
1.329
1.313
1.288
1.159
1.153
1.144
1.127
1.060
1.048
0.959
Filename = 41KWG210321-1_Proton-1-5.j
Author = y-yakka
Experiment = proton.jxp
Sample_Id = 41KWG210321-1
Solvent = CHLOROFORM-D
Creation_Time = 21-MAR-2021 13:04:51
Revision_Time = 21-MAR-2021 13:16:13
Current_Time = 21-MAR-2021 13:20:17
Comment = single_pulse
Data_Format = 1D COMPLEX
Dim_Size = 13107
Dim_Title = Proton
Dim_Units = [ppm]
Dimensions = X
Site = JNM-ECA600
Spectrometer = DELTA2_NMR
Field_Strength = 14.09636928[T] (600[MHz])
X_Acq_Duration = 1.4548992[s]
X_Domain = 1H
X_Freq = 600.1723046[MHz]
X_Offset = 5[ppm]
X_Points = 16384
X_Prescans = 1
X_Resolution = 0.68733284[Hz]
X_Sweep = 11.26126126[kHz]
X_Sweep_Clipped = 9.00900901[kHz]
Irr_Domain = Proton
Irr_Freq = 600.1723046[MHz]
Irr_Offset = 5[ppm]
Tri_Domain = Proton
Tri_Freq = 600.1723046[MHz]
Tri_Offset = 5[ppm]
Clipped = FALSE
Scans = 8
Total_Scans = 8
Relaxation_Delay = 5[s]
Recvr_Gain = 24
Temp_Get = 22.3[dC]
X_90_Width = 7.9[us]
X_Acq_Time = 1.4548992[s]
X_Angle = 45[deg]
X_Atn = 2.5[dB]
X_Pulse = 3.95[us]
Irr_Mode = Off
Tri_Mode = Off
Dante_Presat = FALSE
Initial_Wait = 1[s]
Repetition_Time = 6.4548992[s]

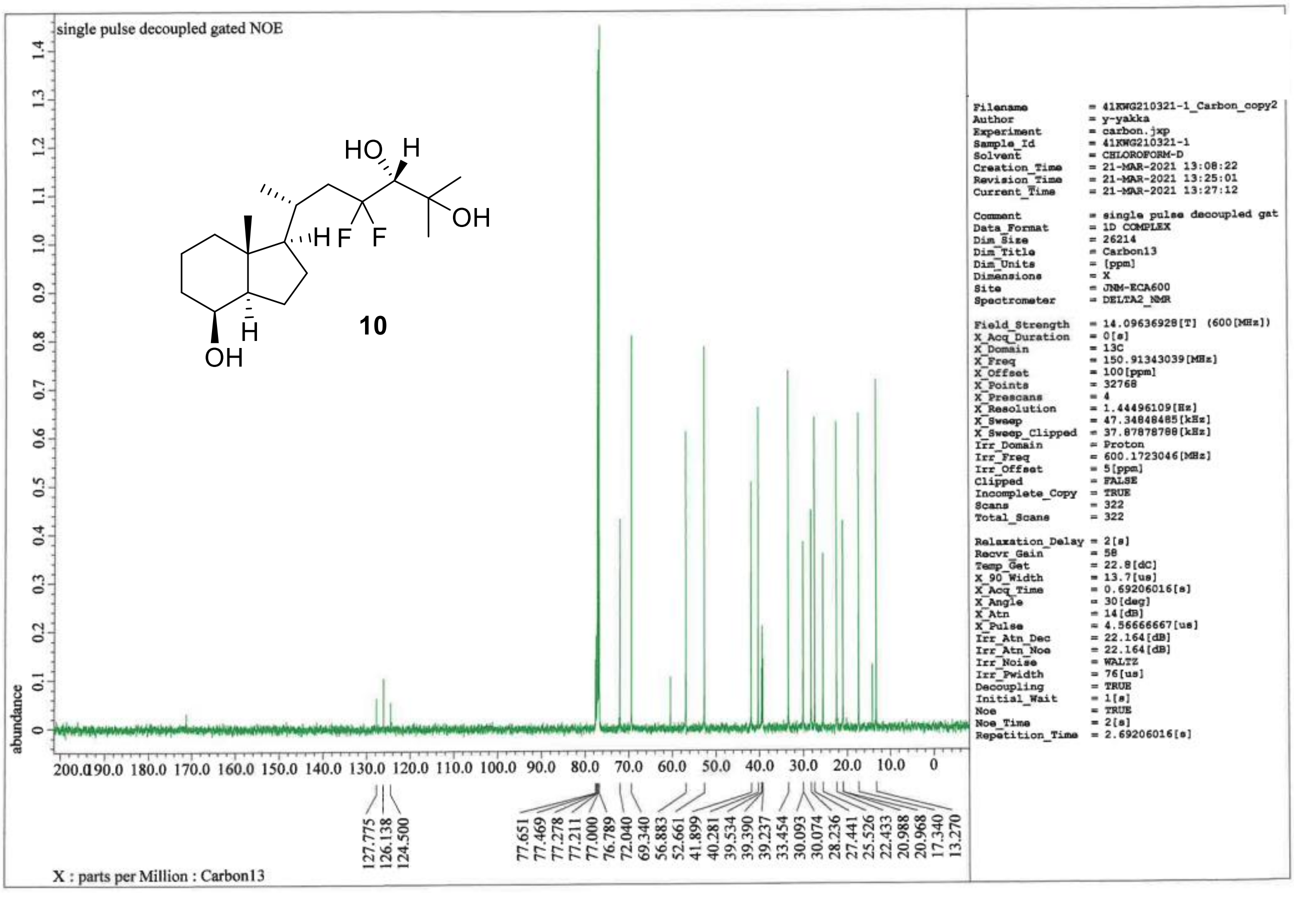

single pulse decoupled gated NOE
HO H
OH
H F F
H
OH
10
abundance
X : parts per Million : Carbon13
127.775
126.138
124.500
77.651
77.469
77.278
77.211
77.000
76.789
72.040
69.340
56.883
52.661
41.899
40.281
39.534
39.390
39.237
33.454
30.093
30.074
28.236
27.441
25.526
22.433
20.988
20.968
17.340
13.270
Filename = 41KWG210321-1_Carbon_copy2
Author = y-yakka
Experiment = carbon.jxp
Sample_Id = 41KWG210321-1
Solvent = CHLOROFORM-D
Creation_Time = 21-MAR-2021 13:08:22
Revision_Time = 21-MAR-2021 13:25:01
Current_Time = 21-MAR-2021 13:27:12
Comment = single pulse decoupled gat
Data_Format = 1D COMPLEX
Dim_Size = 26214
Dim_Title = Carbon13
Dim_Units = [ppm]
Dimensions = X
Site = JNM-ECA600
Spectrometer = DELTA2_NMR
Field_Strength = 14.09636928[T] (600[MHz])
X_Acq_Duration = 0[s]
X_Domain = 13C
X_Freq = 150.91343039[MHz]
X_Offset = 100[ppm]
X_Points = 32768
X_Prescans = 4
X_Resolution = 1.44496109[Hz]
X_Sweep = 47.34848485[kHz]
X_Sweep_Clipped = 37.87878788[kHz]
Irr_Domain = Proton
Irr_Freq = 600.1723046[MHz]
Irr_Offset = 5[ppm]
Clipped = FALSE
Incomplete_Copy = TRUE
Scans = 322
Total_Scans = 322
Relaxation_Delay = 2[s]
Recvr_Gain = 58
Temp_Get = 22.8[dC]
X_90_Width = 13.7[us]
X_Acq_Time = 0.69206016[s]
X_Angle = 30[deg]
X_Atn = 14[dB]
X_Pulse = 4.56666667[us]
Irr_Atn_Dec = 22.164[dB]
Irr_Atn_Noe = 22.164[dB]
Irr_Noise = WALTZ
Irr_Pwidth = 76[us]
Decoupling = TRUE
Initial_Wait = 1[s]
Noe = TRUE
Noe_Time = 2[s]
Repetition_Time = 2.69206016[s]

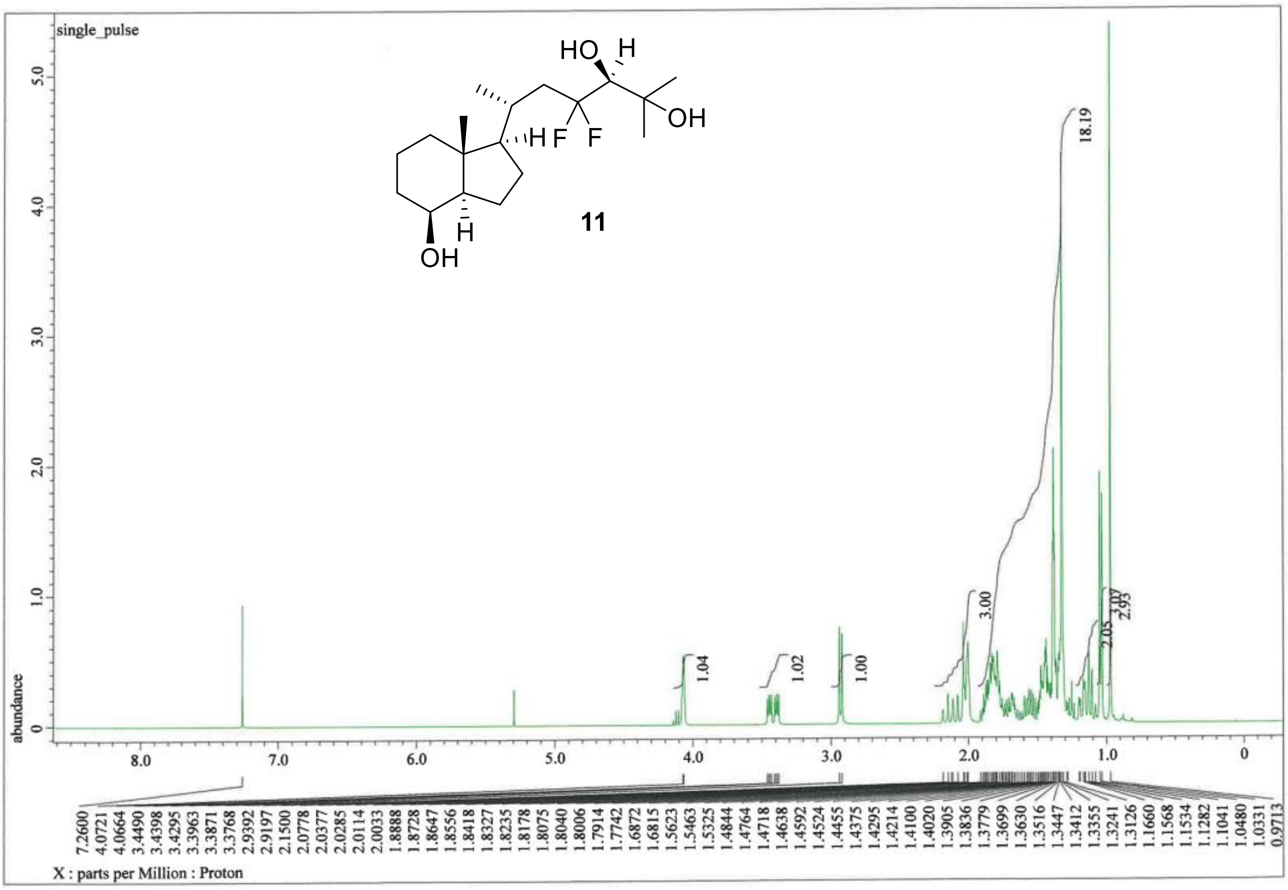
single_pulse
HO H
OH
H F F
H
OH
11
18.19
3.00
2.05
2.07
2.95
1.04
1.02
1.00
abundance
0
1.0
2.0
3.0
4.0
5.0
8.0
7.0
6.0
5.0
4.0
3.0
2.0
1.0
0
7.2600
4.0721
4.0664
3.4490
3.4398
3.4295
3.3963
3.3871
3.3768
2.9392
2.9197
2.1500
2.0778
2.0377
2.0285
2.0114
2.0033
1.8888
1.8728
1.8647
1.8556
1.8418
1.8327
1.8235
1.8178
1.8075
1.8040
1.8006
1.7914
1.7742
1.6872
1.6815
1.5623
1.5463
1.5325
1.4844
1.4764
1.4718
1.4638
1.4592
1.4524
1.4455
1.4375
1.4295
1.4214
1.4100
1.4020
1.3905
1.3836
1.3779
1.3699
1.3630
1.3516
1.3447
1.3412
1.3355
1.3241
1.3126
1.1660
1.1568
1.1534
1.1282
1.1041
1.0480
1.0331
0.9713
X : parts per Million : Proton

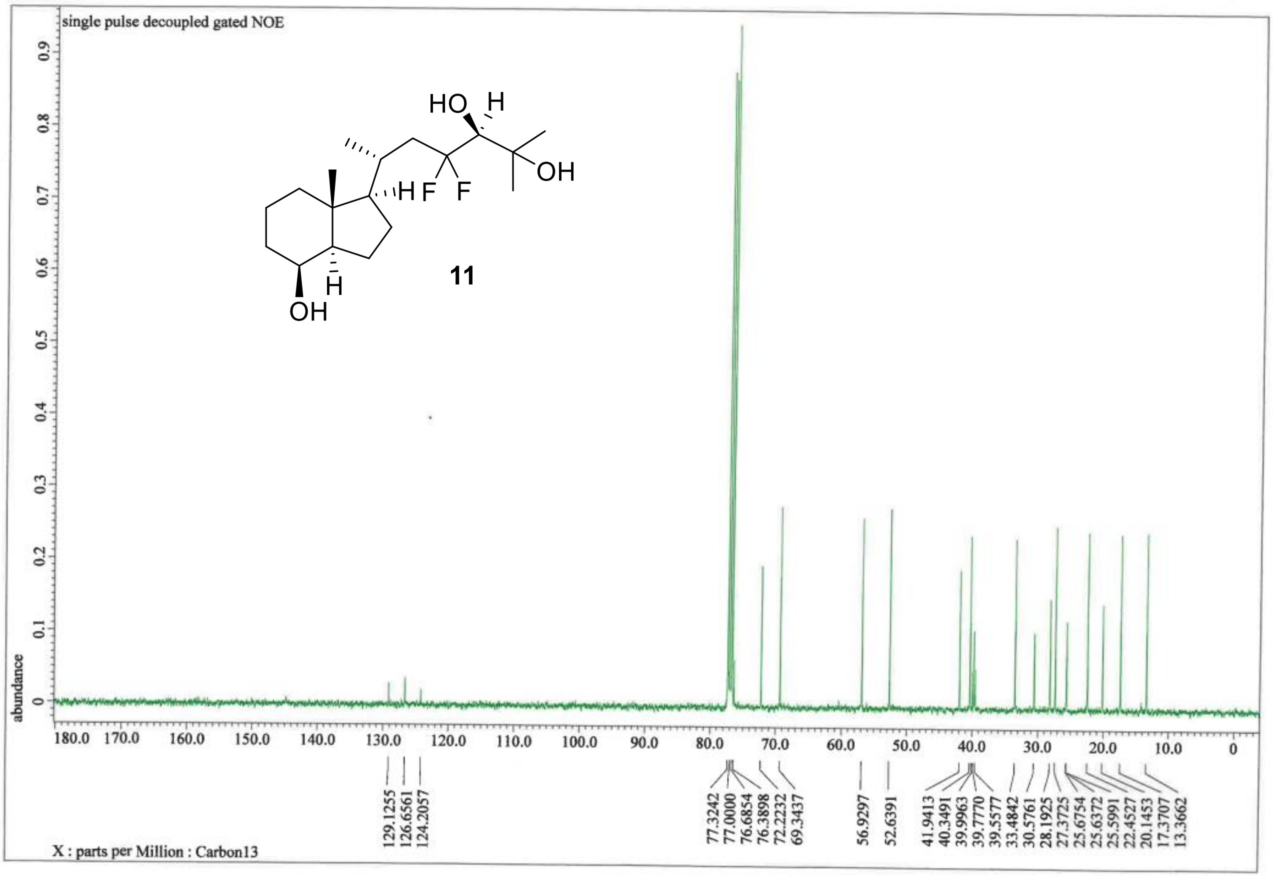
single pulse decoupled gated NOE
HO H
OH
H F F
H
OH
11
abundance
0
0.1
0.2
0.3
0.4
0.5
0.6
0.7
0.8
0.9
180.0
170.0
160.0
150.0
140.0
130.0
120.0
110.0
100.0
90.0
80.0
70.0
60.0
50.0
40.0
30.0
20.0
10.0
0
129.1255
126.6561
124.2057
77.3242
77.0000
76.6854
76.3898
72.2232
69.3437
56.9297
52.6391
41.9413
40.3491
39.9963
39.7770
39.5577
33.4842
30.5761
28.1925
27.3725
25.6754
25.6372
25.5991
22.4527
20.1453
17.3707
13.3662
X : parts per Million : Carbon13

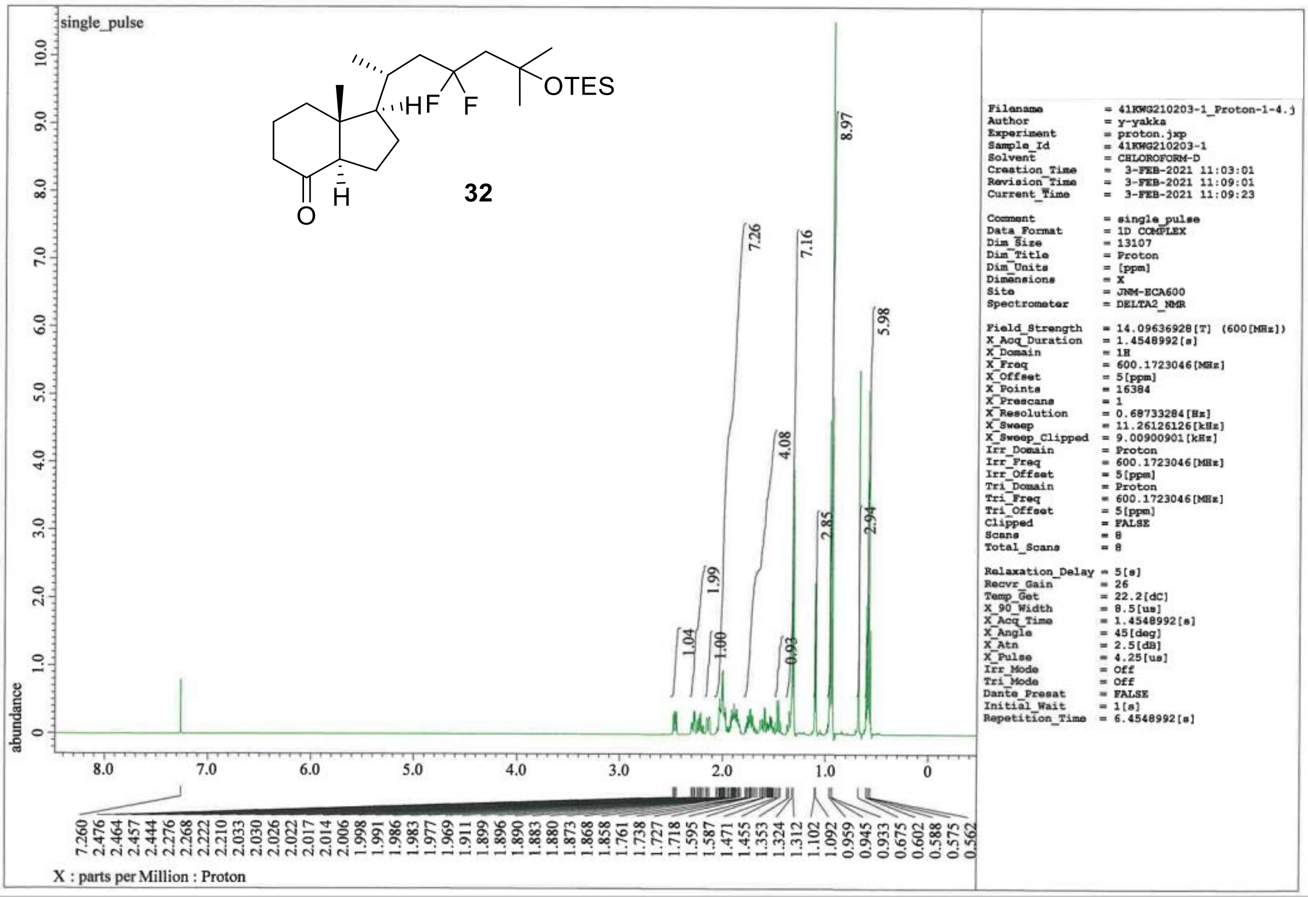
single_pulse
OTES
HF F
H
O
32
abundance
X : parts per Million : Proton
8.0 7.0 6.0 5.0 4.0 3.0 2.0 1.0 0
1.04 1.99 1.00 7.26 0.93 4.08 7.16 2.85 8.97 2.94 5.98
7.260 2.476 2.464 2.457 2.444 2.276 2.268 2.222 2.210 2.033 2.030 2.026 2.022 2.017 2.014 2.006 1.998 1.991 1.986 1.983 1.977 1.969 1.911 1.899 1.896 1.890 1.883 1.880 1.873 1.868 1.858 1.761 1.738 1.727 1.718 1.595 1.587 1.471 1.455 1.353 1.324 1.312 1.102 1.092 0.959 0.945 0.933 0.675 0.602 0.588 0.575 0.562
Filename = 41KWG210203-1_Proton-1-4.j
Author = y-yakka
Experiment = proton.jxp
Sample_Id = 41KWG210203-1
Solvent = CHLOROFORM-D
Creation_Time = 3-FEB-2021 11:03:01
Revision_Time = 3-FEB-2021 11:09:01
Current_Time = 3-FEB-2021 11:09:23
Comment = single_pulse
Data_Format = 1D COMPLEX
Dim_Size = 13107
Dim_Title = Proton
Dim_Units = [ppm]
Dimensions = X
Site = JNM-ECA600
Spectrometer = DELTA2_NMR
Field_Strength = 14.09636928[T] (600[MHz])
X_Acq_Duration = 1.4548992[s]
X_Domain = 1H
X_Freq = 600.1723046[MHz]
X_Offset = 5[ppm]
X_Points = 16384
X_Prescans = 1
X_Resolution = 0.68733284[Hz]
X_Sweep = 11.26126126[kHz]
X_Sweep_Clipped = 9.00900901[kHz]
Irr_Domain = Proton
Irr_Freq = 600.1723046[MHz]
Irr_Offset = 5[ppm]
Tri_Domain = Proton
Tri_Freq = 600.1723046[MHz]
Tri_Offset = 5[ppm]
Clipped = FALSE
Scans = 8
Total_Scans = 8
Relaxation_Delay = 5[s]
Recvr_Gain = 26
Temp_Get = 22.2[dC]
X_90_Width = 8.5[us]
X_Acq_Time = 1.4548992[s]
X_Angle = 45[deg]
X_Atn = 2.5[dB]
X_Pulse = 4.25[us]
Irr_Mode = Off
Tri_Mode = Off
Dante_Presat = FALSE
Initial_Wait = 1[s]
Repetition_Time = 6.4548992[s]

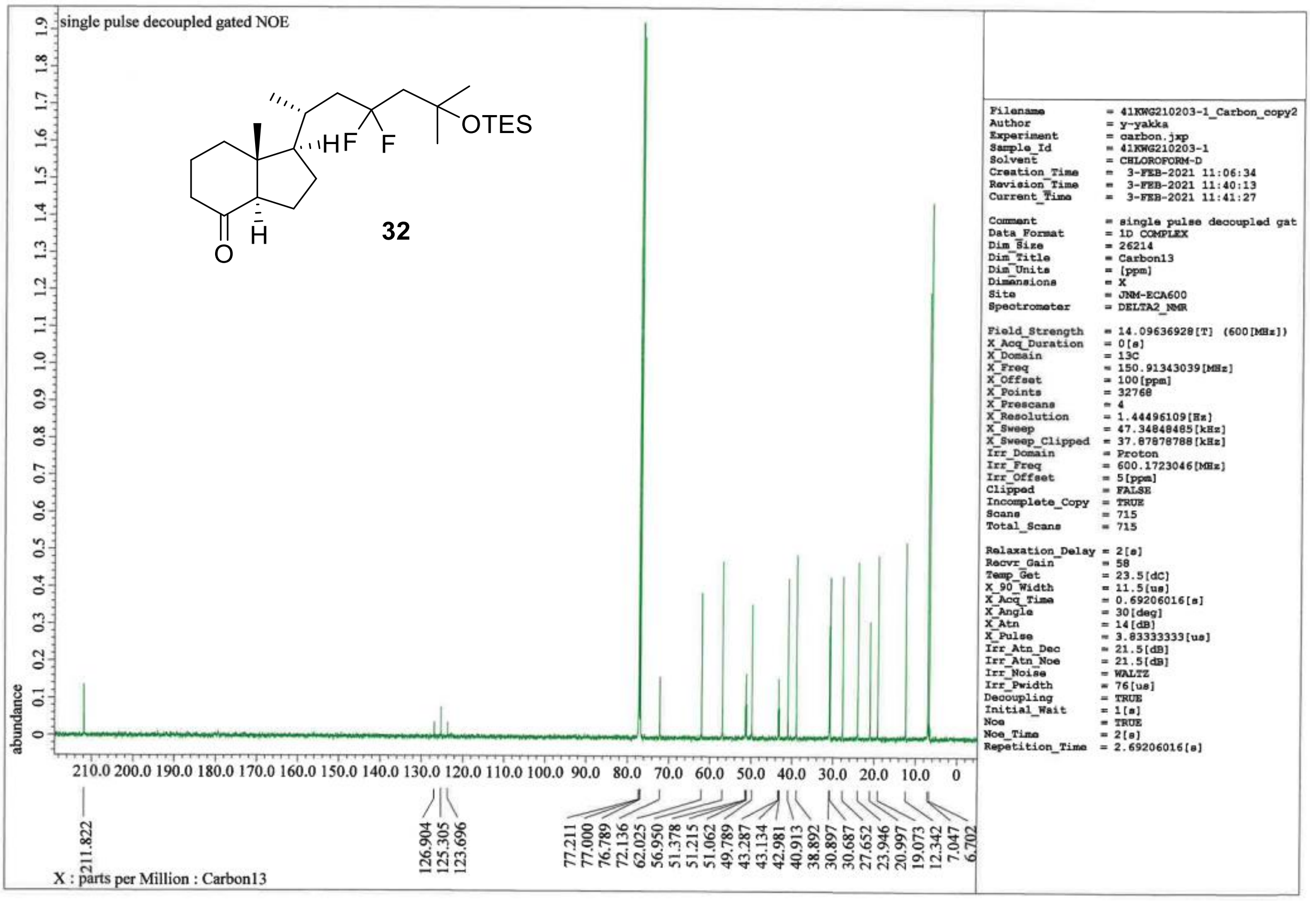
single pulse decoupled gated NOE
OTES
HF F
H
O
32
abundance
X : parts per Million : Carbon13
210.0 200.0 190.0 180.0 170.0 160.0 150.0 140.0 130.0 120.0 110.0 100.0 90.0 80.0 70.0 60.0 50.0 40.0 30.0 20.0 10.0 0
211.822
126.904 125.305 123.696
77.211 77.000 76.789 72.136 62.025 56.950 51.378 51.215 51.062 49.789 43.287 43.134 42.981 40.913 38.892 30.897 30.687 27.652 23.946 20.997 19.073 12.342 7.047 6.702
Filename = 41KWG210203-1_Carbon_copy2
Author = y-yakka
Experiment = carbon.jxp
Sample_Id = 41KWG210203-1
Solvent = CHLOROFORM-D
Creation_Time = 3-FEB-2021 11:06:34
Revision_Time = 3-FEB-2021 11:40:13
Current_Time = 3-FEB-2021 11:41:27
Comment = single pulse decoupled gat
Data_Format = 1D COMPLEX
Dim_Size = 26214
Dim_Title = Carbon13
Dim_Units = [ppm]
Dimensions = X
Site = JNM-ECA600
Spectrometer = DELTA2_NMR
Field_Strength = 14.09636928[T] (600[MHz])
X_Acq_Duration = 0[s]
X_Domain = 13C
X_Freq = 150.91343039[MHz]
X_Offset = 100[ppm]
X_Points = 32768
X_Prescans = 4
X_Resolution = 1.44496109[Hz]
X_Sweep = 47.34848485[kHz]
X_Sweep_Clipped = 37.87878788[kHz]
Irr_Domain = Proton
Irr_Freq = 600.1723046[MHz]
Irr_Offset = 5[ppm]
Clipped = FALSE
Incomplete_Copy = TRUE
Scans = 715
Total_Scans = 715
Relaxation_Delay = 2[s]
Recvr_Gain = 58
Temp_Get = 23.5[dC]
X_90_Width = 11.5[us]
X_Acq_Time = 0.69206016[s]
X_Angle = 30[deg]
X_Atn = 14[dB]
X_Pulse = 3.83333333[us]
Irr_Atn_Dec = 21.5[dB]
Irr_Atn_Noe = 21.5[dB]
Irr_Noise = WALTZ
Irr_Pwidth = 76[us]
Decoupling = TRUE
Initial_Wait = 1[s]
Noe = TRUE
Noe_Time = 2[s]
Repetition_Time = 2.69206016[s]

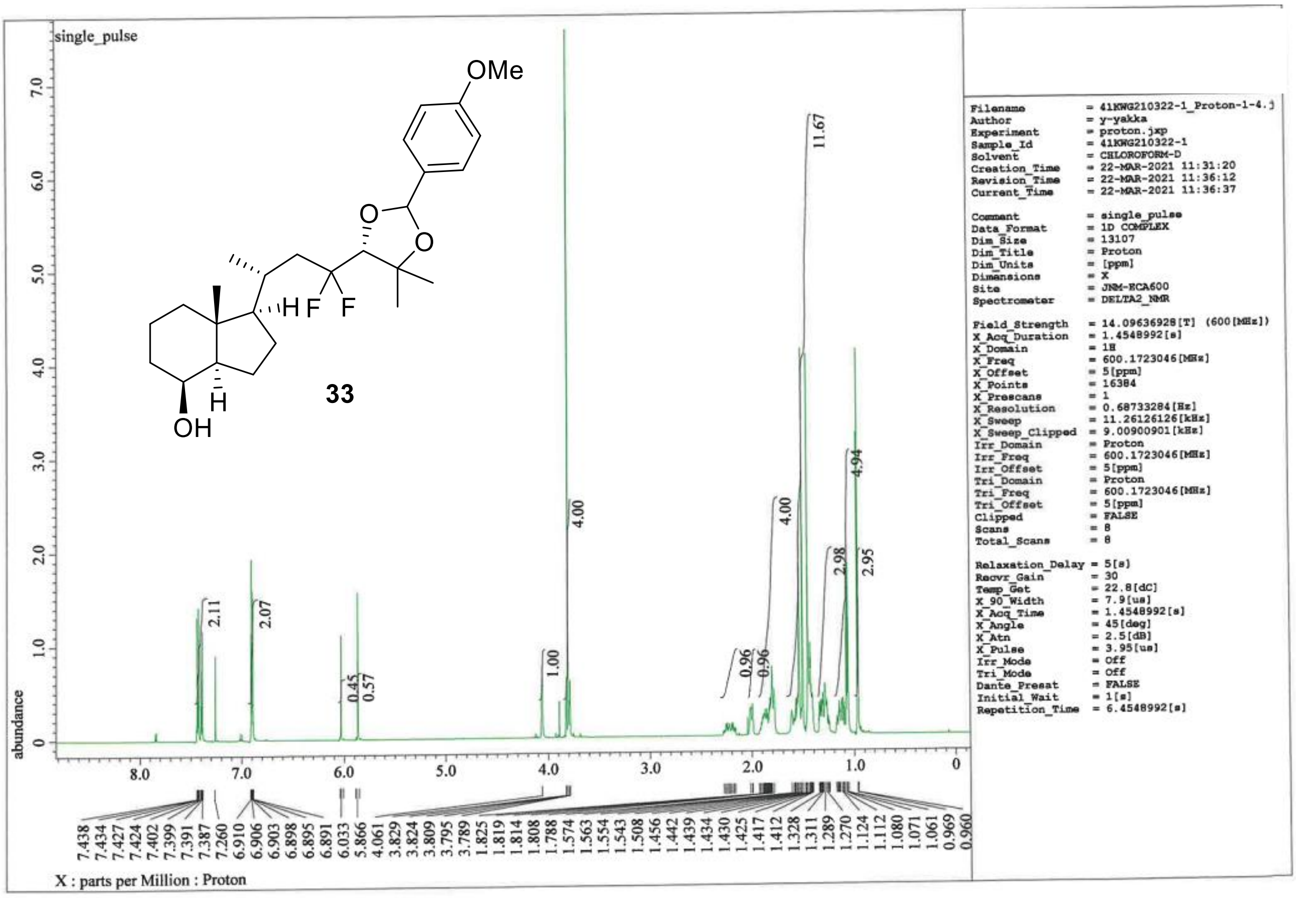
single_pulse
OMe
O
O
H F F
H
OH
33
abundance
X : parts per Million : Proton
2.11
2.07
0.45
0.57
1.00
4.00
0.96
0.96
4.00
11.67
2.98
4.94
2.95
7.438 7.434 7.427 7.424 7.402 7.399 7.391 7.387 7.260 6.910 6.906 6.903 6.898 6.895 6.891 6.033 5.866 4.061 3.829 3.824 3.809 3.795 3.789 1.825 1.819 1.814 1.808 1.788 1.574 1.563 1.554 1.543 1.508 1.456 1.442 1.439 1.434 1.430 1.425 1.417 1.412 1.328 1.311 1.289 1.270 1.124 1.080 1.071 1.061 0.969 0.960
Filename = 41KWG210322-1_Proton-1-4.j
Author = y-yakka
Experiment = proton.jxp
Sample_Id = 41KWG210322-1
Solvent = CHLOROFORM-D
Creation_Time = 22-MAR-2021 11:31:20
Revision_Time = 22-MAR-2021 11:36:12
Current_Time = 22-MAR-2021 11:36:37
Comment = single_pulse
Data_Format = 1D COMPLEX
Dim_Size = 13107
Dim_Title = Proton
Dim_Units = [ppm]
Dimensions = X
Site = JNM-ECA600
Spectrometer = DELTA2_NMR
Field_Strength = 14.09636928[T] (600[MHz])
X_Acq_Duration = 1.4548992[s]
X_Domain = 1H
X_Freq = 600.1723046[MHz]
X_Offset = 5[ppm]
X_Points = 16384
X_Prescans = 1
X_Resolution = 0.68733284[Hz]
X_Sweep = 11.26126126[kHz]
X_Sweep_Clipped = 9.00900901[kHz]
Irr_Domain = Proton
Irr_Freq = 600.1723046[MHz]
Irr_Offset = 5[ppm]
Tri_Domain = Proton
Tri_Freq = 600.1723046[MHz]
Tri_Offset = 5[ppm]
Clipped = FALSE
Scans = 8
Total_Scans = 8
Relaxation_Delay = 5[s]
Recvr_Gain = 30
Temp_Get = 22.8[dC]
X_90_Width = 7.9[us]
X_Acq_Time = 1.4548992[s]
X_Angle = 45[deg]
X_Atn = 2.5[dB]
X_Pulse = 3.95[us]
Irr_Mode = Off
Tri_Mode = Off
Dante_Presat = FALSE
Initial_Wait = 1[s]
Repetition_Time = 6.4548992[s]

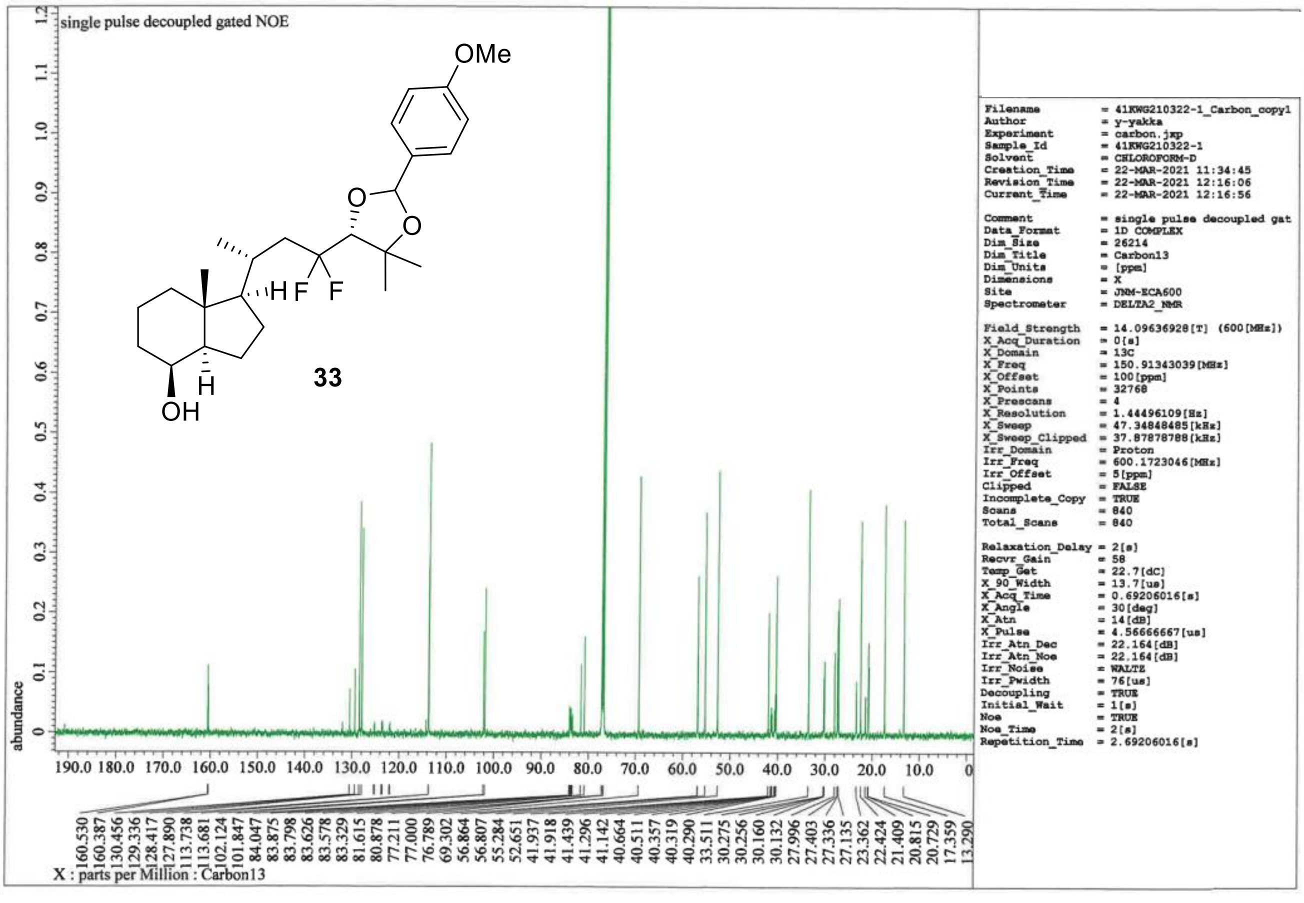
single pulse decoupled gated NOE
OMe
O
O
H F F
H
OH
33
abundance
X : parts per Million : Carbon13
160.530 160.387 130.456 129.336 128.417 127.890 113.738 113.681 102.124 101.847 84.047 83.875 83.798 83.626 83.578 83.329 81.615 80.878 77.211 77.000 76.789 69.302 56.864 56.807 55.284 52.651 41.937 41.918 41.439 41.296 41.142 40.664 40.511 40.357 40.319 40.290 33.511 30.275 30.256 30.160 30.132 27.996 27.403 27.336 27.135 23.362 22.424 21.409 20.815 20.729 17.359 13.290
Filename = 41KWG210322-1_Carbon_copy1
Author = y-yakka
Experiment = carbon.jxp
Sample_Id = 41KWG210322-1
Solvent = CHLOROFORM-D
Creation_Time = 22-MAR-2021 11:34:45
Revision_Time = 22-MAR-2021 12:16:06
Current_Time = 22-MAR-2021 12:16:56
Comment = single pulse decoupled gat
Data_Format = 1D COMPLEX
Dim_Size = 26214
Dim_Title = Carbon13
Dim_Units = [ppm]
Dimensions = X
Site = JNM-ECA600
Spectrometer = DELTA2_NMR
Field_Strength = 14.09636928[T] (600[MHz])
X_Acq_Duration = 0[s]
X_Domain = 13C
X_Freq = 150.91343039[MHz]
X_Offset = 100[ppm]
X_Points = 32768
X_Prescans = 4
X_Resolution = 1.44496109[Hz]
X_Sweep = 47.34848485[kHz]
X_Sweep_Clipped = 37.87878788[kHz]
Irr_Domain = Proton
Irr_Freq = 600.1723046[MHz]
Irr_Offset = 5[ppm]
Clipped = FALSE
Incomplete_Copy = TRUE
Scans = 840
Total_Scans = 840
Relaxation_Delay = 2[s]
Recvr_Gain = 58
Temp_Get = 22.7[dC]
X_90_Width = 13.7[us]
X_Acq_Time = 0.69206016[s]
X_Angle = 30[deg]
X_Atn = 14[dB]
X_Pulse = 4.56666667[us]
Irr_Atn_Dec = 22.164[dB]
Irr_Atn_Noe = 22.164[dB]
Irr_Noise = WALTZ
Irr_Pwidth = 76[us]
Decoupling = TRUE
Initial_Wait = 1[s]
Noe = TRUE
Noe_Time = 2[s]
Repetition_Time = 2.69206016[s]

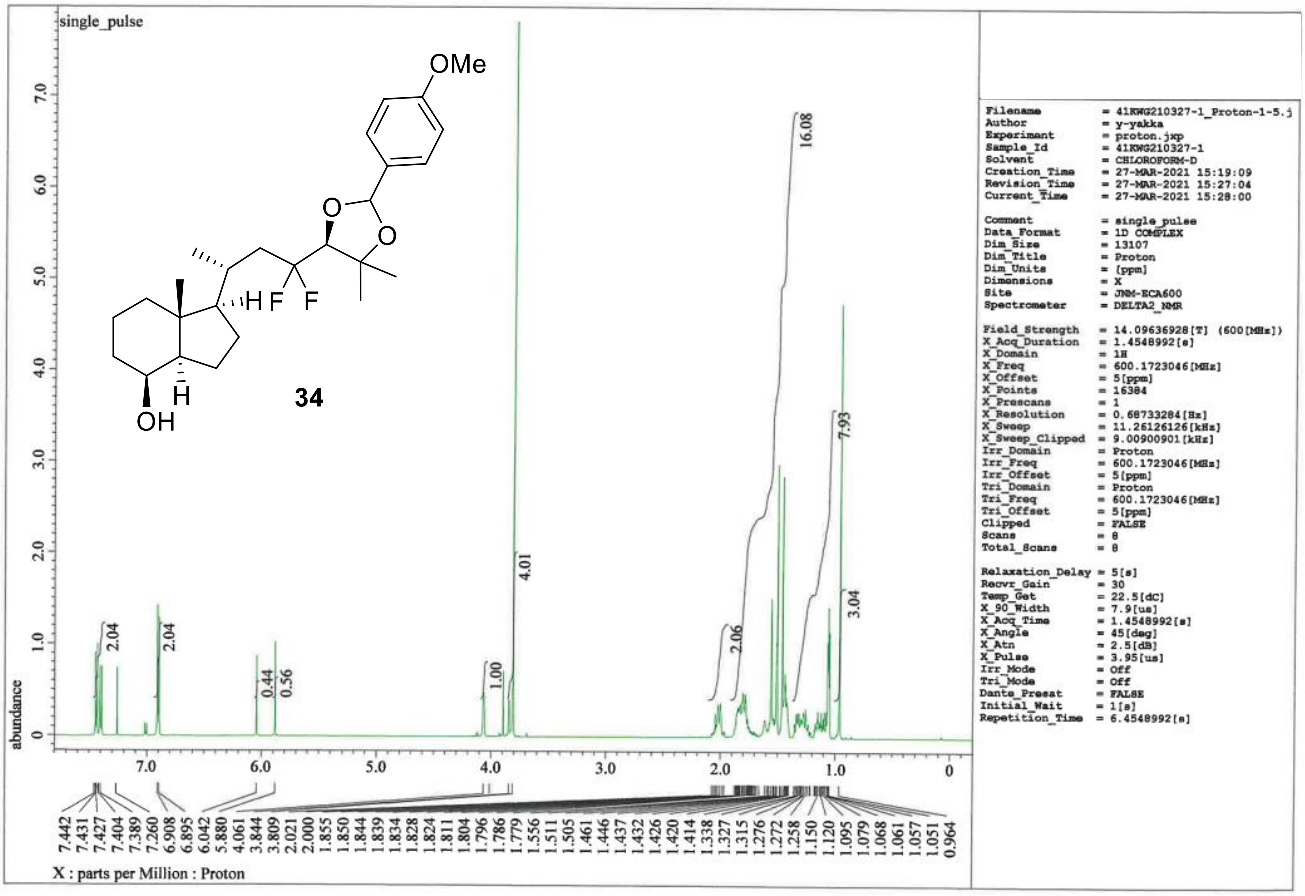
single_pulse
OMe
O
O
H F F
H
OH
34
16.08
7.93
4.01
3.04
2.06
2.04
2.04
1.00
0.44
0.56
abundance
7.442
7.431
7.427
7.404
7.389
7.260
6.908
6.895
6.042
5.880
4.061
3.844
3.809
2.021
2.000
1.855
1.850
1.844
1.839
1.834
1.828
1.824
1.811
1.804
1.796
1.786
1.779
1.556
1.511
1.505
1.461
1.446
1.437
1.432
1.426
1.420
1.414
1.338
1.327
1.315
1.276
1.272
1.258
1.150
1.120
1.095
1.079
1.068
1.061
1.057
1.051
0.964
X : parts per Million : Proton
Filename = 41KWG210327-1_Proton-1-5.j
Author = y-yakka
Experiment = proton.jxp
Sample_Id = 41KWG210327-1
Solvent = CHLOROFORM-D
Creation_Time = 27-MAR-2021 15:19:09
Revision_Time = 27-MAR-2021 15:27:04
Current_Time = 27-MAR-2021 15:28:00
Comment = single_pulse
Data_Format = 1D COMPLEX
Dim_Size = 13107
Dim_Title = Proton
Dim_Units = [ppm]
Dimensions = X
Site = JNM-ECA600
Spectrometer = DELTA2_NMR
Field_Strength = 14.09636928[T] (600[MHz])
X_Acq_Duration = 1.4548992[s]
X_Domain = 1H
X_Freq = 600.1723046[MHz]
X_Offset = 5[ppm]
X_Points = 16384
X_Prescans = 1
X_Resolution = 0.68733284[Hz]
X_Sweep = 11.26126126[kHz]
X_Sweep_Clipped = 9.00900901[kHz]
Irr_Domain = Proton
Irr_Freq = 600.1723046[MHz]
Irr_Offset = 5[ppm]
Tri_Domain = Proton
Tri_Freq = 600.1723046[MHz]
Tri_Offset = 5[ppm]
Clipped = FALSE
Scans = 8
Total_Scans = 8
Relaxation_Delay = 5[s]
Recvr_Gain = 30
Temp_Get = 22.5[dC]
X_90_Width = 7.9[us]
X_Acq_Time = 1.4548992[s]
X_Angle = 45[deg]
X_Atn = 2.5[dB]
X_Pulse = 3.95[us]
Irr_Mode = Off
Tri_Mode = Off
Dante_Presat = FALSE
Initial_Wait = 1[s]
Repetition_Time = 6.4548992[s]

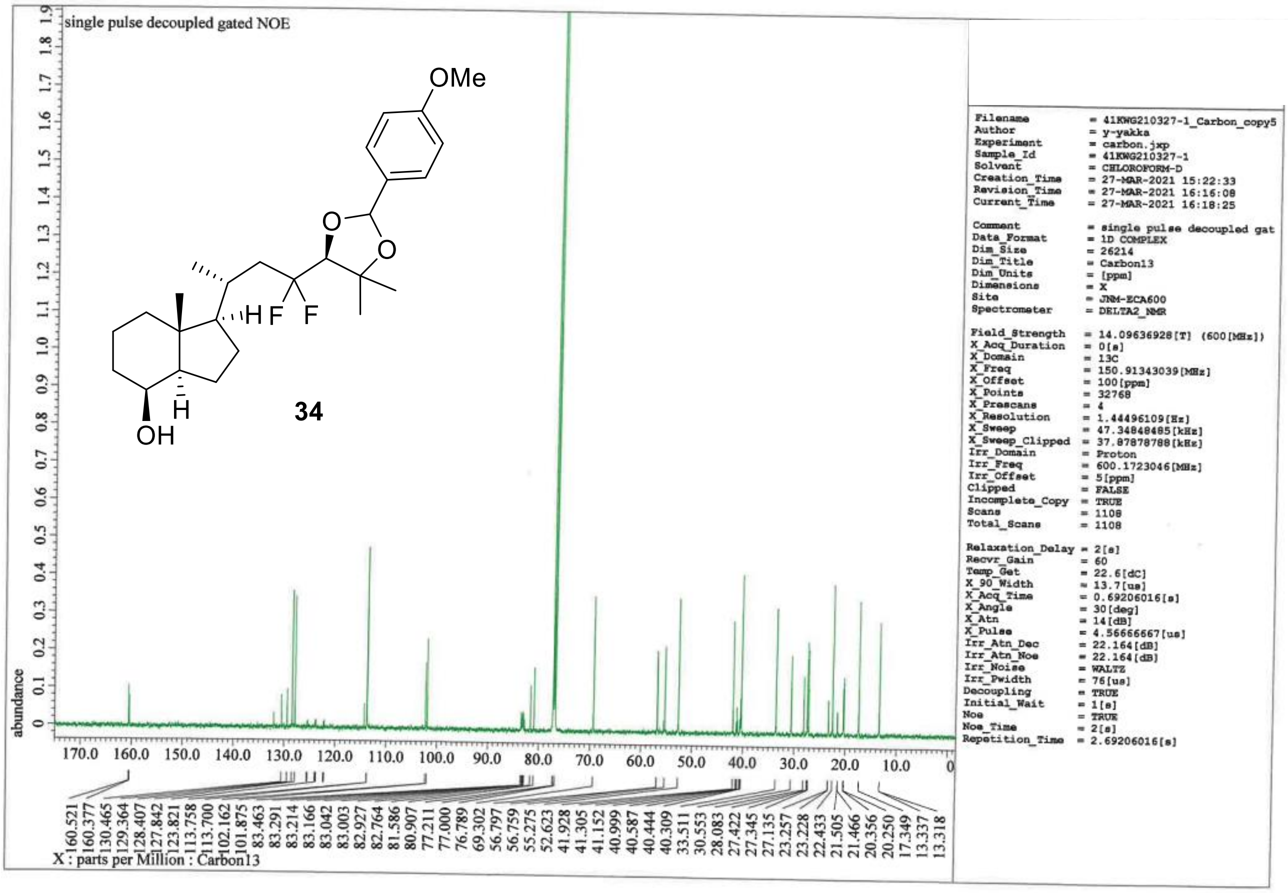
single pulse decoupled gated NOE
OMe
O
O
H F F
H
OH
34
abundance
160.521
160.377
130.465
129.364
128.407
127.842
123.821
113.758
113.700
102.162
101.875
83.463
83.291
83.214
83.166
83.042
83.003
82.927
82.764
81.586
80.907
77.211
77.000
76.789
69.302
56.797
56.759
55.275
52.623
41.928
41.305
41.152
40.999
40.587
40.444
40.309
33.511
30.553
28.083
27.422
27.345
27.135
23.257
23.228
22.433
21.505
21.466
20.356
20.250
17.349
13.337
13.318
X : parts per Million : Carbon13
Filename = 41KWG210327-1_Carbon_copy5
Author = y-yakka
Experiment = carbon.jxp
Sample_Id = 41KWG210327-1
Solvent = CHLOROFORM-D
Creation_Time = 27-MAR-2021 15:22:33
Revision_Time = 27-MAR-2021 16:16:08
Current_Time = 27-MAR-2021 16:18:25
Comment = single pulse decoupled gat
Data_Format = 1D COMPLEX
Dim_Size = 26214
Dim_Title = Carbon13
Dim_Units = [ppm]
Dimensions = X
Site = JNM-ECA600
Spectrometer = DELTA2_NMR
Field_Strength = 14.09636928[T] (600[MHz])
X_Acq_Duration = 0[s]
X_Domain = 13C
X_Freq = 150.91343039[MHz]
X_Offset = 100[ppm]
X_Points = 32768
X_Prescans = 4
X_Resolution = 1.44496109[Hz]
X_Sweep = 47.34848485[kHz]
X_Sweep_Clipped = 37.87878788[kHz]
Irr_Domain = Proton
Irr_Freq = 600.1723046[MHz]
Irr_Offset = 5[ppm]
Clipped = FALSE
Incomplete_Copy = TRUE
Scans = 1108
Total_Scans = 1108
Relaxation_Delay = 2[s]
Recvr_Gain = 60
Temp_Get = 22.6[dC]
X_90_Width = 13.7[us]
X_Acq_Time = 0.69206016[s]
X_Angle = 30[deg]
X_Atn = 14[dB]
X_Pulse = 4.56666667[us]
Irr_Atn_Dec = 22.164[dB]
Irr_Atn_Noe = 22.164[dB]
Irr_Noise = WALTZ
Irr_Pwidth = 76[us]
Decoupling = TRUE
Initial_Wait = 1[s]
Noe = TRUE
Noe_Time = 2[s]
Repetition_Time = 2.69206016[s]

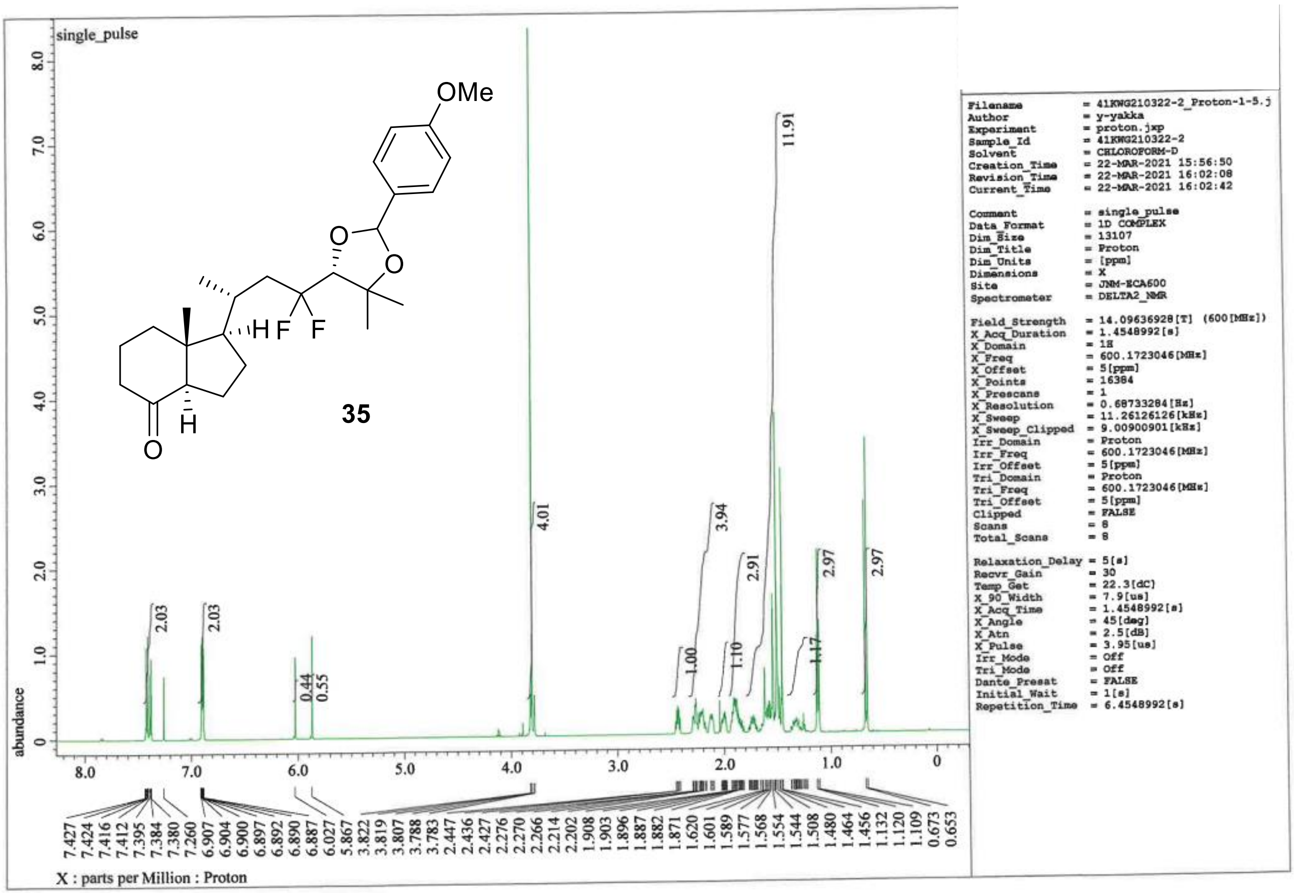
single_pulse
OMe
35
abundance
X : parts per Million : Proton

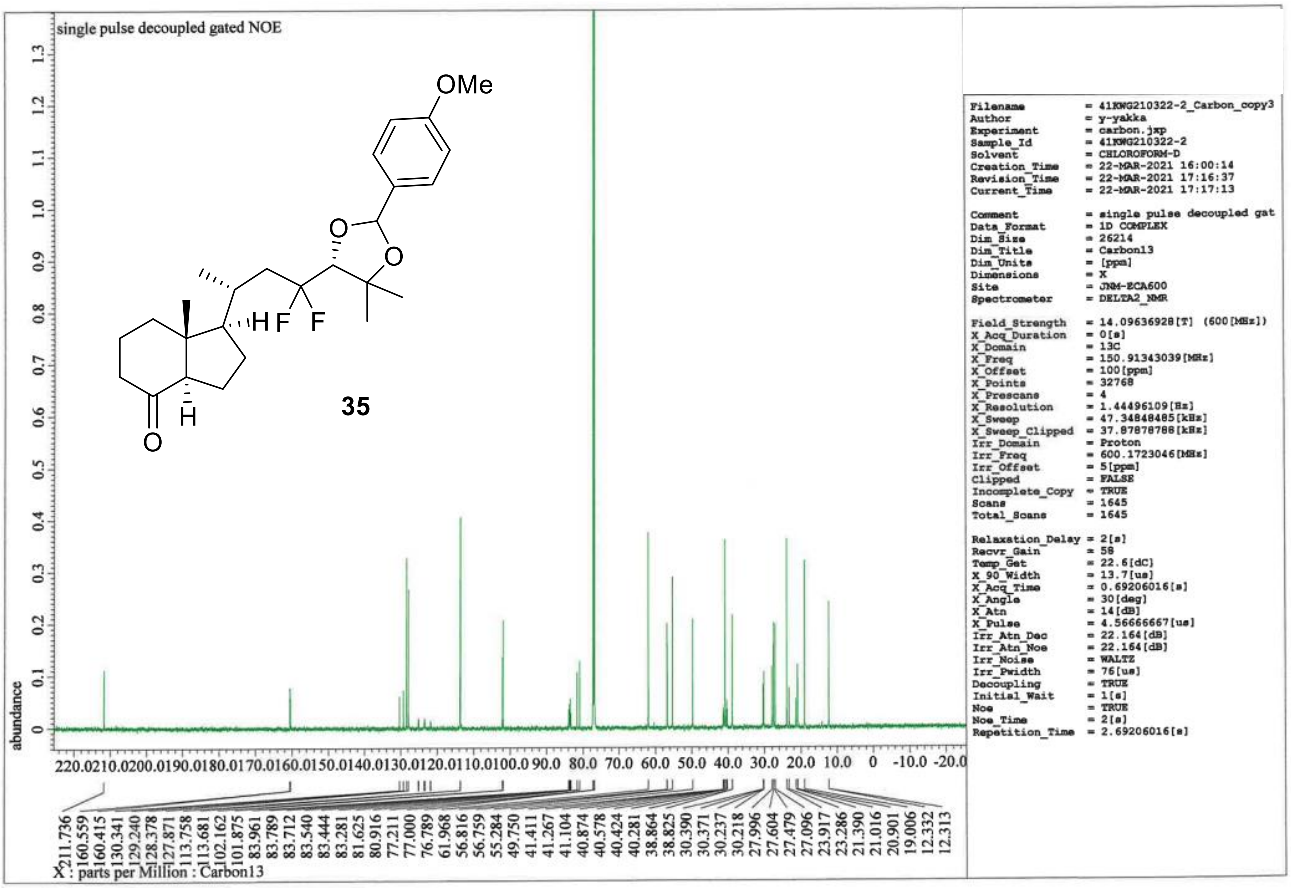
single pulse decoupled gated NOE
OMe
35
abundance
X : parts per Million : Carbon13

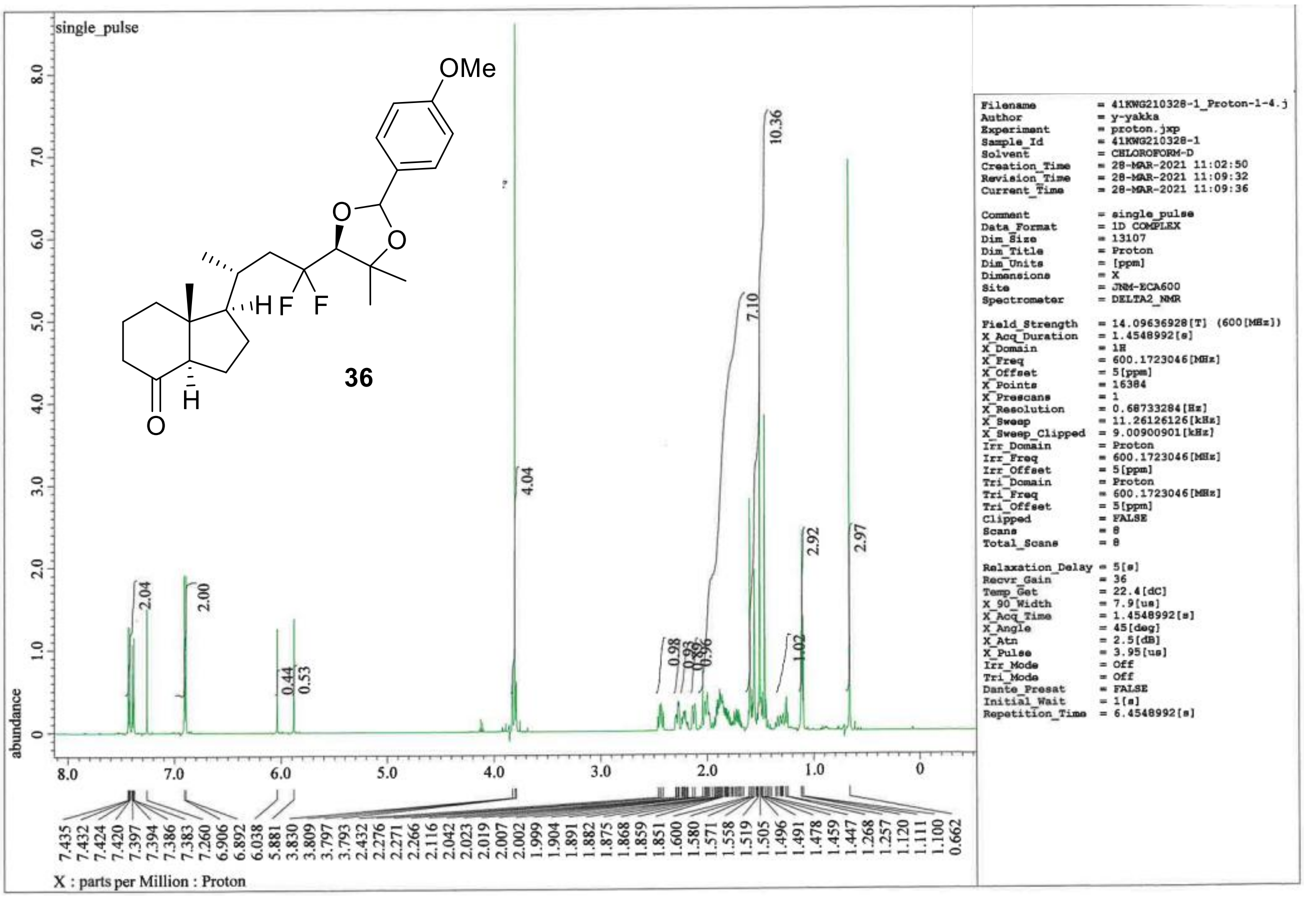
single_pulse
OMe
H F F
H
O
36
abundance
X : parts per Million : Proton
Filename = 41KWG210328-1_Proton-1-4.j
Author = y-yakka
Experiment = proton.jxp
Sample_Id = 41KWG210328-1
Solvent = CHLOROFORM-D
Creation_Time = 28-MAR-2021 11:02:50
Revision_Time = 28-MAR-2021 11:09:32
Current_Time = 28-MAR-2021 11:09:36
Comment = single_pulse
Data_Format = 1D COMPLEX
Dim_Size = 13107
Dim_Title = Proton
Dim_Units = [ppm]
Dimensions = X
Site = JNM-ECA600
Spectrometer = DELTA2_NMR
Field_Strength = 14.09636928[T] (600[MHz])
X_Acq_Duration = 1.4548992[s]
X_Domain = 1H
X_Freq = 600.1723046[MHz]
X_Offset = 5[ppm]
X_Points = 16384
X_Prescans = 1
X_Resolution = 0.68733284[Hz]
X_Sweep = 11.26126126[kHz]
X_Sweep_Clipped = 9.00900901[kHz]
Irr_Domain = Proton
Irr_Freq = 600.1723046[MHz]
Irr_Offset = 5[ppm]
Tri_Domain = Proton
Tri_Freq = 600.1723046[MHz]
Tri_Offset = 5[ppm]
Clipped = FALSE
Scans = 8
Total_Scans = 8
Relaxation_Delay = 5[s]
Recvr_Gain = 36
Temp_Get = 22.4[dC]
X_90_Width = 7.9[us]
X_Acq_Time = 1.4548992[s]
X_Angle = 45[deg]
X_Atn = 2.5[dB]
X_Pulse = 3.95[us]
Irr_Mode = Off
Tri_Mode = Off
Dante_Presat = FALSE
Initial_Wait = 1[s]
Repetition_Time = 6.4548992[s]

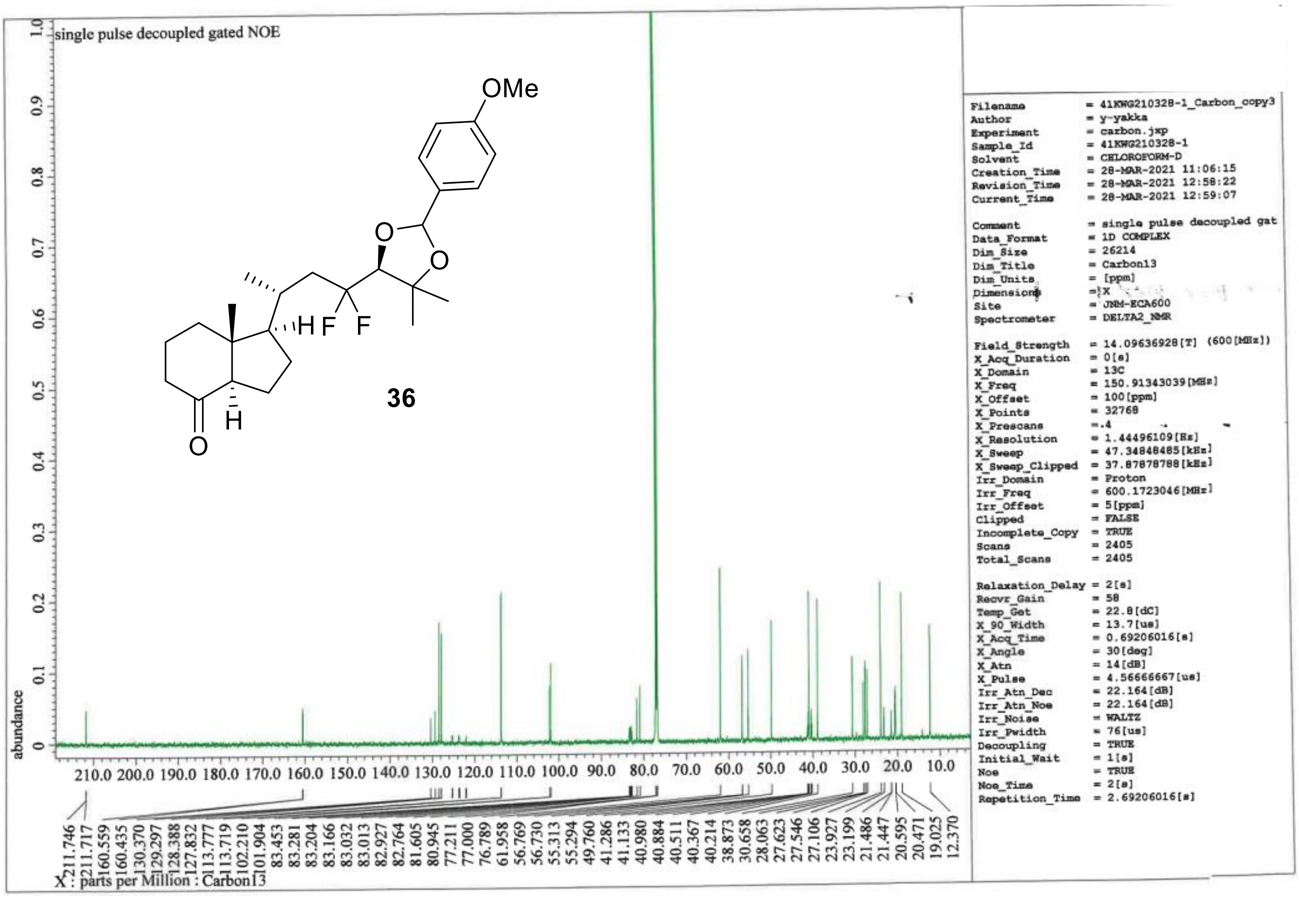
single pulse decoupled gated NOE
OMe
H F F
H
O
36
abundance
X : parts per Million : Carbon13
Filename = 41KWG210328-1_Carbon_copy3
Author = y-yakka
Experiment = carbon.jxp
Sample_Id = 41KWG210328-1
Solvent = CHLOROFORM-D
Creation_Time = 28-MAR-2021 11:06:15
Revision_Time = 28-MAR-2021 12:58:22
Current_Time = 28-MAR-2021 12:59:07
Comment = single pulse decoupled gat
Data_Format = 1D COMPLEX
Dim_Size = 26214
Dim_Title = Carbon13
Dim_Units = [ppm]
Site = JNM-ECA600
Spectrometer = DELTA2_NMR
Field_Strength = 14.09636928[T] (600[MHz])
X_Domain = 13C
X_Freq = 150.91343039[MHz]
X_Offset = 100[ppm]
X_Points = 32768
X_Resolution = 1.44496109[Hz]
X_Sweep = 47.34848485[kHz]
X_Sweep_Clipped = 37.87878788[kHz]
Irr_Domain = Proton
Irr_Freq = 600.1723046[MHz]
Irr_Offset = 5[ppm]
Clipped = FALSE
Incomplete_Copy = TRUE
Scans = 2405
Total_Scans = 2405
Relaxation_Delay = 2[s]
Recvr_Gain = 58
Temp_Get = 22.8[dC]
X_90_Width = 13.7[us]
X_Acq_Time = 0.69206016[s]
X_Angle = 30[deg]
X_Atn = 14[dB]
X_Pulse = 4.56666667[us]
Irr_Atn_Dec = 22.164[dB]
Irr_Atn_Noe = 22.164[dB]
Irr_Noise = WALTZ
Irr_Pwidth = 76[us]
Decoupling = TRUE
Initial_Wait = 1[s]
Noe = TRUE
Noe_Time = 2[s]
Repetition_Time = 2.69206016[s]

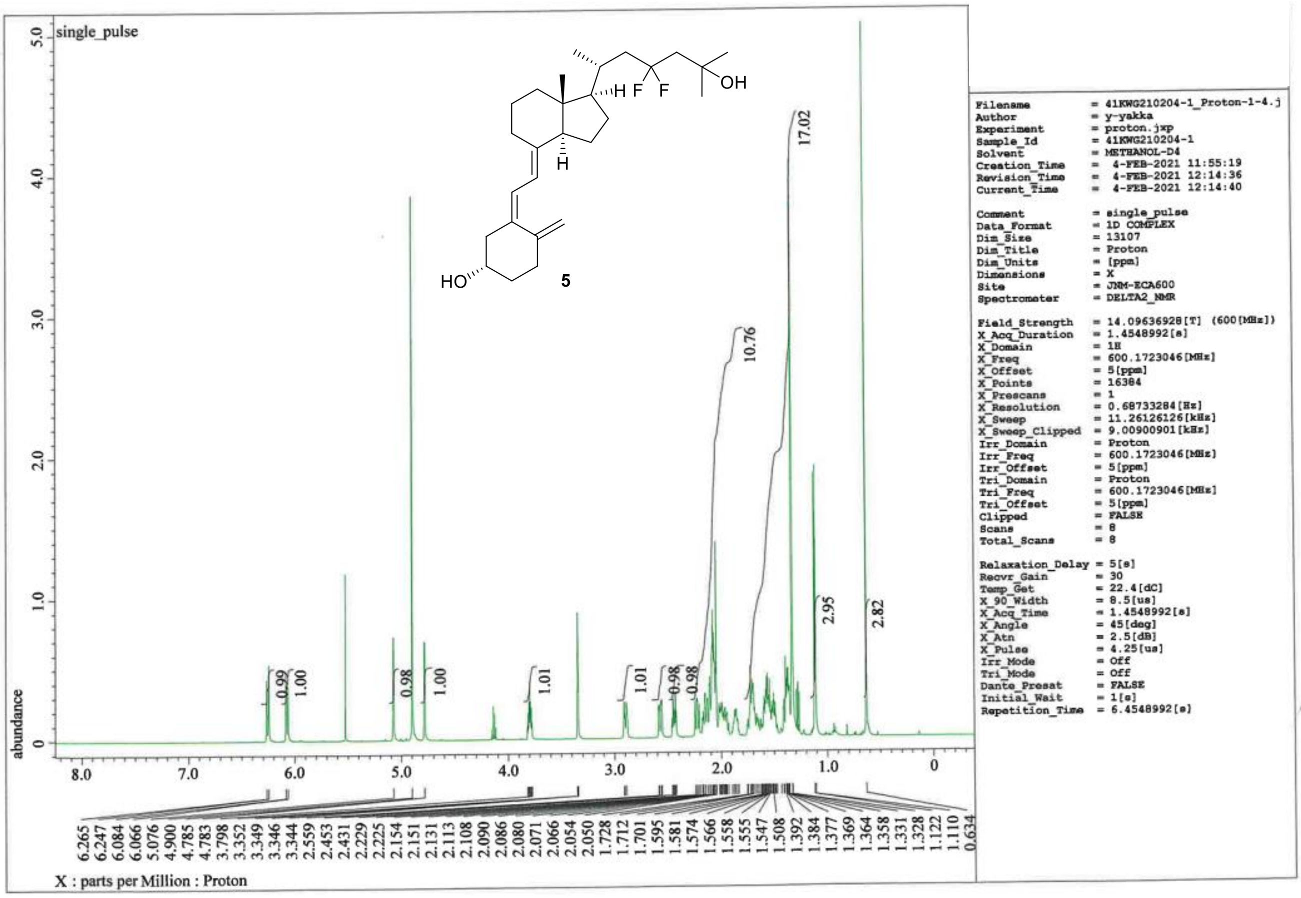
single_pulse
abundance
X : parts per Million : Proton
HO
5
H F F
OH
H
0.99
1.00
0.98
1.00
1.01
1.01
0.98
0.98
10.76
17.02
2.95
2.82
6.265
6.247
6.084
6.066
5.076
4.900
4.785
4.783
3.798
3.352
3.349
3.346
3.344
2.559
2.453
2.431
2.229
2.225
2.154
2.151
2.131
2.113
2.108
2.090
2.086
2.080
2.071
2.066
2.054
2.050
1.728
1.712
1.701
1.595
1.581
1.574
1.566
1.558
1.555
1.547
1.508
1.392
1.384
1.377
1.369
1.364
1.358
1.331
1.328
1.122
1.110
0.634
Filename = 41KWG210204-1_Proton-1-4.j
Author = y-yakka
Experiment = proton.jxp
Sample_Id = 41KWG210204-1
Solvent = METHANOL-D4
Creation_Time = 4-FEB-2021 11:55:19
Revision_Time = 4-FEB-2021 12:14:36
Current_Time = 4-FEB-2021 12:14:40
Comment = single_pulse
Data_Format = 1D COMPLEX
Dim_Size = 13107
Dim_Title = Proton
Dim_Units = [ppm]
Dimensions = X
Site = JNM-ECA600
Spectrometer = DELTA2_NMR
Field_Strength = 14.09636928[T] (600[MHz])
X_Acq_Duration = 1.4548992[s]
X_Domain = 1H
X_Freq = 600.1723046[MHz]
X_Offset = 5[ppm]
X_Points = 16384
X_Prescans = 1
X_Resolution = 0.68733284[Hz]
X_Sweep = 11.26126126[kHz]
X_Sweep_Clipped = 9.00900901[kHz]
Irr_Domain = Proton
Irr_Freq = 600.1723046[MHz]
Irr_Offset = 5[ppm]
Tri_Domain = Proton
Tri_Freq = 600.1723046[MHz]
Tri_Offset = 5[ppm]
Clipped = FALSE
Scans = 8
Total_Scans = 8
Relaxation_Delay = 5[s]
Recvr_Gain = 30
Temp_Get = 22.4[dC]
X_90_Width = 8.5[us]
X_Acq_Time = 1.4548992[s]
X_Angle = 45[deg]
X_Atn = 2.5[dB]
X_Pulse = 4.25[us]
Irr_Mode = Off
Tri_Mode = Off
Dante_Presat = FALSE
Initial_Wait = 1[s]
Repetition_Time = 6.4548992[s]

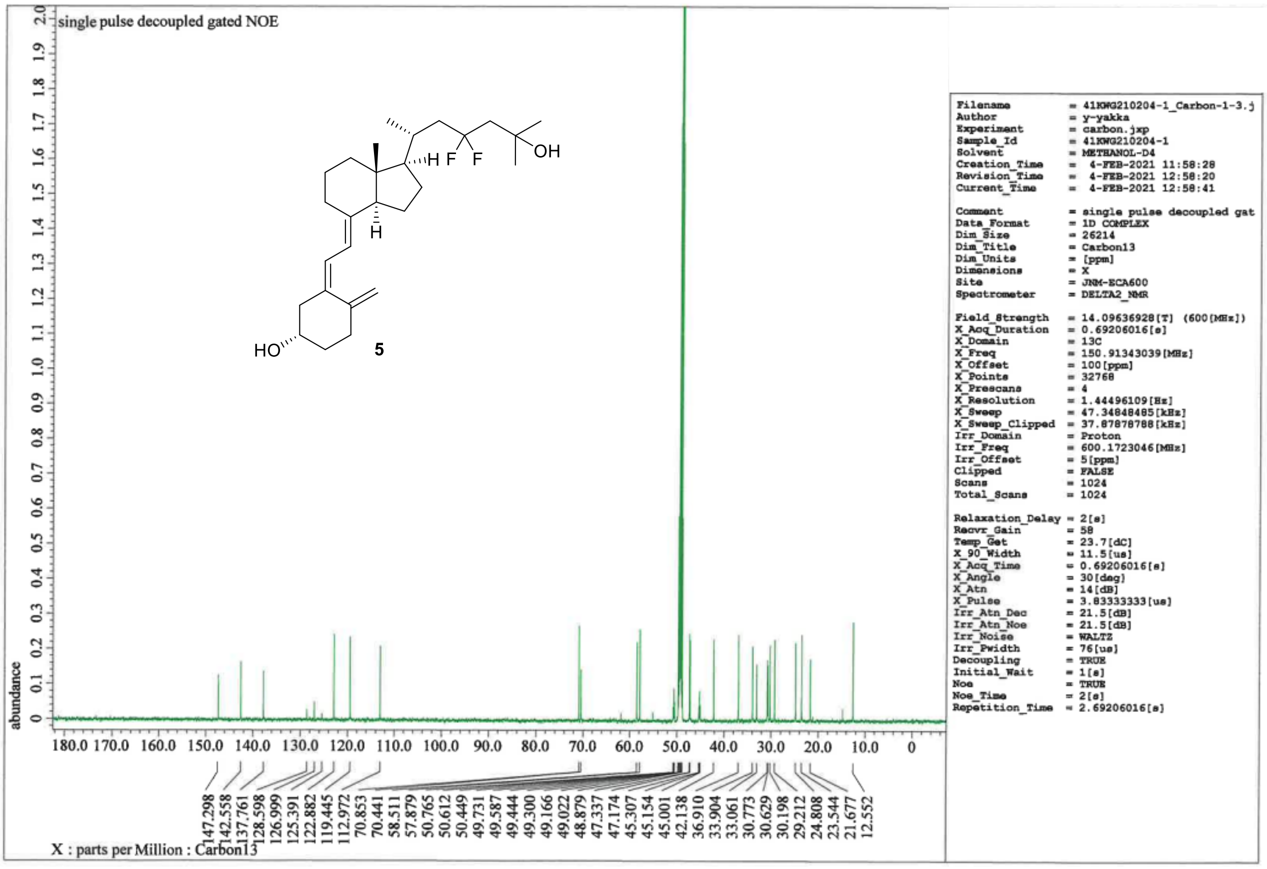
single pulse decoupled gated NOE
abundance
X : parts per Million : Carbon13
HO
5
H F F
OH
H
147.298
142.558
137.761
128.598
126.999
125.391
122.882
119.445
112.972
70.853
70.441
58.511
57.879
50.765
50.612
50.449
49.731
49.587
49.444
49.300
49.166
49.022
48.879
47.337
47.174
45.307
45.154
45.001
42.138
36.910
33.904
33.061
30.773
30.629
30.198
29.212
24.808
23.544
21.677
12.52
Filename = 41KWG210204-1_Carbon-1-3.j
Author = y-yakka
Experiment = carbon.jxp
Sample_Id = 41KWG210204-1
Solvent = METHANOL-D4
Creation_Time = 4-FEB-2021 11:58:28
Revision_Time = 4-FEB-2021 12:58:20
Current_Time = 4-FEB-2021 12:58:41
Comment = single pulse decoupled gat
Data_Format = 1D COMPLEX
Dim_Size = 26214
Dim_Title = Carbon13
Dim_Units = [ppm]
Dimensions = X
Site = JNM-ECA600
Spectrometer = DELTA2_NMR
Field_Strength = 14.09636928[T] (600[MHz])
X_Acq_Duration = 0.69206016[s]
X_Domain = 13C
X_Freq = 150.91343039[MHz]
X_Offset = 100[ppm]
X_Points = 32768
X_Prescans = 4
X_Resolution = 1.44496109[Hz]
X_Sweep = 47.34848485[kHz]
X_Sweep_Clipped = 37.87878788[kHz]
Irr_Domain = Proton
Irr_Freq = 600.1723046[MHz]
Irr_Offset = 5[ppm]
Clipped = FALSE
Scans = 1024
Total_Scans = 1024
Relaxation_Delay = 2[s]
Recvr_Gain = 58
Temp_Get = 23.7[dC]
X_90_Width = 11.5[us]
X_Acq_Time = 0.69206016[s]
X_Angle = 30[deg]
X_Atn = 14[dB]
X_Pulse = 3.83333333[us]
Irr_Atn_Dec = 21.5[dB]
Irr_Atn_Noe = 21.5[dB]
Irr_Noise = WALTZ
Irr_Pwidth = 76[us]
Decoupling = TRUE
Initial_Wait = 1[s]
Noe = TRUE
Noe_Time = 2[s]
Repetition_Time = 2.69206016[s]

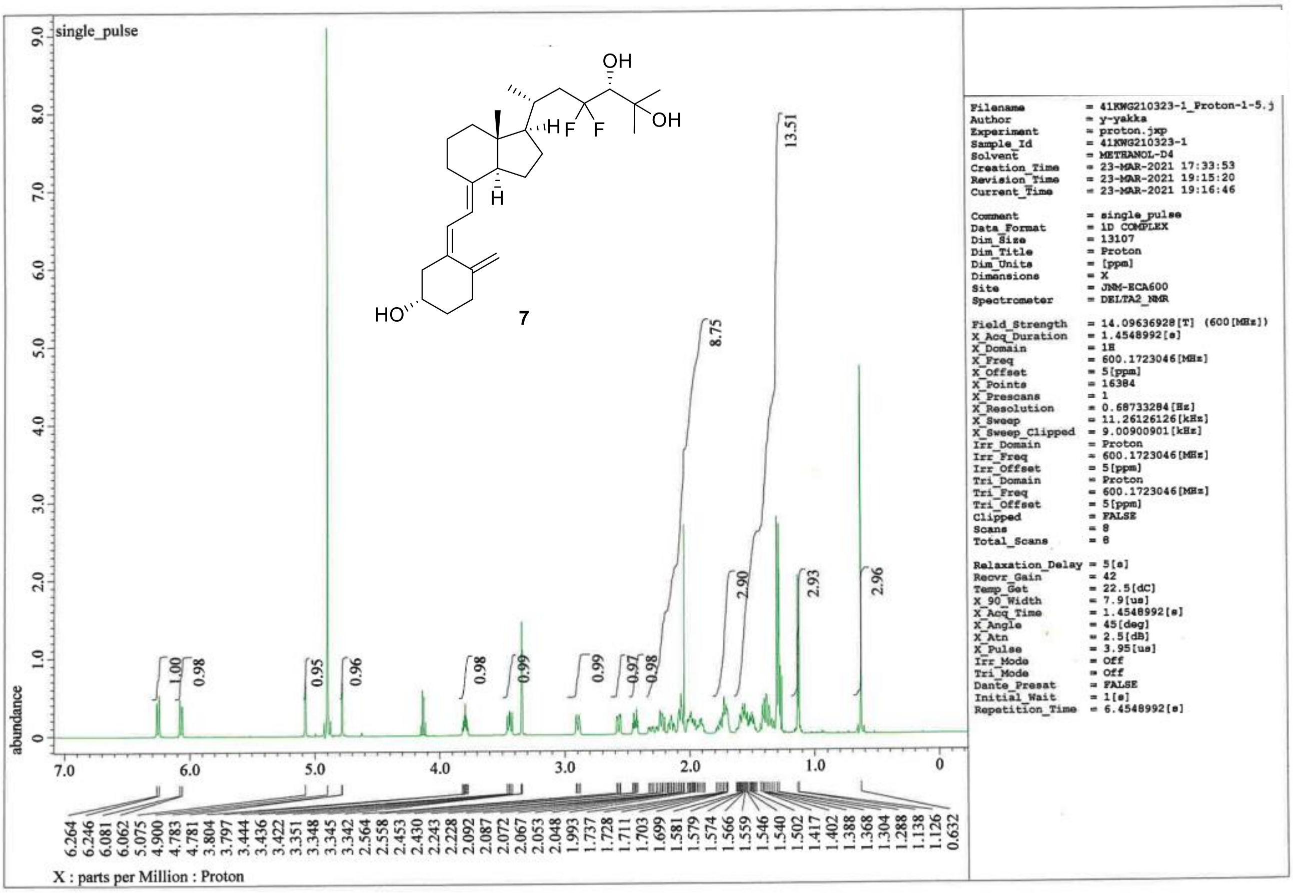
single_pulse
OH
H F F
OH
H
HO
7
abundance
X : parts per Million : Proton
1.00
0.98
0.95
0.96
0.98
0.99
0.99
0.97
0.98
8.75
13.51
2.90
2.93
2.96
6.264
6.246
6.081
6.062
5.075
4.900
4.783
4.781
3.804
3.797
3.444
3.436
3.422
3.351
3.348
3.345
3.342
2.564
2.558
2.453
2.430
2.243
2.228
2.092
2.087
2.072
2.067
2.053
2.048
1.993
1.737
1.728
1.711
1.703
1.699
1.581
1.579
1.574
1.566
1.559
1.546
1.540
1.502
1.417
1.402
1.388
1.368
1.304
1.288
1.138
1.126
0.632
Filename = 41KWG210323-1_Proton-1-5.j
Author = y-yakka
Experiment = proton.jxp
Sample_Id = 41KWG210323-1
Solvent = METHANOL-D4
Creation_Time = 23-MAR-2021 17:33:53
Revision_Time = 23-MAR-2021 19:15:20
Current_Time = 23-MAR-2021 19:16:46
Comment = single_pulse
Data_Format = 1D COMPLEX
Dim_Size = 13107
Dim_Title = Proton
Dim_Units = [ppm]
Dimensions = X
Site = JNM-ECA600
Spectrometer = DELTA2_NMR
Field_Strength = 14.09636928[T] (600[MHz])
X_Acq_Duration = 1.4548992[s]
X_Domain = 1H
X_Freq = 600.1723046[MHz]
X_Offset = 5[ppm]
X_Points = 16384
X_Prescans = 1
X_Resolution = 0.68733284[Hz]
X_Sweep = 11.26126126[kHz]
X_Sweep_Clipped = 9.00900901[kHz]
Irr_Domain = Proton
Irr_Freq = 600.1723046[MHz]
Irr_Offset = 5[ppm]
Tri_Domain = Proton
Tri_Freq = 600.1723046[MHz]
Tri_Offset = 5[ppm]
Clipped = FALSE
Scans = 8
Total_Scans = 8
Relaxation_Delay = 5[s]
Recvr_Gain = 42
Temp_Get = 22.5[dC]
X_90_Width = 7.9[us]
X_Acq_Time = 1.4548992[s]
X_Angle = 45[deg]
X_Atn = 2.5[dB]
X_Pulse = 3.95[us]
Irr_Mode = Off
Tri_Mode = Off
Dante_Presat = FALSE
Initial_Wait = 1[s]
Repetition_Time = 6.4548992[s]

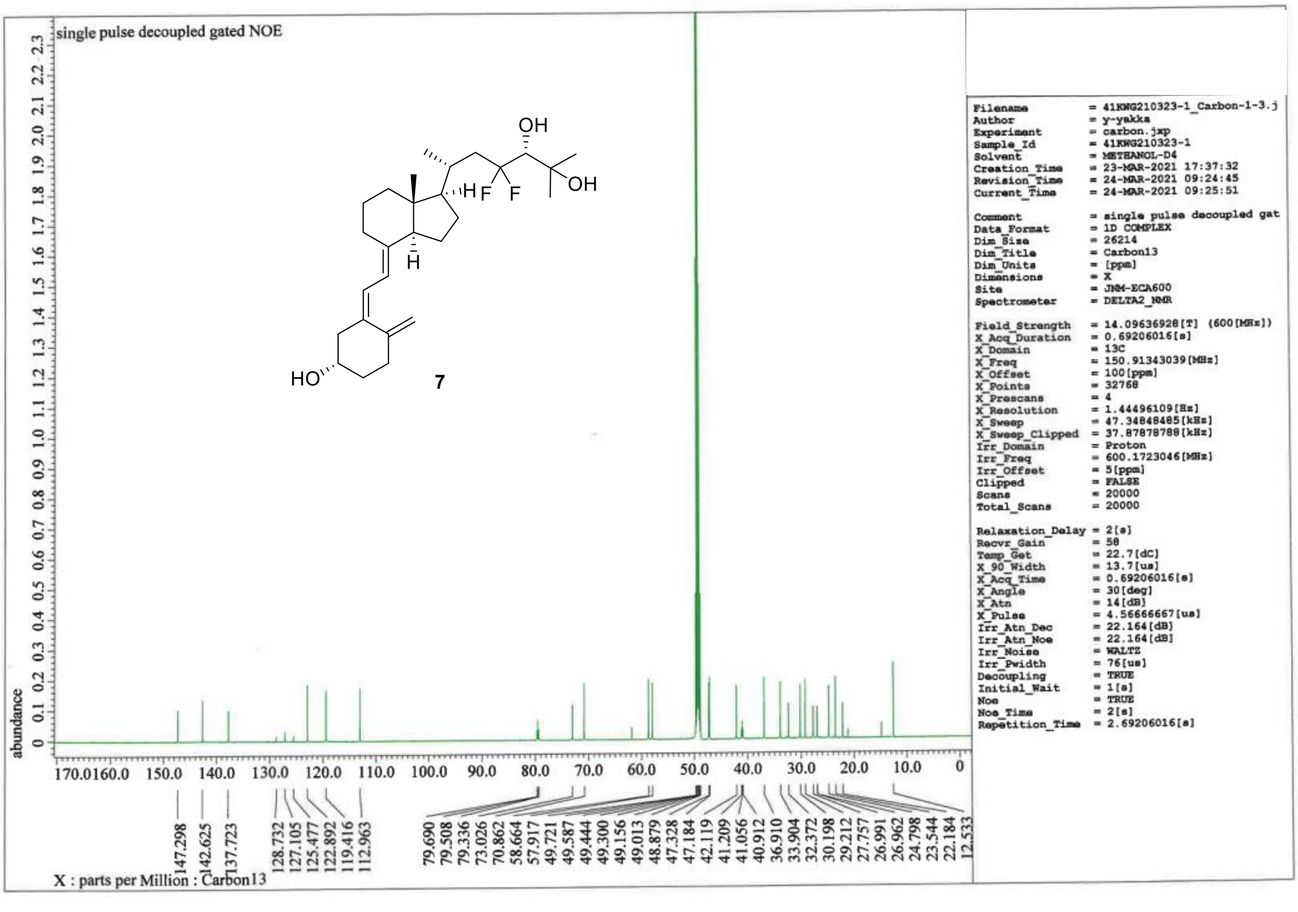
single pulse decoupled gated NOE
OH
H F F
OH
H
HO
7
abundance
X : parts per Million : Carbon13
147.298
142.625
137.723
128.732
127.105
125.477
122.892
119.416
112.963
79.690
79.508
79.336
73.026
70.862
58.664
57.917
49.721
49.587
49.444
49.300
49.156
49.013
48.879
47.328
47.184
42.119
41.209
41.056
40.912
36.910
33.904
32.372
30.198
29.212
27.757
26.991
26.962
24.798
23.544
22.184
12.533
Filename = 41KWG210323-1_Carbon-1-3.j
Author = y-yakka
Experiment = carbon.jxp
Sample_Id = 41KWG210323-1
Solvent = METHANOL-D4
Creation_Time = 23-MAR-2021 17:37:32
Revision_Time = 24-MAR-2021 09:24:45
Current_Time = 24-MAR-2021 09:25:51
Comment = single pulse decoupled gat
Data_Format = 1D COMPLEX
Dim_Size = 26214
Dim_Title = Carbon13
Dim_Units = [ppm]
Dimensions = X
Site = JNM-ECA600
Spectrometer = DELTA2_NMR
Field_Strength = 14.09636928[T] (600[MHz])
X_Acq_Duration = 0.69206016[s]
X_Domain = 13C
X_Freq = 150.91343039[MHz]
X_Offset = 100[ppm]
X_Points = 32768
X_Prescans = 4
X_Resolution = 1.44496109[Hz]
X_Sweep = 47.34848485[kHz]
X_Sweep_Clipped = 37.87878788[kHz]
Irr_Domain = Proton
Irr_Freq = 600.1723046[MHz]
Irr_Offset = 5[ppm]
Clipped = FALSE
Scans = 20000
Total_Scans = 20000
Relaxation_Delay = 2[s]
Recvr_Gain = 58
Temp_Get = 22.7[dC]
X_90_Width = 13.7[us]
X_Acq_Time = 0.69206016[s]
X_Angle = 30[deg]
X_Atn = 14[dB]
X_Pulse = 4.56666667[us]
Irr_Atn_Dec = 22.164[dB]
Irr_Atn_Noe = 22.164[dB]
Irr_Noise = WALTZ
Irr_Pwidth = 76[us]
Decoupling = TRUE
Initial_Wait = 1[s]
Noe = TRUE
Noe_Time = 2[s]
Repetition_Time = 2.69206016[s]

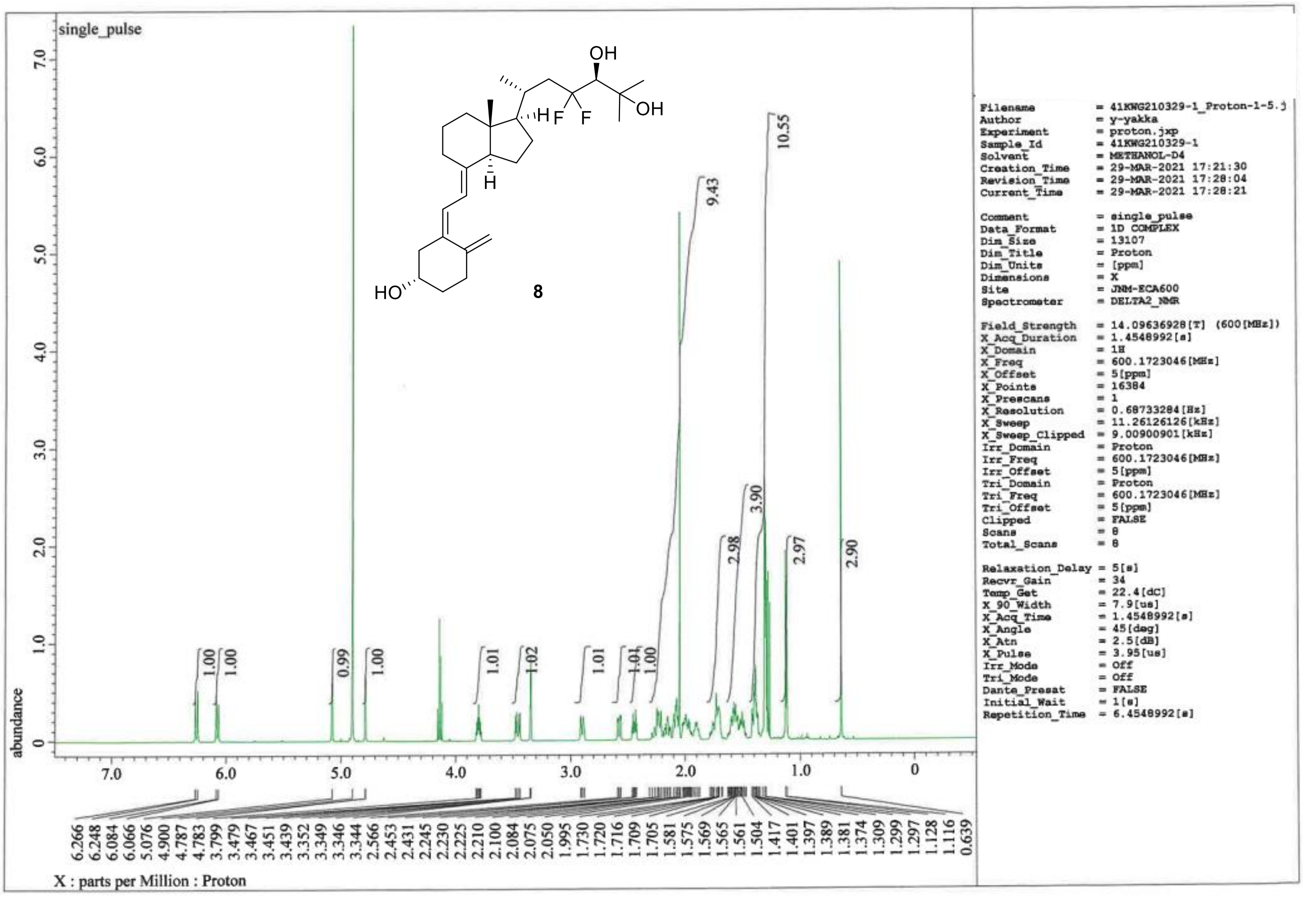
single_pulse
abundance
X : parts per Million : Proton
8
Filename = 41KWG210329-1_Proton-1-5.j
Author = y-yakka
Experiment = proton.jxp
Sample_Id = 41KWG210329-1
Solvent = METHANOL-D4
Creation_Time = 29-MAR-2021 17:21:30
Revision_Time = 29-MAR-2021 17:28:04
Current_Time = 29-MAR-2021 17:28:21
Comment = single_pulse
Data_Format = 1D COMPLEX
Dim_Size = 13107
Dim_Title = Proton
Dim_Units = [ppm]
Dimensions = X
Site = JNM-ECA600
Spectrometer = DELTA2_NMR
Field_Strength = 14.09636928[T] (600[MHz])
X_Acq_Duration = 1.4548992[s]
X_Domain = 1H
X_Freq = 600.1723046[MHz]
X_Offset = 5[ppm]
X_Points = 16384
X_Prescans = 1
X_Resolution = 0.68733284[Hz]
X_Sweep = 11.26126126[kHz]
X_Sweep_Clipped = 9.00900901[kHz]
Irr_Domain = Proton
Irr_Freq = 600.1723046[MHz]
Irr_Offset = 5[ppm]
Tri_Domain = Proton
Tri_Freq = 600.1723046[MHz]
Tri_Offset = 5[ppm]
Clipped = FALSE
Scans = 8
Total_Scans = 8
Relaxation_Delay = 5[s]
Recvr_Gain = 34
Temp_Get = 22.4[dC]
X_90_Width = 7.9[us]
X_Acq_Time = 1.4548992[s]
X_Angle = 45[deg]
X_Atn = 2.5[dB]
X_Pulse = 3.95[us]
Irr_Mode = Off
Tri_Mode = Off
Dante_Presat = FALSE
Initial_Wait = 1[s]
Repetition_Time = 6.4548992[s]

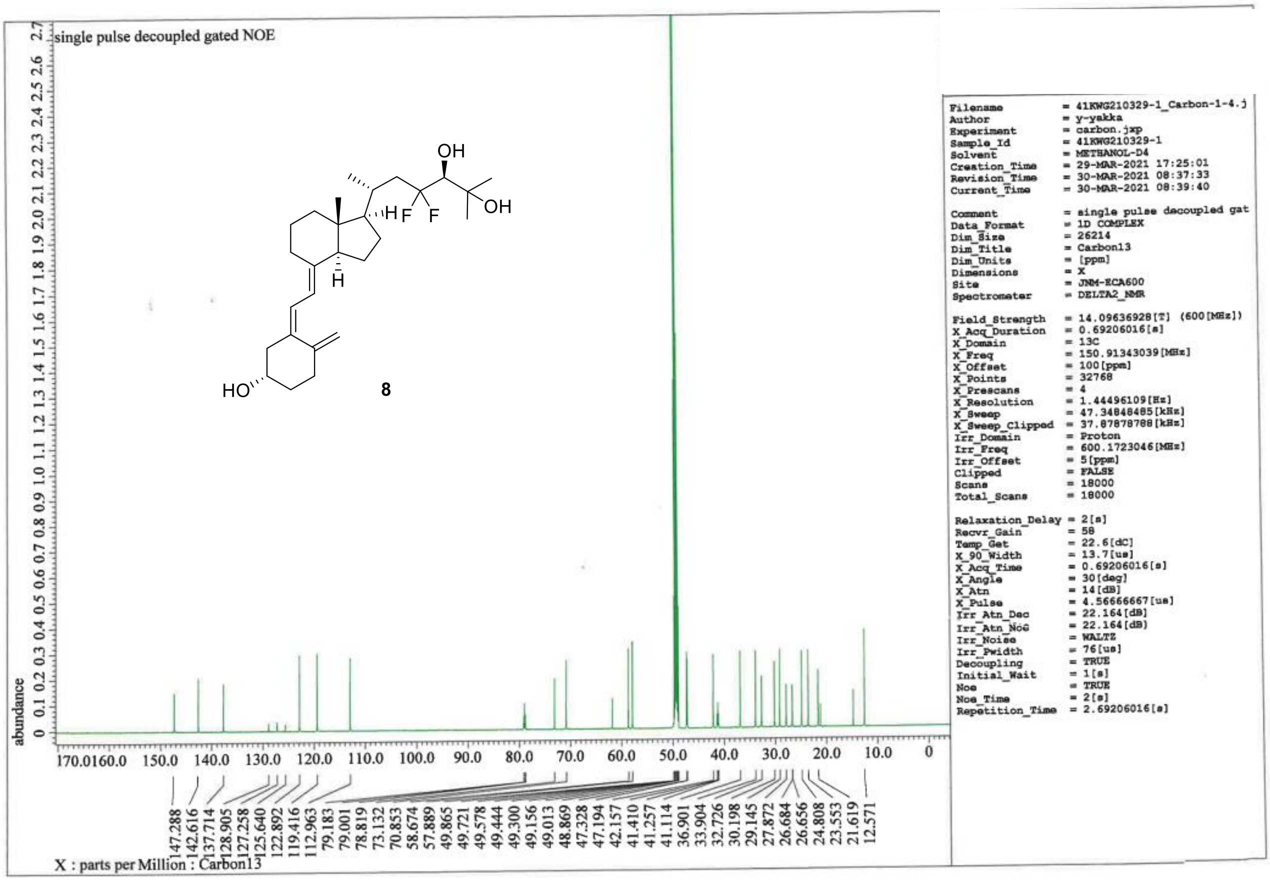
single pulse decoupled gated NOE
abundance
X : parts per Million : Carbon13
8
Filename = 41KWG210329-1_Carbon-1-4.j
Author = y-yakka
Experiment = carbon.jxp
Sample_Id = 41KWG210329-1
Solvent = METHANOL-D4
Creation_Time = 29-MAR-2021 17:25:01
Revision_Time = 30-MAR-2021 08:37:33
Current_Time = 30-MAR-2021 08:39:40
Comment = single pulse decoupled gat
Data_Format = 1D COMPLEX
Dim_Size = 26214
Dim_Title = Carbon13
Dim_Units = [ppm]
Dimensions = X
Site = JNM-ECA600
Spectrometer = DELTA2_NMR
Field_Strength = 14.09636928[T] (600[MHz])
X_Acq_Duration = 0.69206016[s]
X_Domain = 13C
X_Freq = 150.91343039[MHz]
X_Offset = 100[ppm]
X_Points = 32768
X_Prescans = 4
X_Resolution = 1.44496109[Hz]
X_Sweep = 47.34848485[kHz]
X_Sweep_Clipped = 37.87878788[kHz]
Irr_Domain = Proton
Irr_Freq = 600.1723046[MHz]
Irr_Offset = 5[ppm]
Clipped = FALSE
Scans = 18000
Total_Scans = 18000
Relaxation_Delay = 2[s]
Recvr_Gain = 58
Temp_Get = 22.6[dC]
X_90_Width = 13.7[us]
X_Acq_Time = 0.69206016[s]
X_Angle = 30[deg]
X_Atn = 14[dB]
X_Pulse = 4.56666667[us]
Irr_Atn_Dec = 22.164[dB]
Irr_Atn_Noe = 22.164[dB]
Irr_Noise = WALTZ
Irr_Pwidth = 76[us]
Decoupling = TRUE
Initial_Wait = 1[s]
Noe = TRUE
Noe_Time = 2[s]
Repetition_Time = 2.69206016[s]

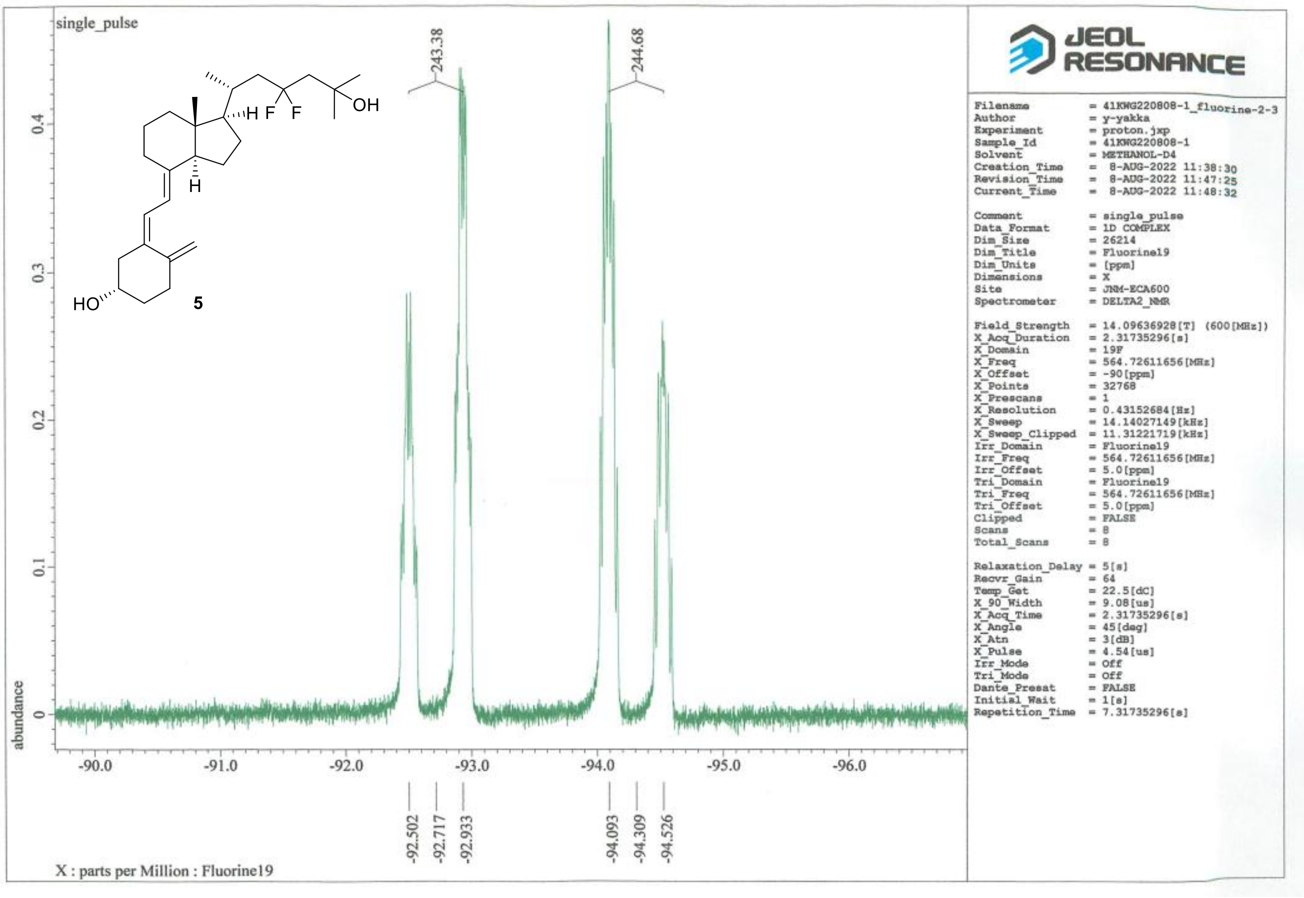
single_pulse
243.38
244.68
5
JEOL RESONANCE
Filename = 41KWG220808-1_fluorine-2-3
Author = y-yakka
Experiment = proton.jxp
Sample_Id = 41KWG220808-1
Solvent = METHANOL-D4
Creation_Time = 8-AUG-2022 11:38:30
Revision_Time = 8-AUG-2022 11:47:25
Current_Time = 8-AUG-2022 11:48:32
Comment = single_pulse
Data_Format = 1D COMPLEX
Dim_Size = 26214
Dim_Title = Fluorine19
Dim_Units = [ppm]
Dimensions = X
Site = JNM-ECA600
Spectrometer = DELTA2_NMR
Field_Strength = 14.09636928[T] (600[MHz])
X_Acq_Duration = 2.31735296[s]
X_Domain = 19F
X_Freq = 564.72611656[MHz]
X_Offset = -90[ppm]
X_Points = 32768
X_Prescans = 1
X_Resolution = 0.43152684[Hz]
X_Sweep = 14.14027149[kHz]
X_Sweep_Clipped = 11.31221719[kHz]
Irr_Domain = Fluorine19
Irr_Freq = 564.72611656[MHz]
Irr_Offset = 5.0[ppm]
Tri_Domain = Fluorine19
Tri_Freq = 564.72611656[MHz]
Tri_Offset = 5.0[ppm]
Clipped = FALSE
Scans = 8
Total_Scans = 8
Relaxation_Delay = 5[s]
Recvr_Gain = 64
Temp_Get = 22.5[dC]
X_90_Width = 9.08[us]
X_Acq_Time = 2.31735296[s]
X_Angle = 45[deg]
X_Atn = 3[dB]
X_Pulse = 4.54[us]
Irr_Mode = Off
Tri_Mode = Off
Dante_Presat = FALSE
Initial_Wait = 1[s]
Repetition_Time = 7.31735296[s]
-92.502
-92.717
-92.933
-94.093
-94.309
-94.526
X : parts per Million : Fluorine19
abundance

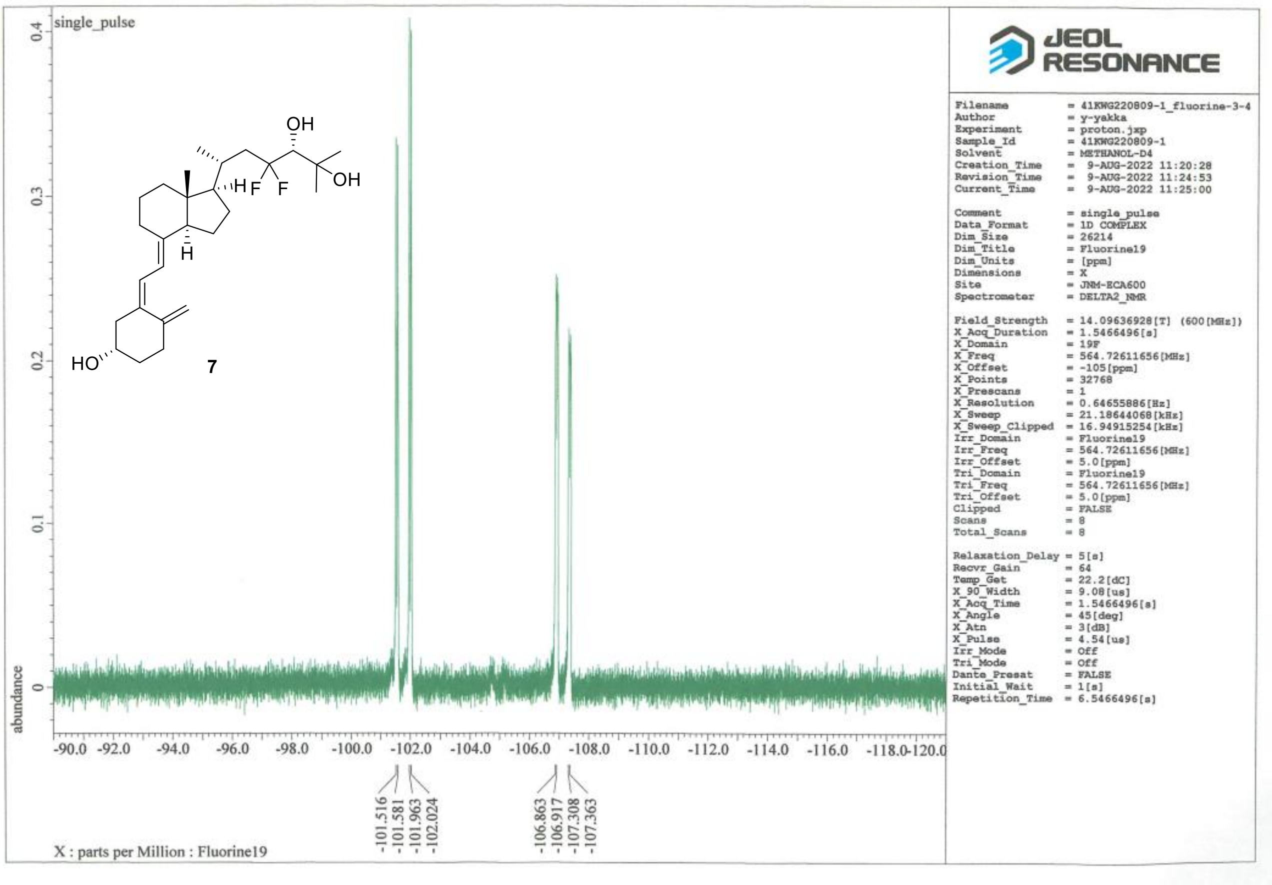
single_pulse
7
JEOL RESONANCE
Filename = 41KWG220809-1_fluorine-3-4
Author = y-yakka
Experiment = proton.jxp
Sample_Id = 41KWG220809-1
Solvent = METHANOL-D4
Creation_Time = 9-AUG-2022 11:20:28
Revision_Time = 9-AUG-2022 11:24:53
Current_Time = 9-AUG-2022 11:25:00
Comment = single_pulse
Data_Format = 1D COMPLEX
Dim_Size = 26214
Dim_Title = Fluorine19
Dim_Units = [ppm]
Dimensions = X
Site = JNM-ECA600
Spectrometer = DELTA2_NMR
Field_Strength = 14.09636928[T] (600[MHz])
X_Acq_Duration = 1.5466496[s]
X_Domain = 19F
X_Freq = 564.72611656[MHz]
X_Offset = -105[ppm]
X_Points = 32768
X_Prescans = 1
X_Resolution = 0.64655886[Hz]
X_Sweep = 21.18644068[kHz]
X_Sweep_Clipped = 16.94915254[kHz]
Irr_Domain = Fluorine19
Irr_Freq = 564.72611656[MHz]
Irr_Offset = 5.0[ppm]
Tri_Domain = Fluorine19
Tri_Freq = 564.72611656[MHz]
Tri_Offset = 5.0[ppm]
Clipped = FALSE
Scans = 8
Total_Scans = 8
Relaxation_Delay = 5[s]
Recvr_Gain = 64
Temp_Get = 22.2[dC]
X_90_Width = 9.08[us]
X_Acq_Time = 1.5466496[s]
X_Angle = 45[deg]
X_Atn = 3[dB]
X_Pulse = 4.54[us]
Irr_Mode = Off
Tri_Mode = Off
Dante_Presat = FALSE
Initial_Wait = 1[s]
Repetition_Time = 6.5466496[s]
-101.516
-101.581
-101.963
-102.024
-106.863
-106.917
-107.308
-107.363
X : parts per Million : Fluorine19
abundance

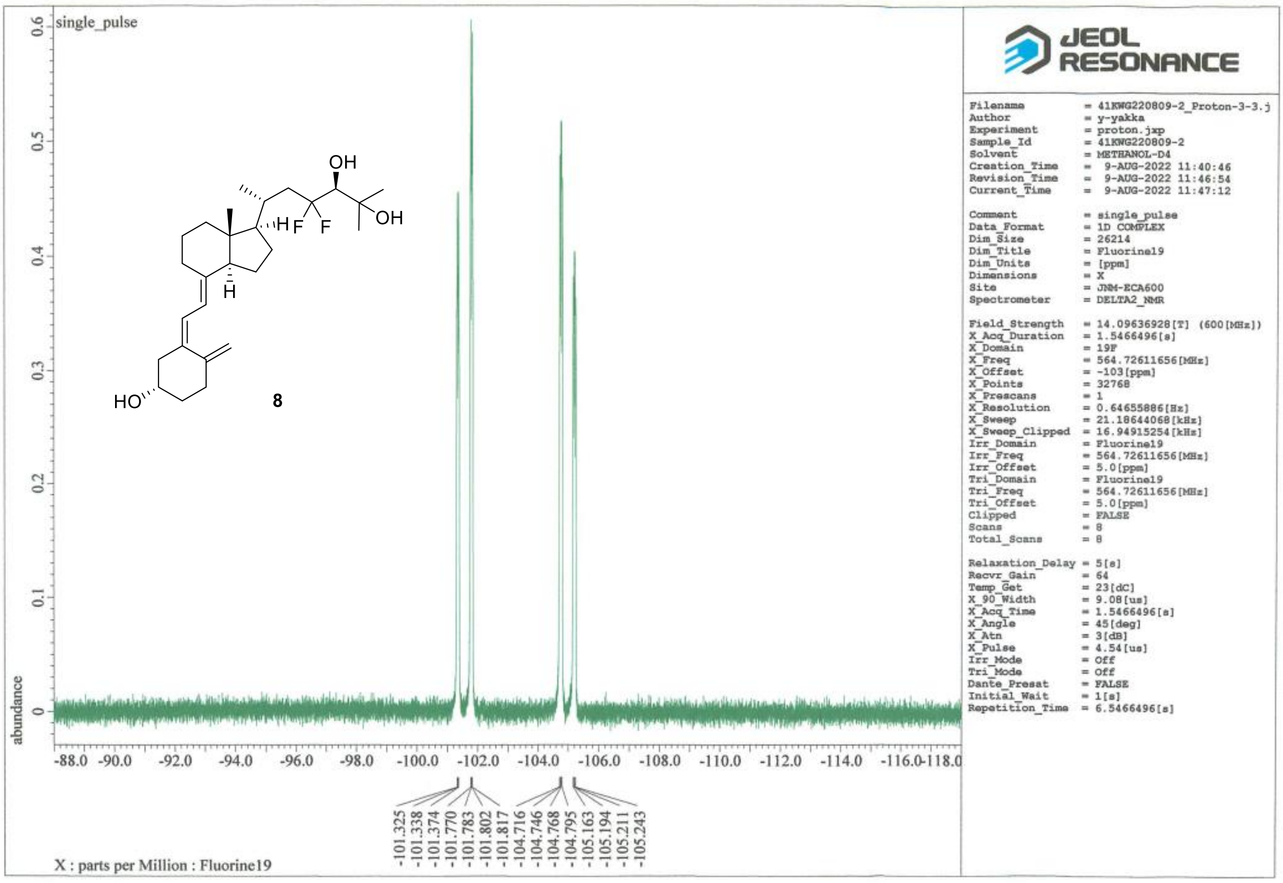
single_pulse
OH
OH
H
F F
H
HO
8
abundance
X : parts per Million : Fluorine19
-101.325
-101.338
-101.374
-101.770
-101.783
-101.802
-101.817
-104.716
-104.746
-104.768
-104.795
-105.163
-105.194
-105.211
-105.243
JEOL RESONANCE
Filename = 41KWG220809-2_Proton-3-3.j
Author = y-yakka
Experiment = proton.jxp
Sample_Id = 41KWG220809-2
Solvent = METHANOL-D4
Creation_Time = 9-AUG-2022 11:40:46
Revision_Time = 9-AUG-2022 11:46:54
Current_Time = 9-AUG-2022 11:47:12
Comment = single_pulse
Data_Format = 1D COMPLEX
Dim_Size = 26214
Dim_Title = Fluorine19
Dim_Units = [ppm]
Dimensions = X
Site = JNM-ECA600
Spectrometer = DELTA2_NMR
Field_Strength = 14.09636928[T] (600[MHz])
X_Acq_Duration = 1.5466496[s]
X_Domain = 19F
X_Freq = 564.72611656[MHz]
X_Offset = -103[ppm]
X_Points = 32768
X_Prescans = 1
X_Resolution = 0.64655886[Hz]
X_Sweep = 21.18644068[kHz]
X_Sweep_Clipped = 16.94915254[kHz]
Irr_Domain = Fluorine19
Irr_Freq = 564.72611656[MHz]
Irr_Offset = 5.0[ppm]
Tri_Domain = Fluorine19
Tri_Freq = 564.72611656[MHz]
Tri_Offset = 5.0[ppm]
Clipped = FALSE
Scans = 8
Total_Scans = 8
Relaxation_Delay = 5[s]
Recvr_Gain = 64
Temp_Get = 23[dC]
X_90_Width = 9.08[us]
X_Acq_Time = 1.5466496[s]
X_Angle = 45[deg]
X_Atn = 3[dB]
X_Pulse = 4.54[us]
Irr_Mode = Off
Tri_Mode = Off
Dante_Presat = FALSE
Initial_Wait = 1[s]
Repetition_Time = 6.5466496[s]
